# *NEK1* Variants in a Cohort of Italian Patients With Amyotrophic Lateral Sclerosis

**DOI:** 10.3389/fnins.2022.833051

**Published:** 2022-04-14

**Authors:** Nilo Riva, Laura Pozzi, Tommaso Russo, Giovanni Battista Pipitone, Paride Schito, Teuta Domi, Federica Agosta, Angelo Quattrini, Paola Carrera, Massimo Filippi

**Affiliations:** ^1^Neuropathology Unit, Division of Neuroscience, Institute of Experimental Neurology (INSPE), San Raffaele Scientific Institute, Milan, Italy; ^2^Neurology Unit, San Raffaele Scientific Institute, Milan, Italy; ^3^Neurorehabilitation Unit, San Raffaele Scientific Institute, Milan, Italy; ^4^Unit of Genomics for Human Disease Diagnosis, San Raffaele Scientific Institute, Milan, Italy; ^5^Neuroimaging Research Unit, Division of Neuroscience, Institute of Experimental Neurology (INSPE), San Raffaele Scientific Institute, Milan, Italy; ^6^Vita-Salute San Raffaele University, Milan, Italy; ^7^Neurophysiology Service, San Raffaele Scientific Institute, Milan, Italy

**Keywords:** motor neuron disease, frontotemporal dementia, neuromuscular diseases, neurodegenerative disorders, genetics, never in mitosis a (NIMA)-related kinase 1, flail arm, ALS phenotype

## Abstract

**Introduction:**

In the last few years, different studies highlighted a significant enrichment of *NEK1* loss of function (LoF) variants in amyotrophic lateral sclerosis (ALS), and an additional role for the p.Arg261His missense variant in the disease susceptibility. Several other missense variants have been described so far, whose pathogenic relevance remains however unclear since many of them have been reported in both patients and controls. This study aimed to investigate the presence of *NEK1* variants and their correlation with phenotype in a cohort of Italian patients with ALS.

**Methods:**

We sequenced a cohort of 350 unrelated Italian patients with ALS by next-generation sequencing (NGS) and then we analyzed the clinical features of *NEK1* carriers.

**Results:**

We detected 20 different *NEK1* rare variants (four LoF and 16 missense) in 33 unrelated patients with sporadic ALS (sALS). The four LoF variants (two frameshift and two splice-site variants) were all novel. The p.Arg261His missense variant was enriched in the patients’ cohort (*p* < 0.001). Excluding this variant from counting, the difference in the frequency of *NEK1* rare missense variants between patients and controls was not statistically significant. *NEK1* carriers had a higher frequency of flail arm (FA) phenotype compared with the other patients of the cohort (29.2% vs. 6.4%). Nine *NEK1* carriers (37.5%) also harbored variants in other ALS-related genes.

**Conclusion:**

This study confirms that *NEK1* LoF and p.Arg261. His missense variants are associated with ALS in an Italian ALS cohort and suggests a correlation between the presence of *NEK1* variants and FA phenotype.

## Introduction

Amyotrophic lateral sclerosis (ALS) is a fatal neurodegenerative disorder characterized by progressive loss of both upper and lower motor neurons, muscular weakness, and atrophy, leading to death within 2–5 years after diagnosis (Riva et al., 2016). It is largely recognized that the clinical spectrum of ALS is widely heterogeneous, with the definition of distinct subphenotypes, characterized by a different involvement of spinal, bulbar, and upper or lower motor neurons (Wijesekera et al., 2009; Chio et al., 2011). In particular, flail arm, flail leg, and pure lower motor neuron are phenotypes where predominant or selective lower motor neurons dysfunction occurs (Schito et al., 2020). About 10% of ALS cases are classified as familial (fALS), whereas the remaining 90% appear to be sporadic (sALS); in about two-thirds of fALS and up to 10% of sALS patients mutations in one or more specific ALS-related genes may be identified (Riva et al., 2016). To date, mutations in at least 30 genes have been implicated in ALS pathogenesis, and more than 120 genes have been proposed as potentially related to the disease. However, only a few of them, i.e., the *C9orf72* repeat expansion, *SOD1*, *FUS*, *TARDBP*, and *TBK1* genes, which account for up to 63% of fALS and 11% of sALS cases, have been clearly demonstrated to be implicated in the disease (Renton et al., 2014; Veldink, 2017).

The never in mitosis A (NIMA)-related kinase 1 (NEK1) is a widely studied serine/threonine kinase belonging to a family that shares significant homology with *Aspergillus nidulans* NIMA proteins. NEK1 plays a key role in several cellular functions, such as cell cycle progression, cilia regulation, DNA damage response (DDR), and mitochondrial membrane permeability (Peres de Oliveira et al., 2020). In addition, alterations in its expression have been correlated with human diseases like polycystic kidney disease and short-rib polydactyly syndrome (Type Majewski), Mohr syndrome, and Wilms tumor (Peres de Oliveira et al., 2020). Recently, large-scale whole-exome sequencing gene burden analysis studies highlighted a significant enrichment of *NEK1* loss of function (LoF) variants in ALS (Brenner et al., 2016; Kenna et al., 2016), and an additional role for the p.Arg261His missense variant for disease susceptibility (Nguyen et al., 2018). Several other missense variants have been described so far; however, their pathogenic relevance remains to be established, since many of them have been reported in both the patients with ALS and control cases (Kenna et al., 2016; Nguyen et al., 2018). In this context, a previous study demonstrated that *NEK1* LoF variants may induce the DNA damage accumulation in motor neurons derived from patients with ALS (Higelin et al., 2018). Other well-established ALS genes, such as *C9orf72* and *FUS*, were previously related to DDR mechanisms, a finding supporting the hypothesis that NEK1 may play a pathogenic role in ALS (Peres de Oliveira et al., 2020; Riancho et al., 2020). According to previous literature, *NEK1* variants do not affect the age at onset or survival in patients with ALS; while no clear genotype-phenotype correlation was reported, interestingly, a recent article described an association between *NEK1* LoF variants and hand involvement at onset in a cohort of Taiwanese patients with ALS (Tsai et al., 2020).

This study aimed to further investigate the presence and impact of *NEK1* variants and to explore potential genotype-phenotype correlations in a cohort of Italian patients with ALS.

## Materials and Methods

### Participants

In this study, we sequenced a cohort of 350 unrelated Italian patients with ALS (200 men and 150 women). Patients were enrolled at the Department of Neurology, San Raffaele Hospital, between 2014 and 2020. El Escorial revised criteria (Brooks et al., 2000) were used for ALS diagnostic categorization; no strict inclusion or exclusion criteria were adopted for patient selection (Brooks et al., 2000). Phenotypic classification of patients was assessed as previously described (Chio et al., 2011; Schito et al., 2020). We performed neuropsychological screening with Edinburgh Cognitive and Behavioral ALS Screen (Abrahams et al., 2014), followed by a further neuropsychological evaluation according to the diagnostic criteria for the behavioral variant of frontotemporal dementia and the ALSFTD consensus criteria (Rascovsky et al., 2011; Strong et al., 2017; Falzone et al., 2020).

The mean age of onset of our cohort was 57.4 ± 12.4 years; 296 patients (84.6%) had spinal onset ALS while 54 (15.4%) had bulbar onset ALS; twenty-nine patients (8.3%) had fALS. All patients were of Caucasian ethnicity, except three of Hispanic origin and the other three who were Africans. A cohort of 380 non-neurological unrelated Italian patients, for whom next-generation sequencing (NGS) exome sequencing data were available from our in-house database, was selected as the control group. This cohort ware screened to exclude the presence of neurological diseases or comorbidities.

### Standard Protocol Approvals, Registrations, and Patient Consents

This study was conducted according to Helsinki criteria and received approval from the Local Ethics Committee of the San Raffaele Hospital; blood samples were collected for diagnostic purposes and stored in our tissue bank, after informed consent, both for ALS and control cohorts.

### Genetic Analysis

Targeted NGS, using *TruSeq Neurodegeneration Panel* by Illumina (San Diego, CA, United States) was performed for *NEK1* mutational analysis, following the procedure of the manufacturer. Single variants reported in the FASTQ and VCF output file were analyzed with Illumina Variant Studio V3.0 software^1^ and visualized *via* Integrative Genome Viewer software.^2^ The *C9orf72* repeat expansion was also analyzed in all patients, using both amplicon-length and repeat-primed polymerase chain reactions, as described before (Agosta et al., 2017). All the identified *NEK1* variants found in patients with ALS and patients’ relatives were confirmed by Sanger sequencing (Pozzi et al., 2017). The clinical significance of reported variants was assessed based on the American College of Medical Genetics and Genomics (ACMG) guidelines (Richards et al., 2015). The Amyotrophic Lateral Sclerosis Online (ALSoD) database^3^ was used to select the 33 ALS-related genes to evaluate the oligogenicity of *NEK1* carriers. To exclude the presence of duplicates and to confirm that patients with ALS analyzed were unrelated, a KING tool analysis^4^ was performed, including a bcftools and plink2-based script (Manichaikul et al., 2010). The same analytical and validated in-house pipeline, and filtering criteria, were adopted for both the patients with ALS and control subjects (for detailed procedures and variants filtering criteria, see Supplementary Material).

### Statistical Analysis

Descriptive statistics are reported as count and percentage, for categorical variables, or mean and SD, for continuous variables. *NEK1* variants classified as likely benign were excluded from statistical analyses and counting, both in patients with ALS and control cases. Kaplan–Meier univariate analysis was carried out to determine the effect of *NEK1* variants on age at onset and survival (defined as the time from symptoms onset to death/tracheostomy). Follow-up data were censored on February 2021. The Fisher’s exact test and binary logistic regression analysis, adjusted for sex and age at onset, were used to explore differences in the frequency of patients carrying *NEK1* variants compared with controls, and the influence of *NEK1* variants on phenotype (Brenner et al., 2016; Black et al., 2017; Naruse et al., 2019; Tsai et al., 2020). Statistical significance was set at *p*-value < 0.05. All analyses were performed using the SPSS 22.0 software (Technologies Incorporation, Chicago, Illinois, United States).

## Results

We conducted an NGS analysis of 350 Italian patients with ALS. Overall, we detected and confirmed 20 different *NEK1* rare variants in 33 unrelated patients, all presenting with sALS. Four of these variants were LoF and 16 were missense; all of these variants were found in heterozygosity (Table 1). After excluding the variants classified as likely benign according to ACMG classification, both in ALS and control cohorts, we identified a total of 14 different rare variants (four LoF and 10 missense) in 24 patients and five missense in controls (Table 1 and Supplementary Table 3). Eight of the identified variants are novel, while the others have a minor allele frequency (MAF) < 0.0005. The only one variant with a higher MAF is the p.Arg261His (global MAF: 0.00385 and population MAF: 0.00398), which has already been reported as an ALS risk factor (Kenna et al., 2016; Nguyen et al., 2018; Lattante et al., 2021). Our cohort of patients with ALS was enriched for *NEK1* variants compared with controls (24/350, i.e., 6.86% and 6/380, i.e., 1.58%; *p* < 0.001).

**TABLE 1:** All the *NEK1* variants found in our NGS analysis.

cDNA change	Protein change	dbSNP ID^a^	ACMG	Global MAF^b^	Population MAF^c^	SIFT score	Polyphen score	Mutation taster pred	CADD	Nr.^d^ patients	Nr.^e^ controls
**Variants present only in patients with ALS**											
**LoF variants**											
c.1518-1519delTT	p.Leu507GlufsTer8	-	5	-	-	-	-	- (DC)	-	1	0
c.3222 + 1G > A	c.3222 + 1G > A^†^	-	4	-	-	-	-	-	-	1	0
c.3374 + 1G > A	c.3374 + 1G > A^†^	-	4	-	-	-	-	-	-	1	0
c.3759-3769delAAT AGTTCAAA	p.Ile1254TyrfsTer5	-	4	-	-	-	-	- (DC)	-	1	0
**Missense variants**											
c.337T > C	p.Cys113Arg	rs756261702	3	0.00004	0.00003	(0) D	(1) D	180 (DC)	28.9	1	0
c.380G > A	p.Arg127Gln	rs1312619422	3	0	0	(0) D	(1) D	43 (DC)	28.7	1	0
c.383A > T	p.Asp128Val	-	3	-	-	(0) D	(1) D	152 (DC)	-	1	0
c.469G > C	p.Val157Leu	-	3	-	-	(0.04) D	(0.059) B	32 (DC)	-	1	0
c.734A > G	p.Asp245Gly	rs756066992	3	0.000008	0.00001	(0.01) D	(0.004) B	94 (DC)	22.4	1	0
c.1978-1979delGAinsAT	p.Glu660Met	-	3	-	-	(0) D	(0.987) D	126 (DC)	-	1	0
c.2195A > G	p.Asn732Ser^‡^	rs755259769	2	0.00050	0	(0.49) T	(0.002) B	46 (P)	3.82	1	0
c.2375G > A	p.Gly792Asp^§^	rs200643637	3	0.00018	0.00010	(0.14) T	(0.559) P	94 (DC)	17.58	1	0
c.3278A > T	p.Asp1093Val	-	3	-	-	(0) T	(0.913) B	152 (DC)	-	1	0
c.3628G > A	p.Val1210Ile	rs1429056203	2	0.000004	0.000008	(0.91) T	(0.076) B	29 (P)	13.87	1	0
c.3664G > A	p.Glu1222Lys^¶^	rs1219156888	3	0.00050	0.00050	(0.01) D	(1) D	56 (DC)	27.6	1	0
**Variants present in patients with ALS and controls**											
c.782G > A	p.Arg261His^§^	rs200161705	4	0.00385	0.00398	(0.01) D	(0.987) D	29 (DC)	25.1	13	1
c.1942A > G	p.Lys648Glu^§^	rs371562840	2	0.00050	0.00050	(0.02) D	(0.967) D	56 (DC)	26.5	1	1
c.2235T > G	p.Asn745Lys^§^	rs34324114	2	0.00382	0.00399	(0) D	(1) D	94 (DC)	24.8	3	6
c.2731C > G	p.Gln911Glu^‡^	rs6828134	2	0.00018	0	(0.19) T	(0.005) B	29 (DC)	13.97	2	1
c.3289G > A	p.Val1097Ile^‡^	rs374890006	2	0	0	(0.08) T	(0.03) B	29 (P)	13.26	1	1

*^a^dbSNP150; ^b^Global MAF, global allele counts were calculated from all subjects in the GnomAD database; ^c^MAF population, population allele count refers to European Ancestry subjects from the GnomAD database; ^d^Nr. Patients, number of patients with ALS carrying the variant. ^e^Nr. Controls, number of controls carrying the variant. Key: ^†^variants predicted as splice site by in silico tools; ^‡^variants already described in literature in control cases; ^§^variants already described in literature in both patients with ALS and control cases; ^¶^variants already described in literature in patients with ALS (see Supplementary Table 5 for details). ACMG, American College of Medical Genetics and Genomics; ACMG classification: 1, benign; 2, likely benign; 3, uncertain significance; 4, likely pathogenic; 5, pathogenic; MAF, minor allele frequency; SIFT, T = tolerated and D = deleterious; Polyphen, B = benign, P = possibly damaging, D = damaging; Mutation Taster, D = disease causing, N = polymorphism, A = disease causing automatic; CADD, combined annotation-dependent depletion; CADD scores, scaled CADD scores (Phred like) for scoring deleteriousness.*

LoF variants were found in 1.14% (4/350) of patients and none (0/380) in control individuals, in line with previous literature (Brenner et al., 2016; Kenna et al., 2016; Nguyen et al., 2018; Shu et al., 2018; Naruse et al., 2019; Tsai et al., 2020; Lattante et al., 2021), even though this difference did not reach statistical significance. In addition, we also observed a higher frequency of *NEK1* missense variants in patients vs. controls (21/350, i.e., 6.0% and 6/380, i.e., 1.58%; *p* = 0.0015). However, this enrichment was driven by the high prevalence of the p.Arg261His, found in 13 patients with ALS and only one control (*p* < 0.001). Excluding this latter from counting, the difference between the two groups was not statistically significant (2.57% patients with ALS and 1.32% controls), in line with previous studies (Brenner et al., 2016; Kenna et al., 2016; Nguyen et al., 2018; Shu et al., 2018; Naruse et al., 2019; Tsai et al., 2020; Lattante et al., 2021).

Patients with ALS carrying *NEK1* variants did not differ for sex distribution, age at onset, or survival from the other patients of the cohort. However, a binary logistic regression analysis, adjusted for sex and age at onset, revealed a significantly higher risk for *NEK1* carriers to manifest the flail arm phenotype (OR 5.34, 95% CI 1.93–14.74). Indeed, we found a significant enrichment of patients presenting with a flail arm phenotype among the *NEK1* carriers group (7/24, i.e., 29.2% vs. 21/326, i.e., 6.4%, *p* = 0.0013).

### *NEK1* Loss of Function Variants

The four *NEK1* LoF were absent from all the public genomic databases, including dbSNP, and also from our in-house control cohort (Table 1). They include two frameshift variants (p.Leu507GlufsTer8, p.Ile1254TyrfsTer5) and the two c.3222 + 1G > A and c.3374 + 1G > A variants, which are predicted to alter the normal splice sites from *in silico* tools (Supplementary Table 2).

The p.Leu507GlufsTer8 is predicted to cause the transcript degradation through nonsense-mediated decay, while the p.Ile1254TyrfsTer5, being at the C-terminal of the protein, could likely lead to the formation of a truncated protein. These frameshift variants were identified in two unrelated male patients with sALS, both presenting with a flail arm phenotype (Table 2).

**TABLE 2:** Demographic and clinical characteristics of patients with *NEK1* variants.

Pt-ID^a^	Variant	Sex	Age of onset (years)	Family history	Disease duration (months)^†^	ALSFRS-R progression rate^‡^	Site of onset	Phenotype
ALS_41	p.Leu507GlufsTer8^§^	M	57	sALS	48	0.14	Upper limbs	Flail arm
ALS_798	c.3222 + 1G > A + p.Asp1093Val^§^	F	74	sALS	53	0.15	Upper limbs	Flail arm
ALS_716	c.3374 + 1G > A	F	53	sALS	22	2.00	Bulbar	Bulbar
ALS_293	p.Ile1254TyrfsTer5	M	54	sALS	52	0.32	Upper limbs^¶^	Flail arm
ALS_315	p.Cys113Arg^§^	F	57	sALS	44	0.56	Upper limbs^¶^	Classic
ALS_119	p.Arg127Gln^§^	F	69	sALS	73	0.14	Upper limbs^¶^	Flail arm
ALS_695	p.Asp128Val + p.Arg261His^§^	M	53	sALS	23	-	Upper limbs	Classic
ALS_367	p.Val157Leu	F	61	sALS	53	0.35	Bulbar	Bulbar
ALS_549	p.Asp245Gly	F	74	sALS	65	0.44	Lower limbs	Pumn
ALS_152	p.Arg261His	M	64	sALS	131	0.11	Upper limbs	Flail arm
ALS_155	p.Arg261His^§^	M	65	sALS	29	1.53	Lower limbs	Plmn
ALS_200	p.Arg261His	F	57	sALS	82	0.26	Lower limbs	Flail leg
ALS_209	p.Arg261His	F	71	sALS	38	0.91	Lower limbs	Classic
ALS_444	p.Arg261His^§^	F	67	sALS	75	0.58	Upper limbs	Flail arm
ALS_464	p.Arg261His	F	45	sALS	52	0.33	Upper limbs^¶^	Classic
ALS_480	p.Arg261His^§^	F	80	sALS	36	1.38	Upper limbs^¶^	Classic
ALS_512	p.Arg261His^§^	M	63	sALS	25	0.83	Upper Limbs	Flail arm
ALS_839	p.Arg261His	M	72	sALS	25	0.40	Lower limbs	Flail leg
ALS_855	p.Arg261His	M	67	sALS	28	0.56	Lower limbs	Flail leg
ALS_883	p.Arg261His	M	73	sALS	32	0.40	Bulbar	Bulbar
ALS_974	p.Arg261His	M	56	sALS	21	0.15	Lower limbs	Pumn
ALS_742	p.Lys648Glu	F	66	sALS	51	1.36	Lower limbs	Pyramidal/FTD
ALS_101	p.Glu660Met	M	48	sALS	46	0.77	Upper limbs	Classic
ALS_190	p.Asn732Ser	F	41	sALS	226	0.15	Lower limbs	Pumn/FTD
ALS_320	p.Asn745Lys	F	54	sALS	107	0.25	Upper limbs^¶^	Flail arm
ALS_470	p.Asn745Lys	M	50	sALS	166	0.33	Lower limbs	Plmn
ALS_945	p.Asn745Lys	F	33	sALS	15	0.63	Upper limbs	Classic
ALS_66	p.Gly792Asp	M	67	sALS	110	0.18	Lower limbs	Flail leg
ALS_441	p.Gln911Glu	M	85	sALS	34	1.17	Upper limbs^¶^	Classic
ALS_862	p.Gln911Glu	M	42	sALS	13	1.67	Bulbar	Bulbar
ALS_751	p.Val1097Ile	M	53	sALS	41	0.25	Lower limbs	Flail leg
ALS_601	p.Val1210Ile	M	57	sALS	10	3.14	Lower limbs	Classic/FTD
ALS_518	p.Glu1222Lys	M	59	sALS	45	0.74	Lower limbs	Flail leg

*^a^PT-ID, patient identification code. ^†^Disease duration: time from disease onset to latest visit or death. ^‡^ALSFRS-R, ALS Functional Rating Scale-revised. Progression rate = (48-ALSFRS-R score)/time from disease onset to ALSFRS-R; ^§^NEK1 carriers with other variants of interest (see Table 3); ^¶^hand onset. Key, sALS, sporadic amyotrophic lateral sclerosis; Fals, familial amyotrophic lateral sclerosis; plmn, predominantly lower motor neuron disease; pumn, predominantly upper motor neuron disease.*

The c.3222 + 1G > A splice site was found in a female patient with sALS also presenting with a flail arm phenotype. Interestingly, this patient also carried a novel *NEK1* missense variant (Tables 1, 2). The c.3374 + 1G > A variant was identified in a female patient with sALS presenting with a bulbar phenotype (Table 2); this variant was also detected in her unaffected brother, whose DNA was available for testing (Family #1 in Supplementary Figure 1).

### *NEK1* Missense Variants

Overall, we found 10 different missense variants in 22 unrelated patients with ALS (Table 1). The most frequent missense variant identified in this study was the p.Arg261His, previously described as ALS-risk factor (Kenna et al., 2016; Nguyen et al., 2018; Lattante et al., 2021), that we found in 13 patients with ALS and one control (3.71 and 0.26%, respectively). Nine missense variants were classified as variants of uncertain significance (VUS), according to the ACMG classification. Among them, the p.Asp128Val, p.Val157Leu, p.Glu660Met, and p.Asp1093Val were novel, neither reported in the literature or dbSNPs database nor found in our in-house controls. The two patients with the novel p.Asp128Val and p.Asp1093Val were both found to carry another *NEK1* variant (p.Arg261His and c.3222 + 1G > A, respectively) (Table 3). These patients had a classic and a flail arm phenotype, respectively. The p.Glu660Met variant was the result of the change of two adjacent bases (c.1978-1979delGAinsAT). The other five missense variants classified as VUS were found only in the ALS cohort; among them, the p.Glu1222Lys was previously described in a patient with ALS (Lattante et al., 2021), the p.Gly792Asp in both patients with ALS and controls (Kenna et al., 2016), whereas the others were only reported in dbSNPs database. The other six missense variants were considered as likely benign (Table 1).

**TABLE 3:** List of *NEK1* carriers with other variants of interest.

Pt ID^a^	Variant 1 (ACMG)	Variant 2 (ACMG)	Variant 3 (ACMG)
ALS_41	*NEK1* p.Leu507GlufsTer8 (5)	*FIG4* p.Pro344Thr (4)	
ALS_798	*NEK1* c.3222 + 1G > A (4)	*NEK1* p.Asp1093Val (3)	
ALS_315	*NEK1* p.Cys113Arg (3)	*C9Orf72* expansion (5)	
ALS_119	*NEK1* p.Arg127Gln (3)	*SETX* p.Leu223Val (3)	*EPHA4* p.Gly383Glu (3)
ALS_695	*NEK1* p.Asp128Val (3)	*NEK1* p.Arg261His (4)	*SOD1* p.Asp91Ala (5)
ALS_444	*NEK1* p.Arg261His (4)	*C9Orf72* expansion (5)	*ANG* p.Ile70Val (4)
ALS_155	*NEK1* p.Arg261His (4)	*TARDBP* p.Ala382Thr (5)	*FIG4* p.Ile262SerfsTer10 (3)
ALS_512	*NEK1* p.Arg261His (4)	*TARDBP* p.Gly368Ser (4)	*GRN* p.Pro21Ser (3)
ALS_480	*NEK1* p.Arg261His (4)	*UNC13A* p.Leu1669Pro (3)	

*^a^PT-ID, patient identification code. Key: American College of Medical Genetics and Genomics (ACMG) classification: 1, benign; 2, likely benign; 3, uncertain significance; 4, likely pathogenic; and 5, pathogenic. Benign and likely benign variants are not shown.*

### *NEK1* Polygenic Carriers

We next investigated whether the *NEK1* patients with ALS also carried other variants of interest among other 33 *ALS* genes (Supplementary Methods). We found that nine patients (37.5%) were oligogenic for other *ALS* genes (Table 3); in particular, two carried the pathological expansion of the *C9orf72* gene and one of them carried also the p.Ile70Val missense in the *ANG* gene, which was classified as presumed pathogenic (Greenway et al., 2006; Crabtree et al., 2007; Paubel et al., 2008). The patient carrying the *NEK1* p.Cys113Arg missense, classified as VUS, also harbored the *C9orf72* expansion, while her unaffected mother and brother carried only the *NEK1* variant (Family #2 in Supplementary Figure 1). This patient had hand onset and a classic ALS phenotype (Table 2). The other four patients with the *C9orf72* expansion carried likely benign *NEK1* missense variants and were excluded from counting. Notably, the two unaffected sisters of the patient in Family #3 carried the *C9orf72* expansion but were wild type for the *NEK1* p.Asn732Ser likely benign variant, identified in their affected sister (Supplementary Figure 1).

Two p.Arg261His *NEK1* carriers were also heterozygous for a *TARDBP* pathogenic variant (p.Ala382Thr and p.Gly368Ser, respectively). The unaffected brother of the patient carrying both the p.Arg261His *NEK1* risk factor and the likely pathogenic p.Gly368Ser *TARDBP* variant was found to carry the *TARDBP* variant but not the *NEK1* missense (Family #4 in Supplementary Figure 1). The patient had a flail arm phenotype (Table 2).

One patient carried also the *SOD1* p.Asp91Ala pathogenic variant; notably, he was found to carry the p.Arg261His risk factor and another *NEK1* missense variant (p.Asp128Val) (Table 3).

Another patient, presenting with a flail arm phenotype, carried two different *NEK1* variants: the c.3222 + 1G > A LoF and the novel p.Asp1093Val missense variant (Table 2).

The other three patients carried a VUS in *EPHA4, FIG4, SETX*, and *UNC13A* ALS-related genes (Table 3).

*NEK1* polygenic carriers did not show relevant differences in age of onset or survival compared with ALS patients carrying a single *NEK1* variant (data not shown).

## Discussion

This study describes the genetic variability of the *NEK1* gene in a cohort of 350 Italian patients with ALS. Indeed, we identified four LoF and 16 missense variants in 33 unrelated patients with sALS. We also characterized the clinical features of the patients carrying *NEK1* variants, highlighting a potential association with the flail arm phenotype.

*NEK1* LoF variants have been previously proposed to play a pathogenic role in ALS, since several studies described a significantly higher frequency in both patients with fALS and sALS compared with controls, with a prevalence ranging from 0.4 to 1.8% (Brenner et al., 2016; Kenna et al., 2016; Nguyen et al., 2018; Shu et al., 2018; Naruse et al., 2019; Tsai et al., 2020; Lattante et al., 2021). A previous study also demonstrated that LoF variants lead to NEK1 haploinsufficiency, resulting in DDR impairment (Higelin et al., 2018), a mechanism already associated with other ALS-related genes (Riancho et al., 2020). In our ALS cohort, LoF frequency was 1.1%. However, *NEK1* LoF variants should not be considered rare: indeed, despite being less frequent than variants in *C9orf72, SOD1, FUS*, *TARDBP*, and *TBK1*, their prevalence is higher compared with other ALS-related genes (Tsai et al., 2020).

In addition to LoF, we also observed an apparently enriched fraction of *NEK1* missense variants in patients with ALS with an overall frequency of 6.0% compared with 1.58% of our in-house controls. Previous studies reported, instead, similar percentages of missense variants in both the ALS and control cases, ranging from 1.1 to 3.7%, suggesting that, collectively, missense variants might not affect ALS susceptibility (Supplementary Tables 4, 5; Brenner et al., 2016; Kenna et al., 2016; Black et al., 2017; Gratten et al., 2017; Nguyen et al., 2018; Shu et al., 2018; Naruse et al., 2019; Tsai et al., 2020; Lattante et al., 2021). The enrichment we observed is explained by the higher prevalence of the p.Arg261His missense in our ALS cohort, which was 10 times more frequent than in controls. The p.Arg261His is the only *NEK1* missense variant already reported to be related to an increased risk for ALS (Kenna et al., 2016; Black et al., 2017; Nguyen et al., 2018; Lattante et al., 2021). Nevertheless, previous studies showed p.Arg261His frequency in patients with ALS ranging from 0.6 to 1.7% (Kenna et al., 2016; Nguyen et al., 2018; Lattante et al., 2021), while our data reported a higher frequency of 3.7%. Regarding other *NEK1* missense variants, further studies are required to assess their role in ALS pathogenesis.

We then explored whether *NEK1* variants in patients with ALS might have an association with specific phenotypic features. Notably, 29.2% of *NEK1* carriers presented with a flail arm phenotype, a percentage significantly higher compared with non-*NEK1* carriers of our ALS cohort, and with the 11% prevalence of this phenotype reported by previous studies (Wijesekera et al., 2009; Chio et al., 2011; Schito et al., 2020). Among patients with ALS carrying *NEK1* LoF variants, three presented with a flail arm phenotype, while the patient whose variant was shared with her unaffected brother had instead a bulbar phenotype. Interestingly, a recent report correlated the presence of a *NEK1* LoF variant with a hand-onset ALS in a Taiwanese ALS cohort, reporting it in all LoF carriers (Tsai et al., 2020). Nevertheless, other studies described *NEK1* LoF carriers with the same clinical presentation, even if the authors did not directly point out such correlation (Brenner et al., 2016; Shu et al., 2018; Naruse et al., 2019).

Nowadays it is well recognized that ALS and frontotemporal dementia (FTD) share common clinical and genetic features and could represent a pathological *continuum* (Liscic et al., 2020). In our study, none of the *NEK1* LoF carriers had cognitive impairment; moreover, we found three patients carrying a *NEK1* likely benign missense variant that presented also with FTD, but all of them carried also the *C9orf72* expansion. Intriguingly, none of the patients with ALS carrying *NEK1* LoF variants described up to date had cognitive impairment, except for one case (Lattante et al., 2021), whereas *C9orf72* association to ALS-FTD is well known (Hodges, 2012).

It has already been proposed that ALS could be a complex multistep pathogenic process, in which the disease presentation results from the combination of different factors (Al-Chalabi et al., 2014; Scarlino et al., 2020). This “multiple-hit hypothesis” could account for the phenotypic heterogeneity observed even in patients harboring the same gene mutation, and for the reduced penetrance of *NEK1* variants (Nguyen et al., 2018; Scarlino et al., 2020). Our results are in line with this oligogenic hypothesis since more than one-third of *NEK1* carriers had variants in other ALS-related genes. From pedigree analysis, we found that in two different families the patients with ALS carried both the *C9orf72* expansion and a *NEK1* missense variant, whereas the unaffected relatives carried a variant in only one of these two genes. Similarly, in another family, both the patient and his unaffected brother carried a *TARDBP* mutation, but only the patient was oligogenic for a *NEK1* missense variant. Taken together, these observations support the already proposed hypothesis that *NEK1* variants may confer a significant susceptibility to ALS, even though they may be not sufficient *per se* for disease development, possibly acting as a phenotypic modifier (Nguyen et al., 2018; Lattante et al., 2021). Although several studies already suggested that additional variants may affect ALS disease severity (Pang et al., 2017; Ross et al., 2020), in our cohort the concomitant presence of a *NEK1* variant with variants in other ALS-related genes did not seem to significantly influence the disease onset or progression rate.

We acknowledge that this study has some limitations. First, our sample size is relatively small and this cannot be considered a population-based study. However, we were able to provide detailed phenotype information of patients with ALS to test potential genotype-phenotype correlations. Second, it is difficult to determine the potential pathogenic role for the missense variants. Indeed, specific functional studies are needed to evaluate whether the single amino acid change could affect the whole protein function. In our study, the lack of biological material from *NEK1* carriers prevented us to perform exhaustive segregation analysis and functional studies at the transcript or protein levels. However, we analyzed also a non-neurological age-matched cohort of controls.

## Conclusion

In conclusion, this study supports *NEK1*’s contribution to ALS pathogenesis in the Italian population. We confirmed previous findings that *NEK1* LoF variants, and the p.Arg261His missense variant, are significantly enriched in patients with ALS. Notably, our data also suggest an association of the flail arm phenotype with the presence of *NEK1* variants. We also found a relevant number of *NEK1* carriers with variants in other ALS-related genes, supporting the hypothesis that *NEK1* variants might concur to ALS pathogenesis in an oligogenic model of disease, possibly acting as a phenotypic modifier.

## Data Availability Statement

The data presented in the study are deposited in the NCBI SRA Sequence Read Archive Depository, accession number PRJNA817104.

## Ethics Statement

The studies involving human participants were reviewed and approved by the Local Ethics Committee of the San Raffaele Hospital. The patients/participants provided their written informed consent to participate in this study.

## Author Contributions

NR, PC, and AQ conceived and designed the study. NR, LP, TR, GP PS, TD, FA, PC, and AQ contributed to data collection, analysis, and interpretation and generation of the tables. NR, LP, and TR did the statistical analysis. NR, TR, and PS were involved in patient selection and clinical data collection. MF, PC, and AQ supervised the study. LP and TR wrote the first draft of the article. All authors actively contributed to the writing, critically reviewing of the article for important intellectual content, and approved the final version and contributed to data interpretation.

## Conflict of Interest

The authors declare that the research was conducted in the absence of any commercial or financial relationships that could be construed as a potential conflict of interest.

## Publisher’s Note

All claims expressed in this article are solely those of the authors and do not necessarily represent those of their affiliated organizations, or those of the publisher, the editors and the reviewers. Any product that may be evaluated in this article, or claim that may be made by its manufacturer, is not guaranteed or endorsed by the publisher.

## Footnotes

1
http://variantstudio.software.illumina.com/

2
http://www.broadinstitute.org/software/igv/

3
https://alsod.ac.uk/

4
https://kingrelatedness.com
