# Supplementary material for: NEK1 Variants in a Cohort of Italian Patients With Amyotrophic Lateral Sclerosis
Source: Front Neurosci. 2022 Apr 14;16:833051. doi: 10.3389/fnins.2022.833051 (PMC9048593; doi:10.3389/fnins.2022.833051)
Supplement: Supplementary file 1 [file Data_Sheet_1.docx]

**
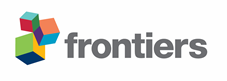
**

**Supplementary Material**

***Supplementary Methods***

*DNA Preparation*

Genetic analysis was performed on genomic DNA extracted from peripheral EDTA-treated blood samples using the NucleoSpin Blood L Extraction Kit (Macherey-Nagel, Düren, Germany) following the manufacturer’s instructions. The quality of genomic DNA was evaluated and quantified by NanoDrop ND-1000 spectrophotometer (Thermo Fisher Scientific,Wilmington, DE) and Qubit 2.0 Fluorometer using the Qubit dsDNA BR assay (Invitrogen, Merelbeke, Belgium). Only high-quality DNA samples (1.8–2.0 260/280 ratio and 2.0–2.2 260/230 ratio) were used for NGS analysis.

*Targeted Next Generation Sequencing (NGS)*

We used the targeted next-generation sequencing (NGS) *TruSeq Neurodegeneration Panel* by Illumina (San Diego, CA, USA), following the manufacturer’s procedure. The panel includes both risk-validated genes and genes thought to be associated with the major neurodegenerative diseases, including ALS and FTD, for a total number of 118 genes; it features over 8.7 Mb of content, including exons, introns, promoters and untranslated regions.

Targeted resequencing was performed using the TG NextSeq® 500/550 High Output Kit v2.1 (300 cycles) according to manufacturer’s protocol, using a NextSeq platform (Illumina, San Diego, CA, USA). Sequence reads mapping to the Human Genome version 19 (hg/19/GRCh37) and variant calling were performed with BaseSpace Onsite Sequence Hub. The reference coding of NEK1, NM_001199397.1, was used.

Single variants reported in the FASTQ and VCF output file were analyzed with Illumina Variant Studio V3.0 software (http://variantstudio.software.illumina.com/) and visualized via Integrative Genome Viewer (IGV) software (<http://www.broadinstitute.org/software/igv/>).

*Filters*

Variants both in ALS and control cohorts were filtered based on the following criteria: (i) coverage depth >10; (ii) intron and synonymous variants were excluded except for those located within or near splice sites; (iii) variants with global or European population minor allele frequencies (MAF) > 0.01 identified in the dbSNP150 database (www.ncbi.nlm.nih.gov/projects/SNP/) or in The Genome Aggregation Database (gnomAD; <https://gnomad.broadinstitute.org/>) were filter out, in agreement with the ACMG classification criteria ([Richards, Aziz et al. 2015](#_ENREF_13)), with the exception of variants reported as pathogenic or of uncertain significance ([Cady, Allred et al. 2015](#_ENREF_3); [Morgan, Shoai et al. 2015](#_ENREF_8); [Nishiyama, Niihori et al. 2017](#_ENREF_11); [Naruse, Ishiura et al. 2019](#_ENREF_9); [Scarlino, Domi et al. 2020](#_ENREF_14)). Sorting Intolerant from Tolerant (SIFT; sift.jcvi.org), Polymorphism Phenotyping (PolyPhen-2; genetics.bwh.harvard.edu/pph2/) and Mutation Taster (www.mutationtaster.org/) prediction tools were used to evaluate the functional effect of candidate missense variants. MaxEntScan (<http://genes.mit.edu/burgelab/maxent/Xmaxentscan_scoreseq.html>), Splice Site Prediction by Neural Network Site (http://www.fruitfly.org/seq_tools/splice.html, Berkley, CA, USA) and Gene Splicer (http://www.cbcb.umd.edu/software/GeneSplicer/gene_spl.shtml) were used to evaluate the potential effects on gene splicing. The ALSoD database (<https://alsod.ac.uk/>) was used to filter the ALS-related genes within the 118 panel genes in order to evaluate oligogenicity of *NEK1* carriers. The 33 ALS-related genes are the following: ALS2, ANG, APOE, C21orf2, CHCHD10, CHMP2B, DCTN1, EPHA4, FIG4, FUS, GRN, HNRNPA1, HNRNPA2B1, LMNB1, MAPT, NEFH, NEK1, OPTN, PARK7, PFN1, PSEN1, SARM1, SETX, SOD1, SPAST, SQSTM1, TARDBP, TBK1, TUBA4A, UBQLN2, UNC13A, VAPB, VCP.

*Sanger Sequencing Validation*

All the identified *NEK1* variants found in patients and patient’s relatives were confirmed by Sanger sequencing, using the Big Dye TerminatorV1.1 Cycle Sequencing Kit (Applied Biosystems). PCR and sequencing reactions were purified using AMPure (Agencourt-Beckmann Coulter, Inc., Brea, CA, USA) and Big Dye X-Terminator Kit (Applied Biosystems Foster City, CA, USA), respectively, according to protocols developed in the laboratory.([Pozzi, Valenza et al. 2017](#_ENREF_12)) Dye terminator reaction sequences were run on an ABI 3730 Genetic Analyzer (Applied Biosystems Foster City, CA, USA). Called Sequences were aligned with the reference sequence (hg/19/GRCh37) using Sequencer 5.0 Software (Gene Codes). See table S1 for Primers list and figures S2, S3 for Sanger validation electropherograms. Reporting was performed according to the STREGA statement ([Little, Higgins et al. 2009](#_ENREF_7)).

**Figure S1: Pedigree of four families of *NEK1* carriers.**

I:1

*NEK1*: NA

*C9*: NA

II:1

*NEK1*: m/+

*C9*: +/+

I:2

*NEK1*: m/+

*C9*: +/+

II:2

*NEK1*: m/+

*C9*: m/+

Family #2

*NEK1*: p.C113R

*C9Orf72* Expansion

I:1

*NEK1*: NA

II:1

*NEK1*: m/+

I:2

*NEK1*: NA

Family #1

*NEK1*: c.3374+1G>A

II:2

*NEK1*: m/+

I:1

*NEK1*: NA

*TARDBP*: NA

II:1

*NEK1*: +/+

*TARDBP*: m/+

I:2

*NEK1*: NA

*TARDBP*: NA

II:2

*NEK1*: m/+

*TARDBP*: m/+

Family #4

*NEK1*: p.R261H

*TARDBP*: p.G368S

I:1

*NEK1*: NA

*C9*: NA

I:2

*NEK1*: NA

*C9*: NA

Family #3

*NEK1*: p.N732S

*C9Orf72* Expansion

II:2

*NEK1*: +/+

*C9*: m/+

II:1

*NEK1*: +/+

*C9*: m/+

II:3

*NEK1*: m/+

*C9*: m/+

**Key:** square: male individual; circle: female individual. Black symbols: patients with ALS. NA: no DNA available for testing; m/+: heterozygous mutation; +/+: wild type gene.

**Figure S2: Sanger validation of NEK1 variants found in our NGS analysis.**

- ALS_41: p.Leu507GlufsTer8 (delTT)


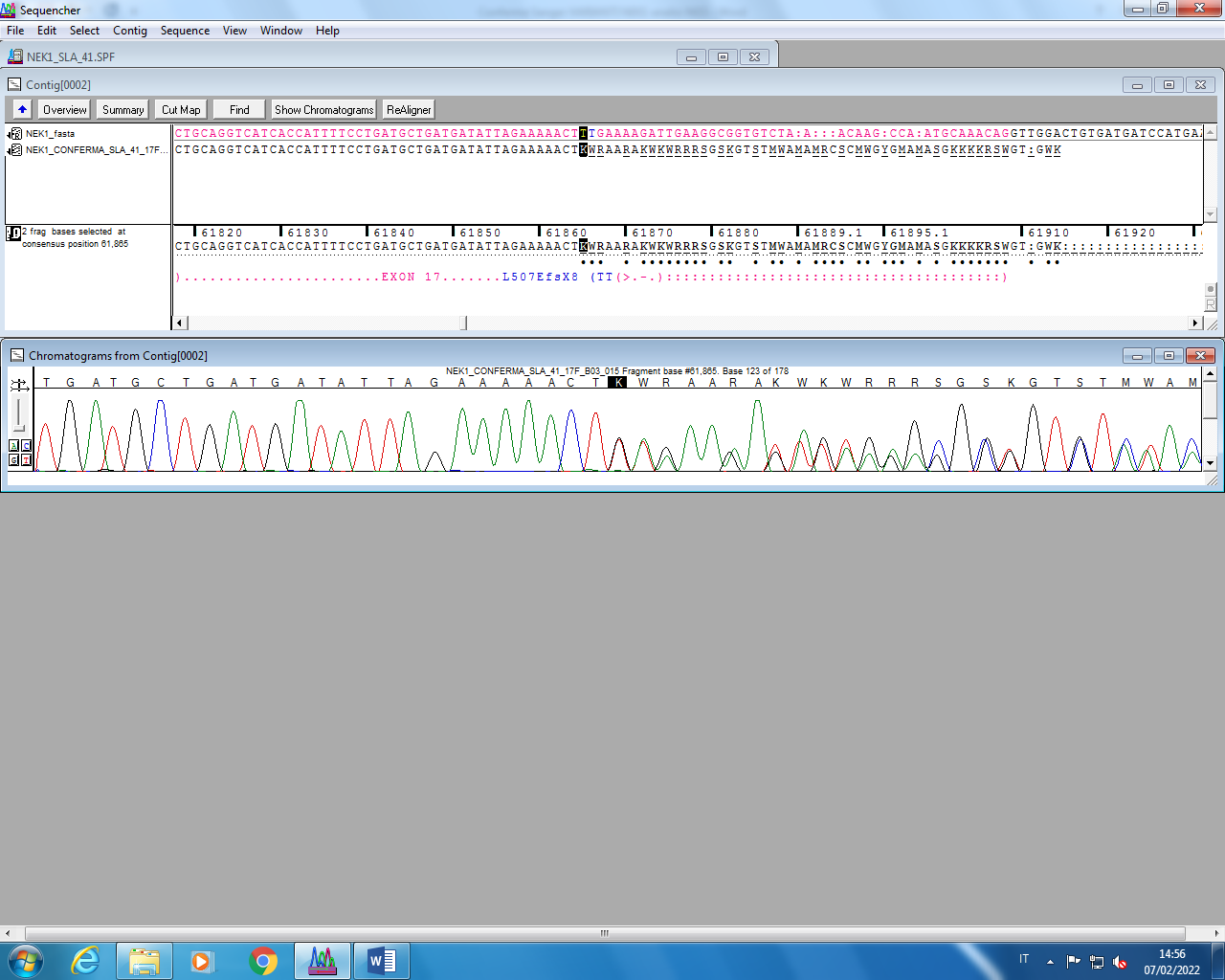


- ALS_798: c.3222+1G>A + p.Asp1093Val (A>T)


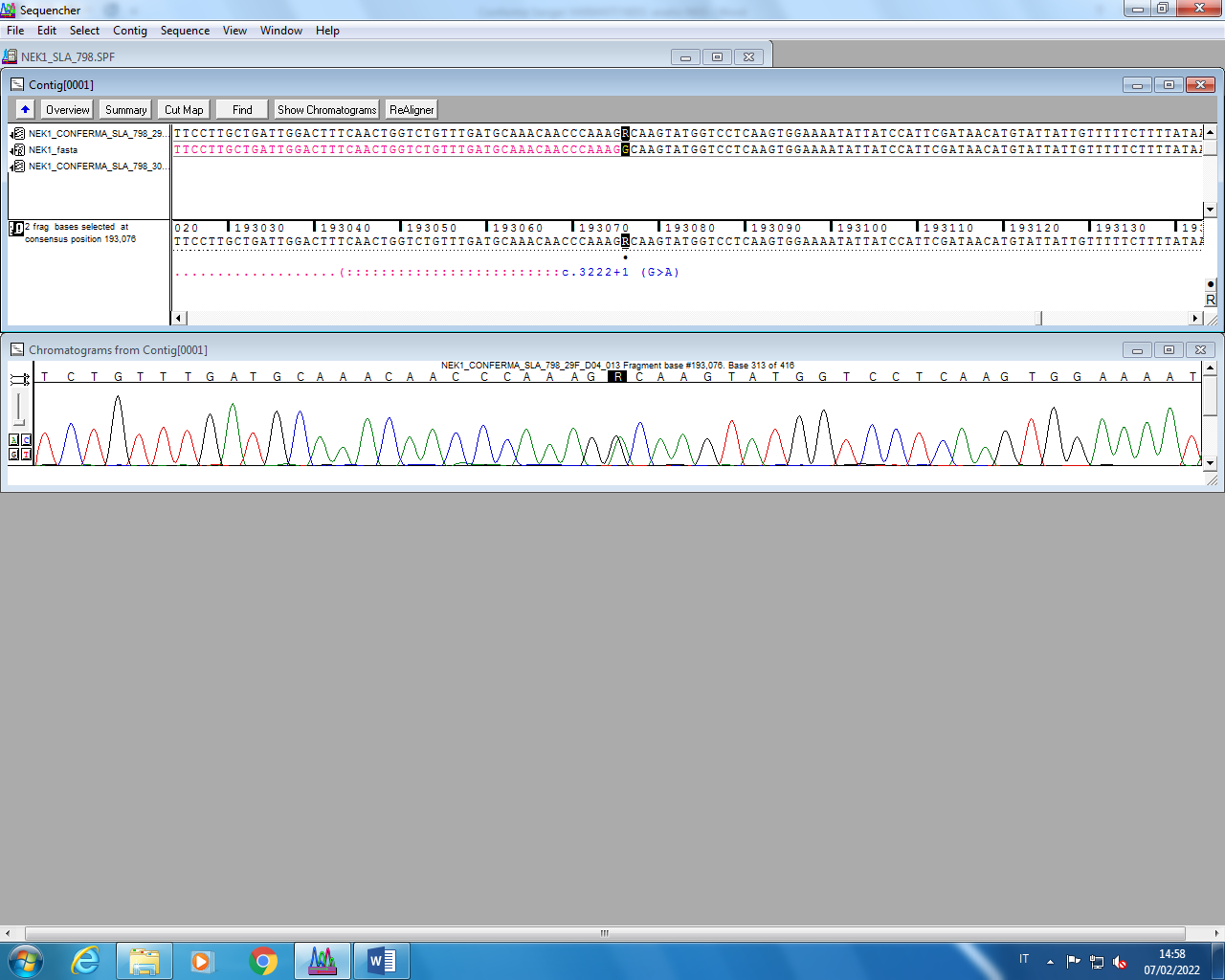


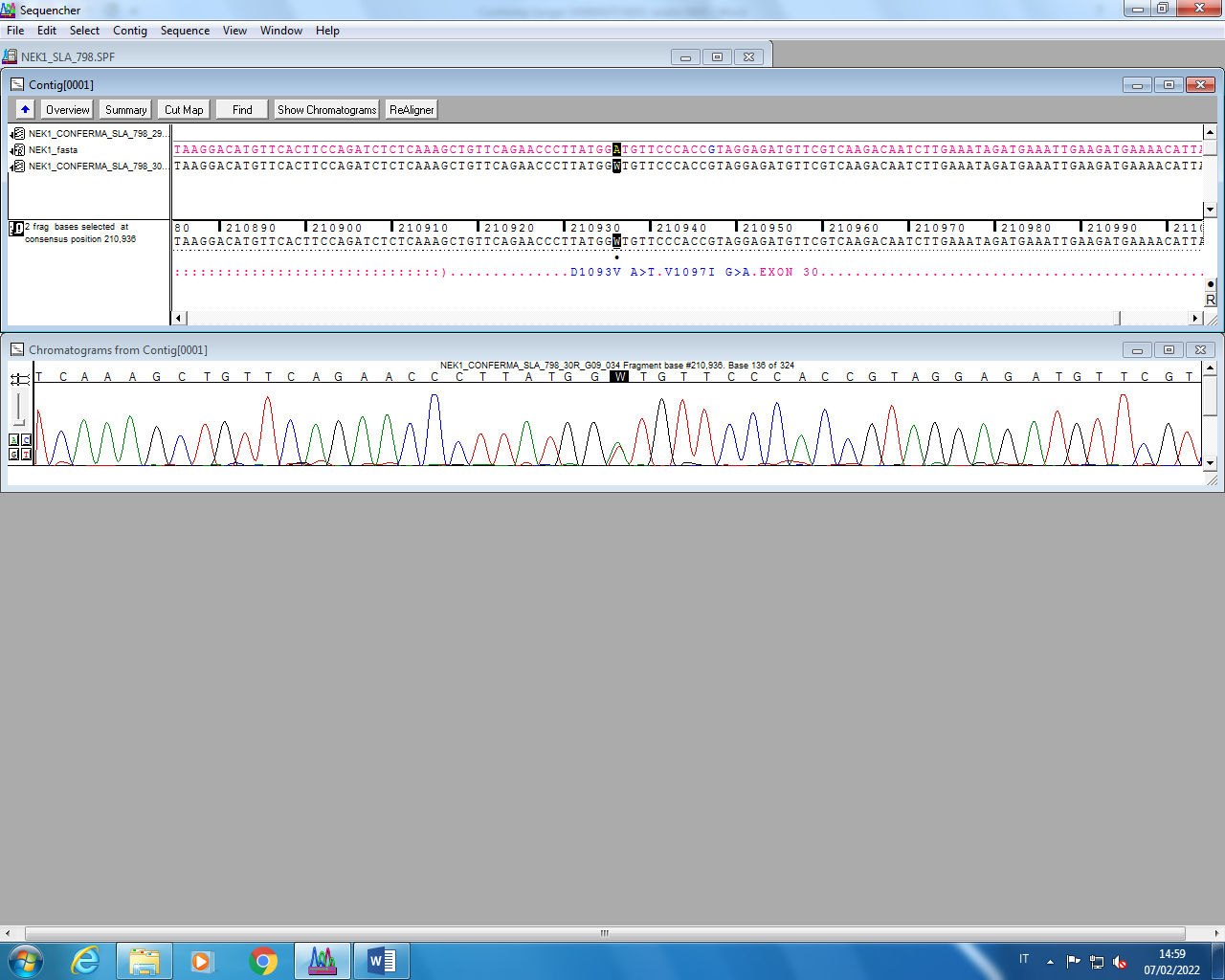


- ALS_716: c.3374+1G>A


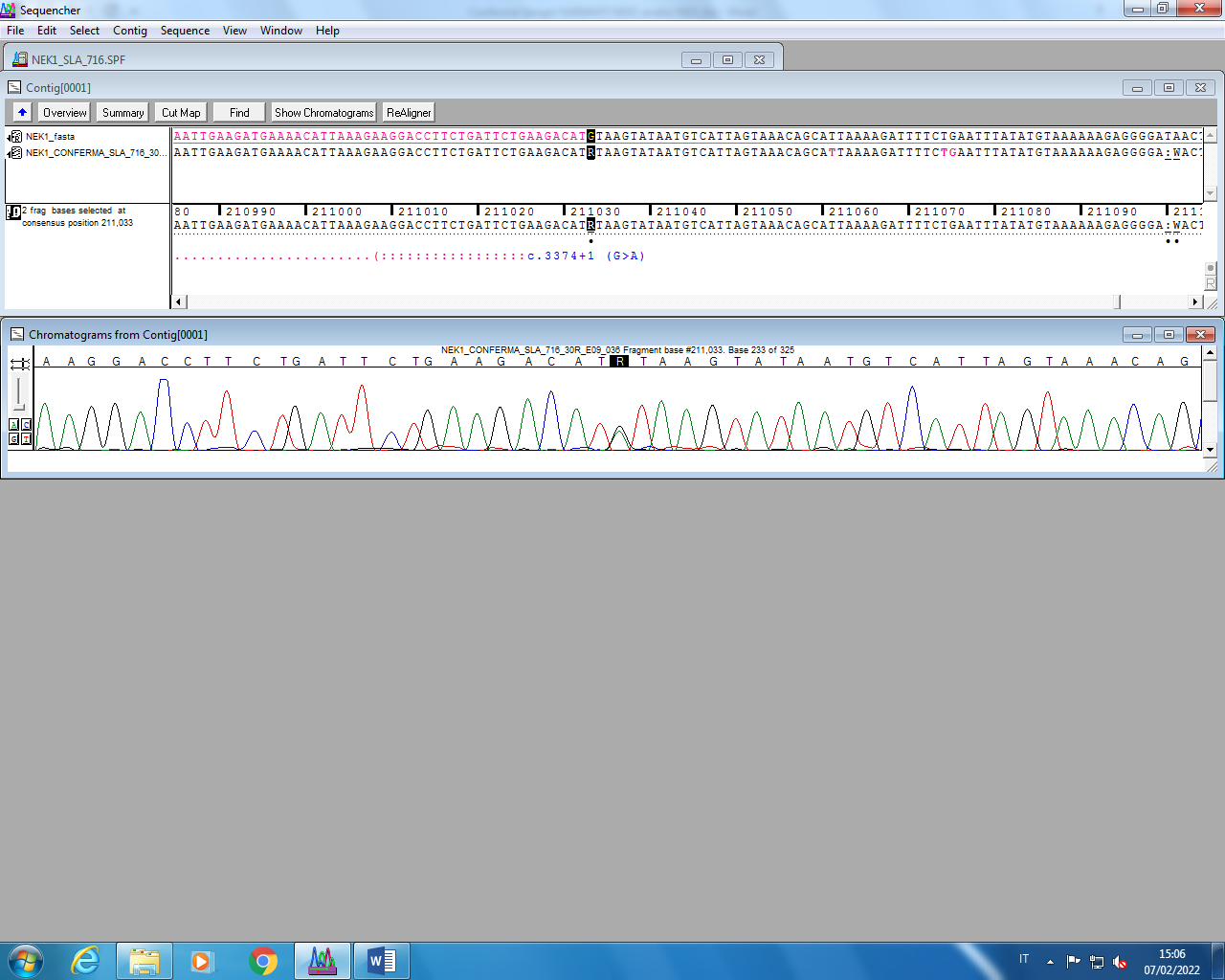


- ALS_293: p.Ile1254TyrfsTer5 (delAATAGTTCAAA)


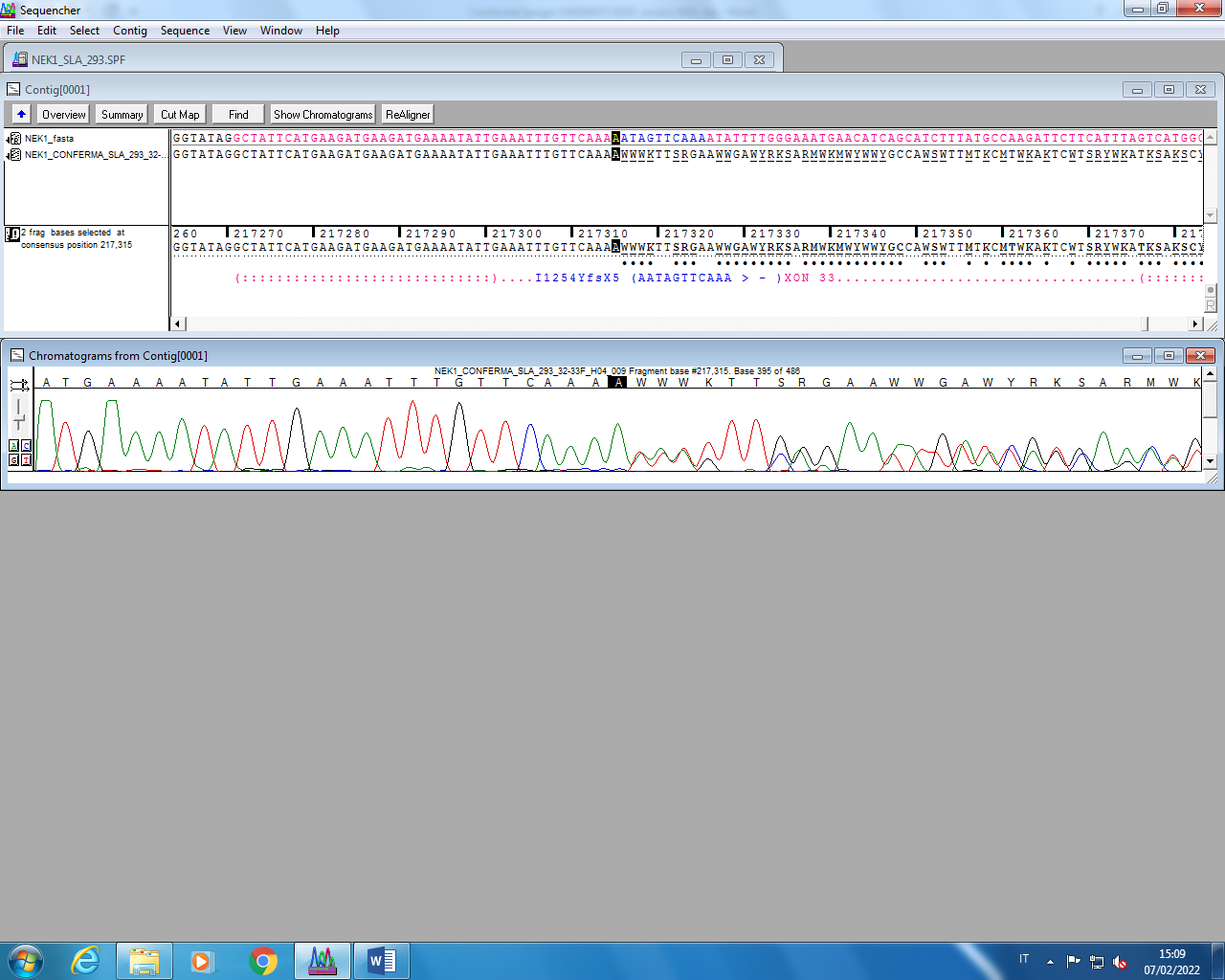


- ALS_315: p.Cys113Arg (T>C)


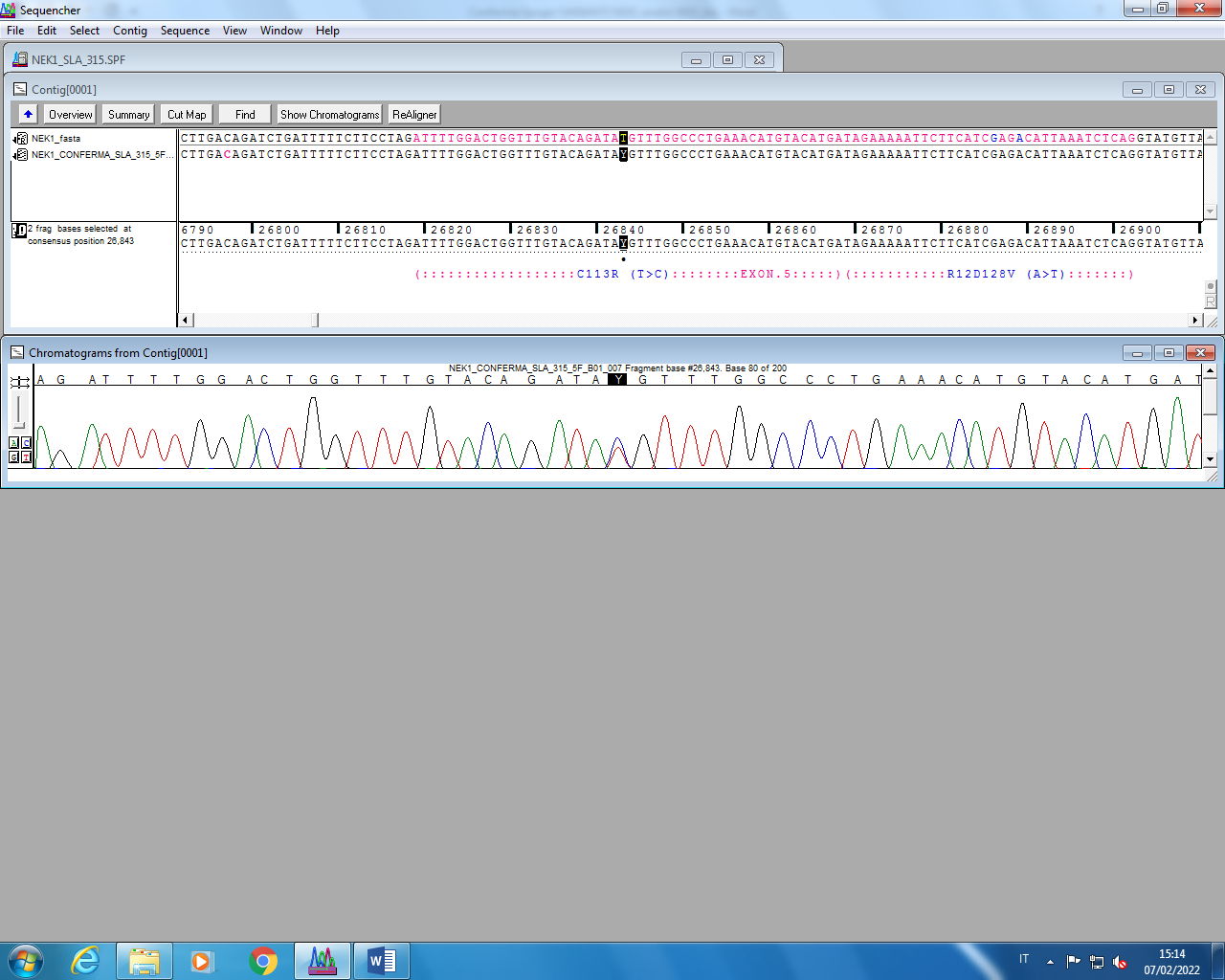


- ALS_119: p.Arg127Gln(G>A)


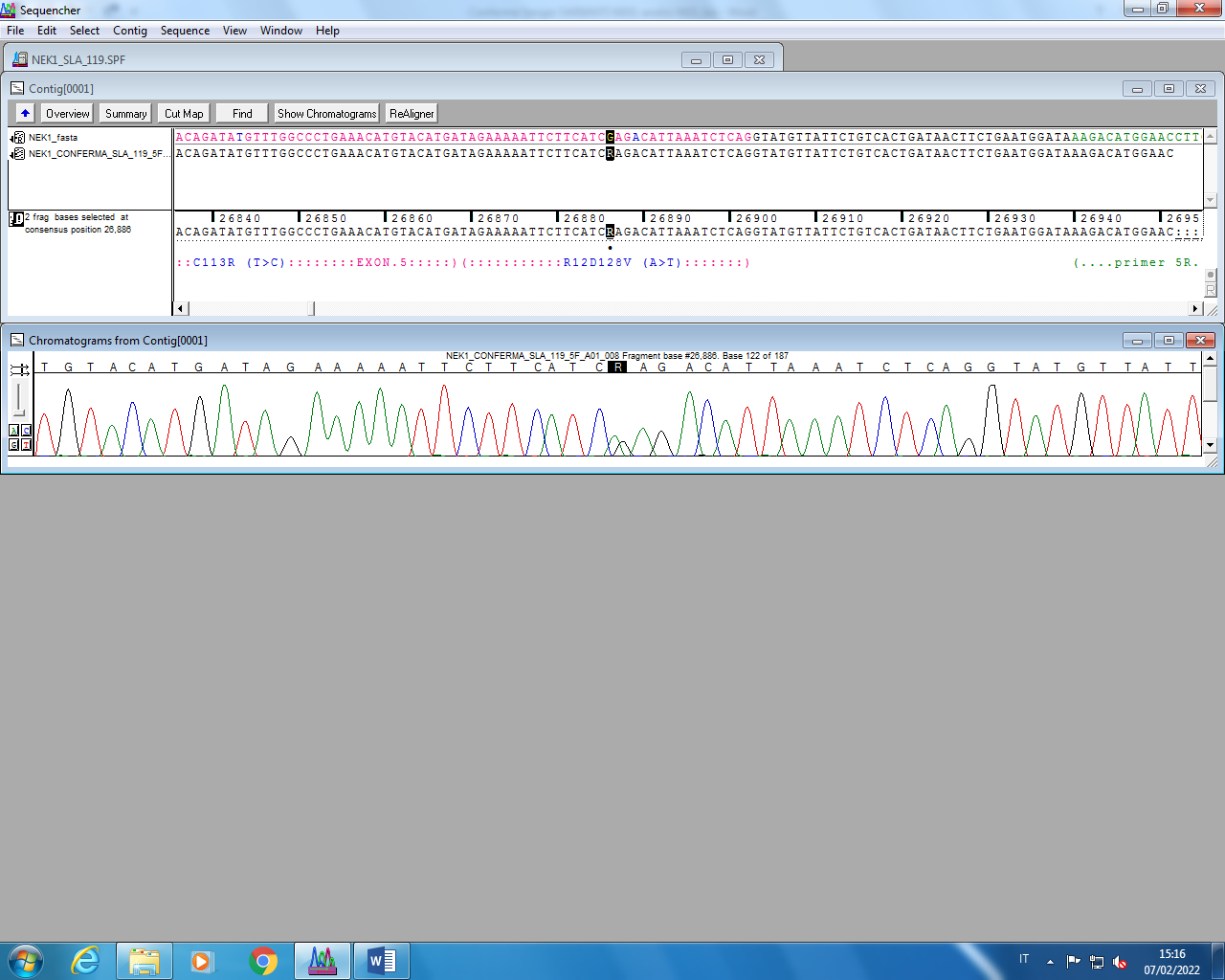


- ALS_695: p.Asp128Val (A>T) + p.Arg261His (G>A)
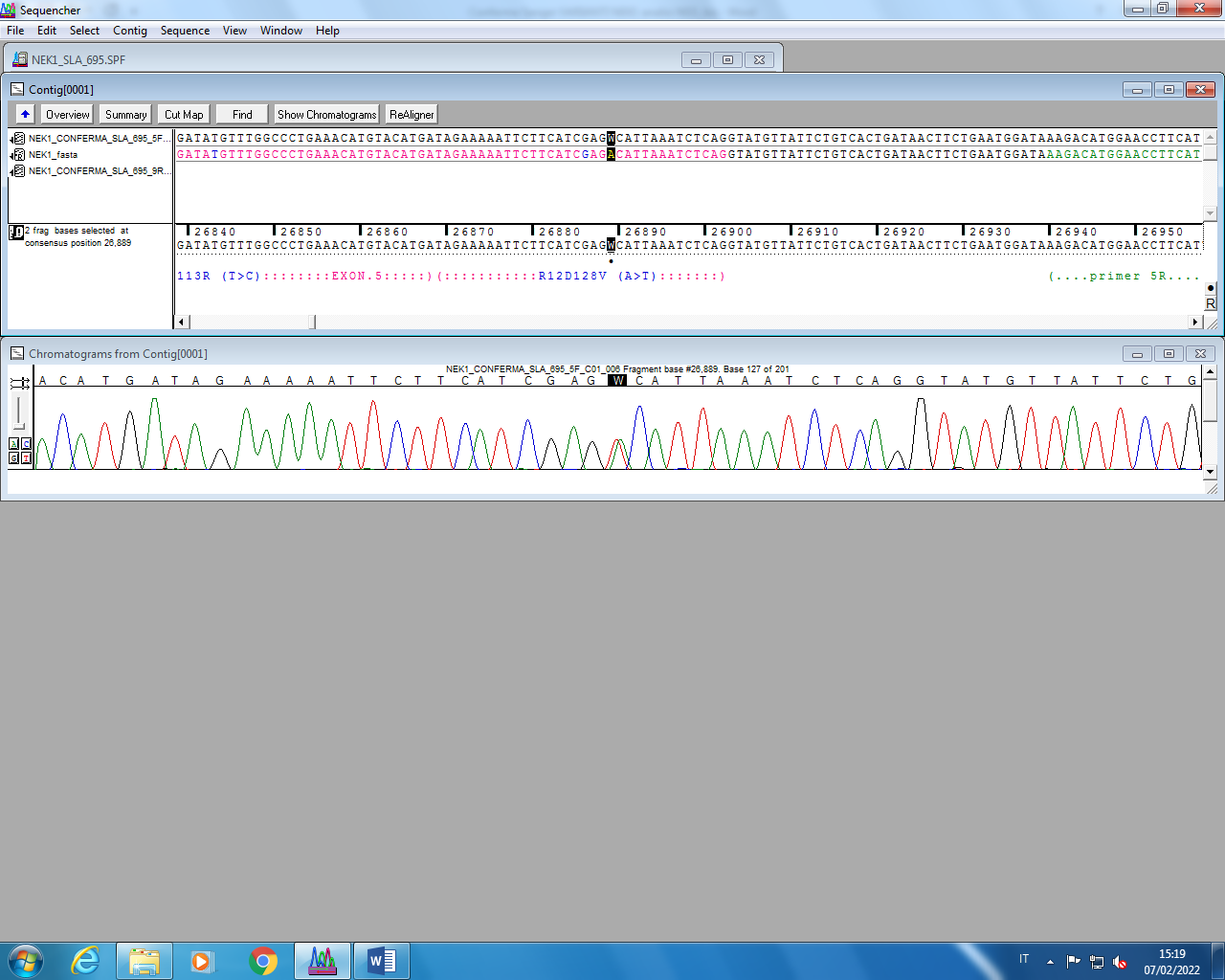

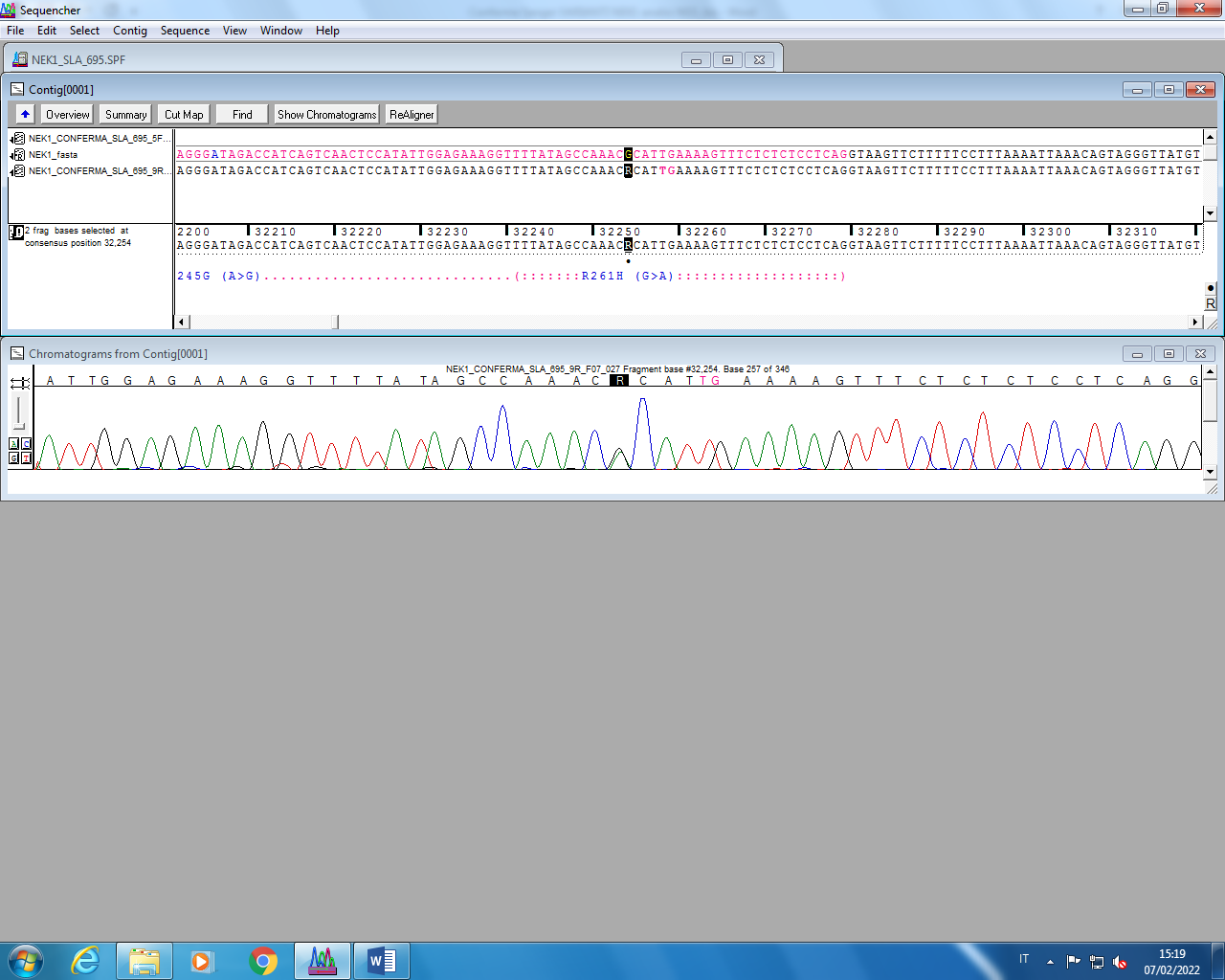

- ALS_367: p.Val157Leu (G>C)


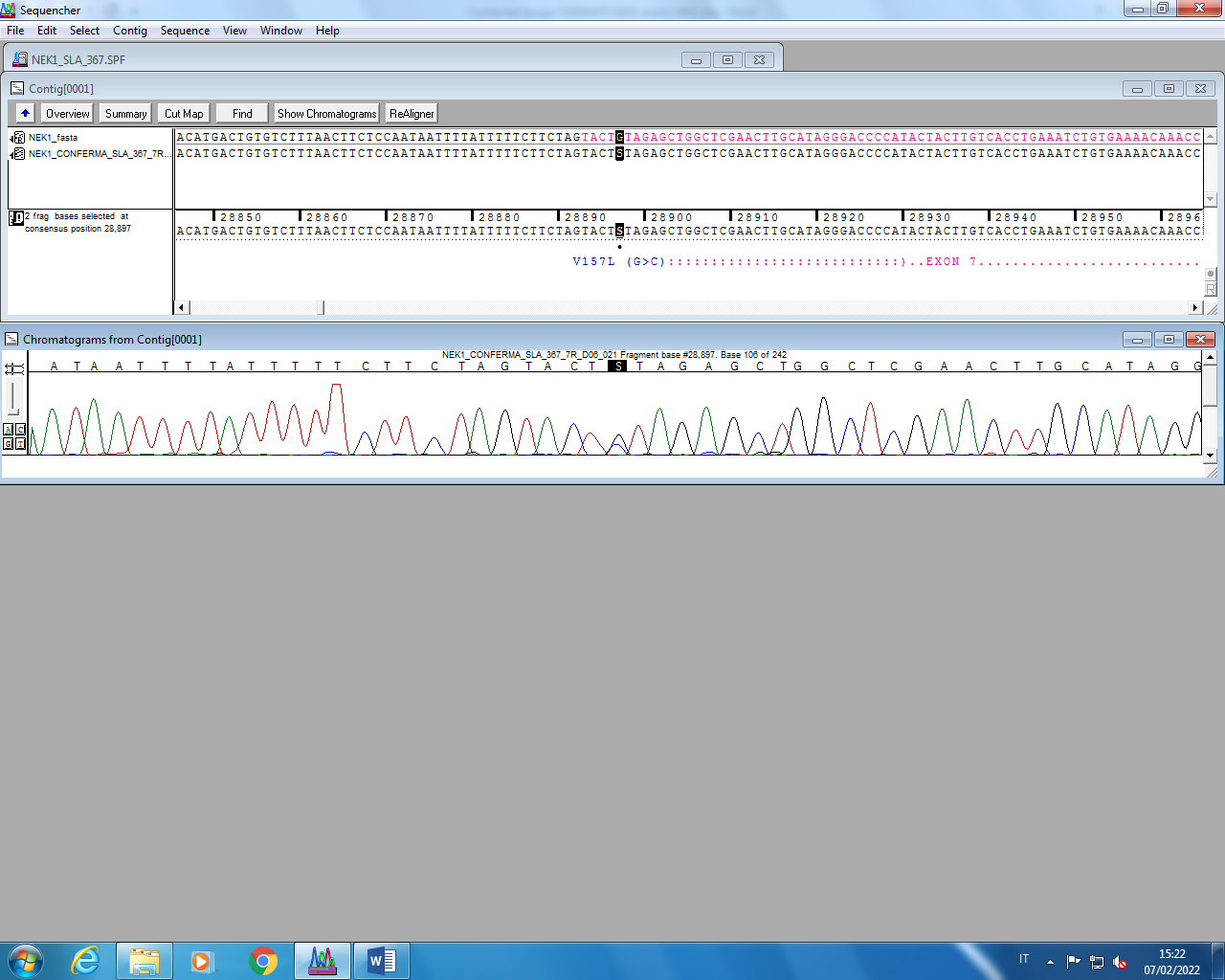


- ALS_549: p.Asp245Gly (A>G)


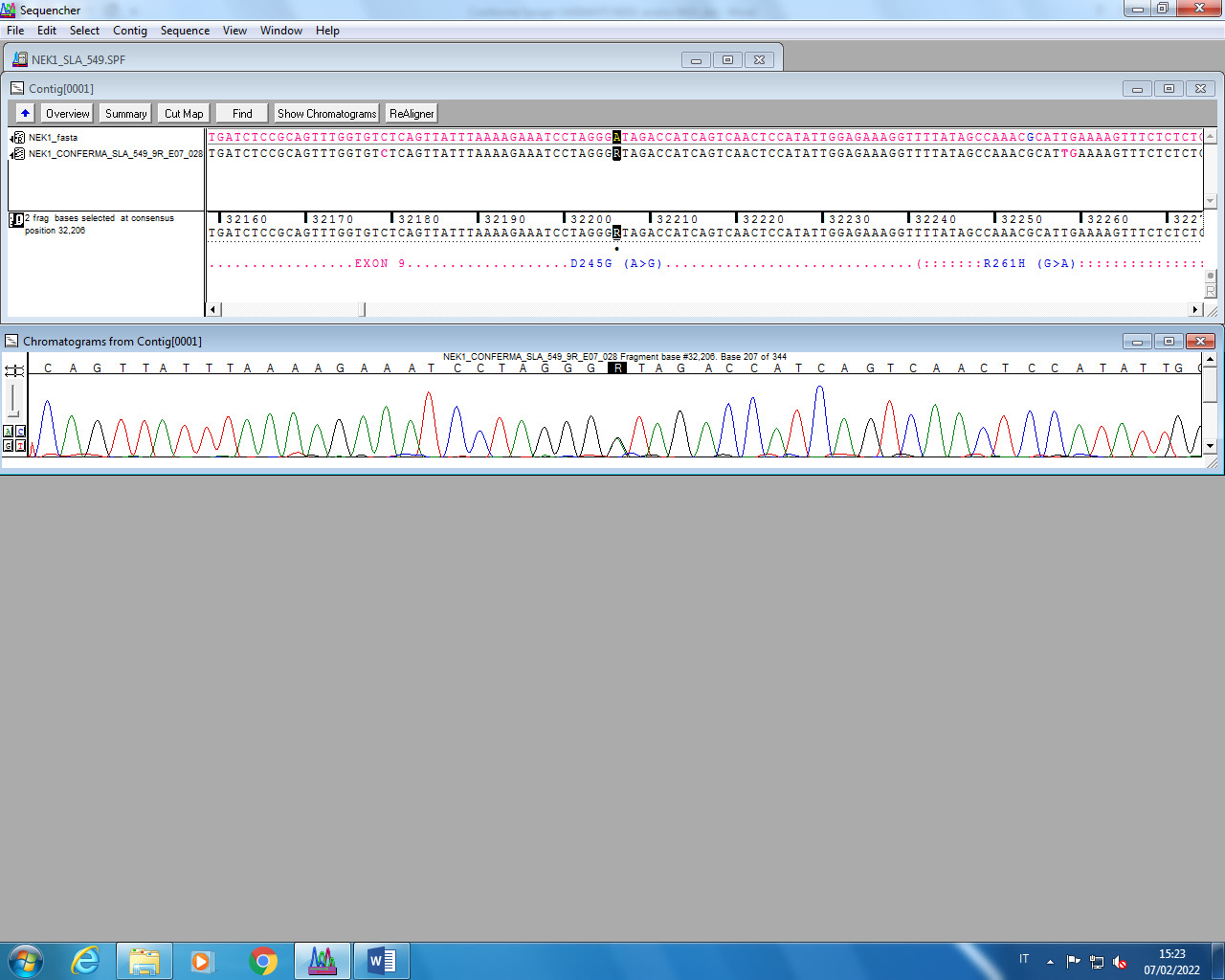


- ALS_152: p.Arg261His (G>A)


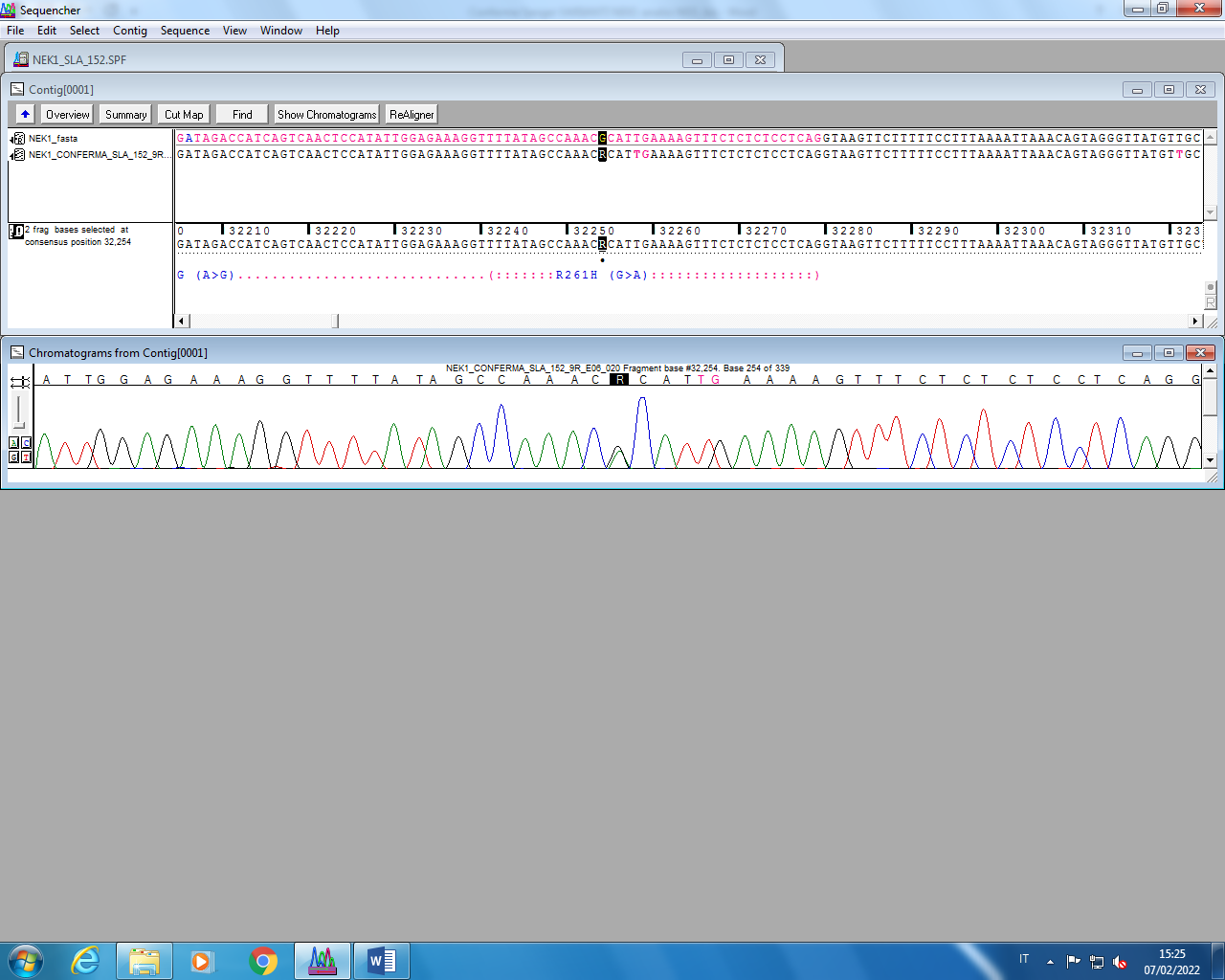


- ALS_155: p.Arg261His (G>A)


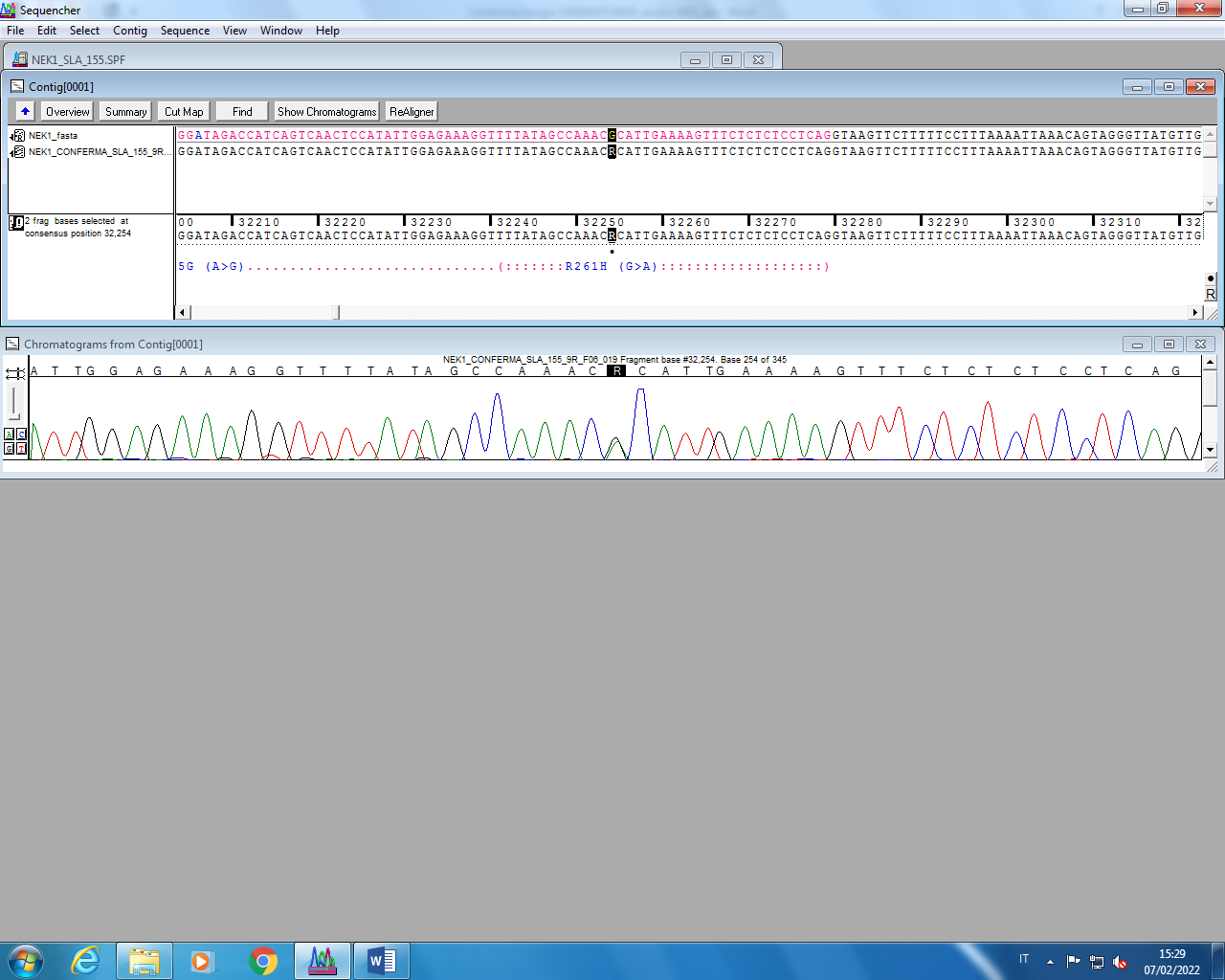


- ALS_200: p.Arg261His (G>A)


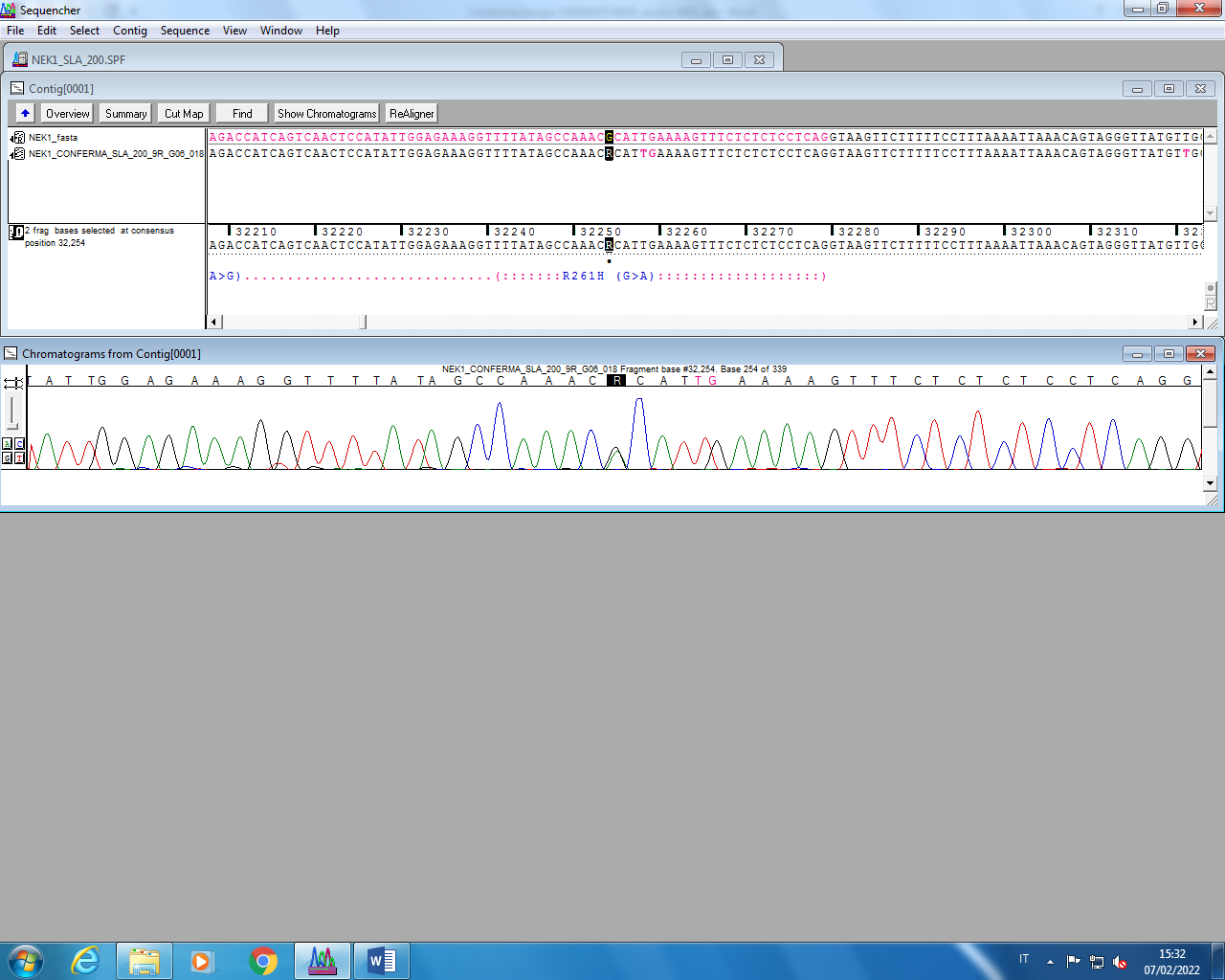


- ALS_209: p.Arg261His (G>A)


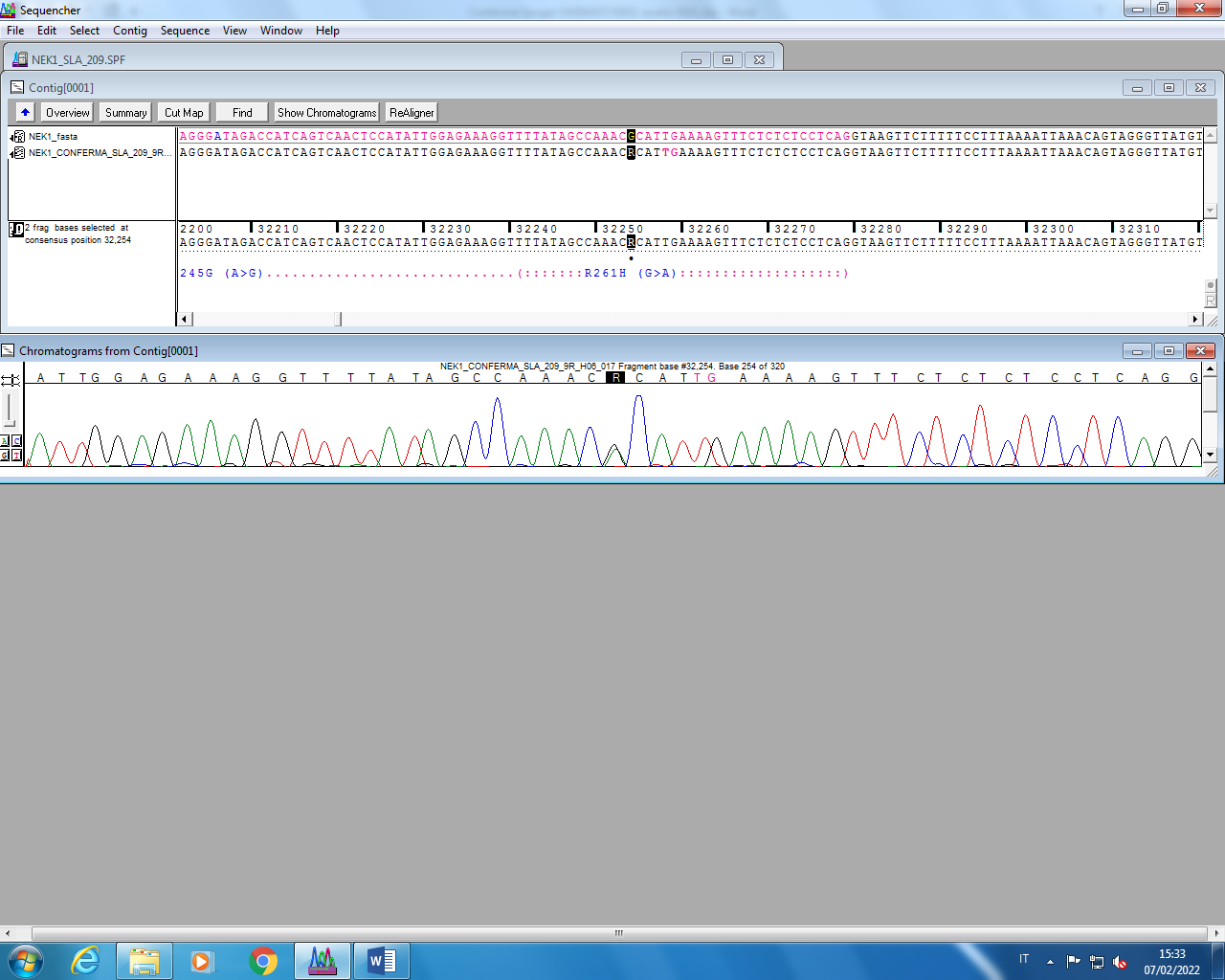


- ALS_444: p.Arg261His (G>A)


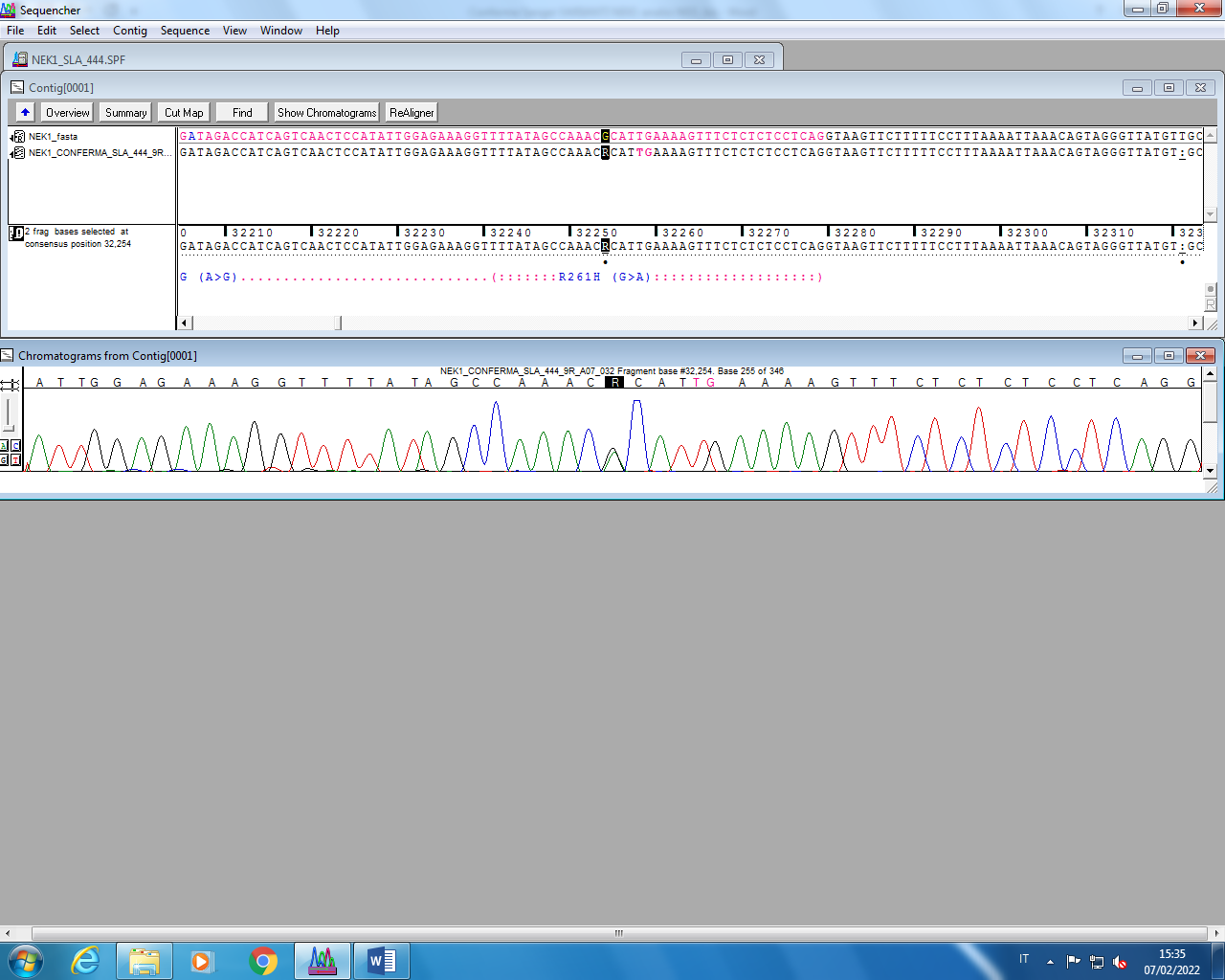


- ALS_464: p.Arg261His (G>A)


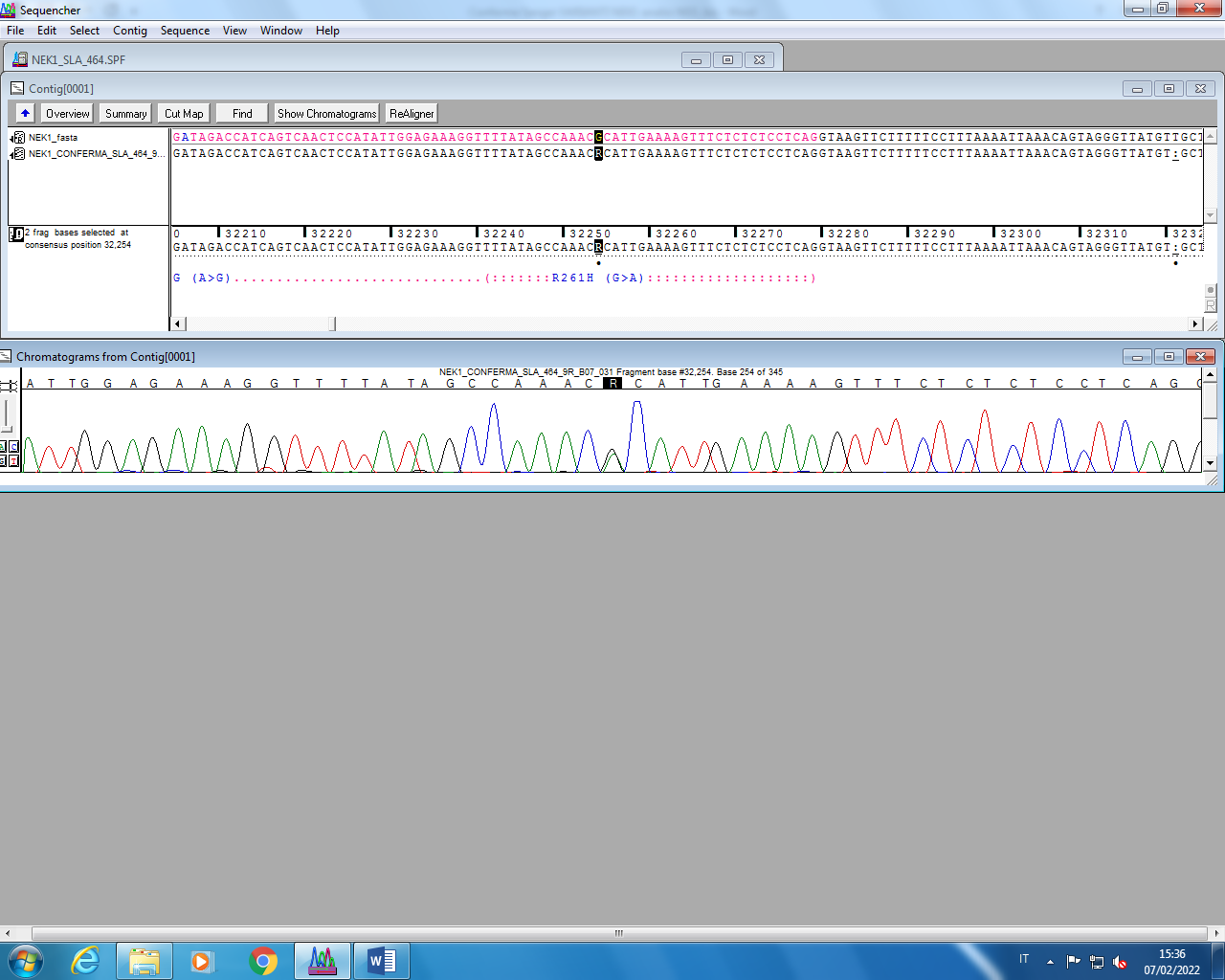


- ALS_480: p.Arg261His (G>A)


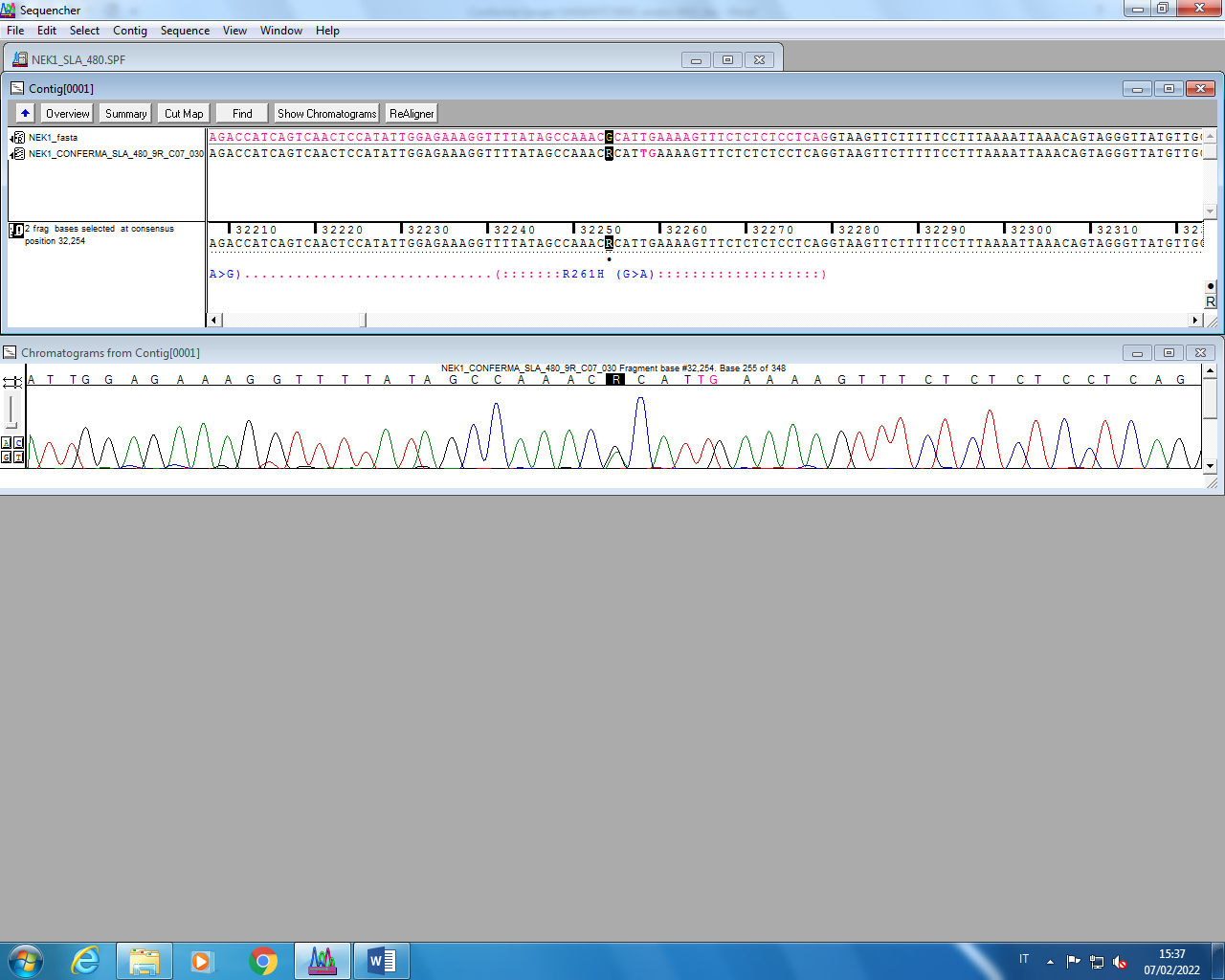


- ALS_512: p.Arg261His (G>A)


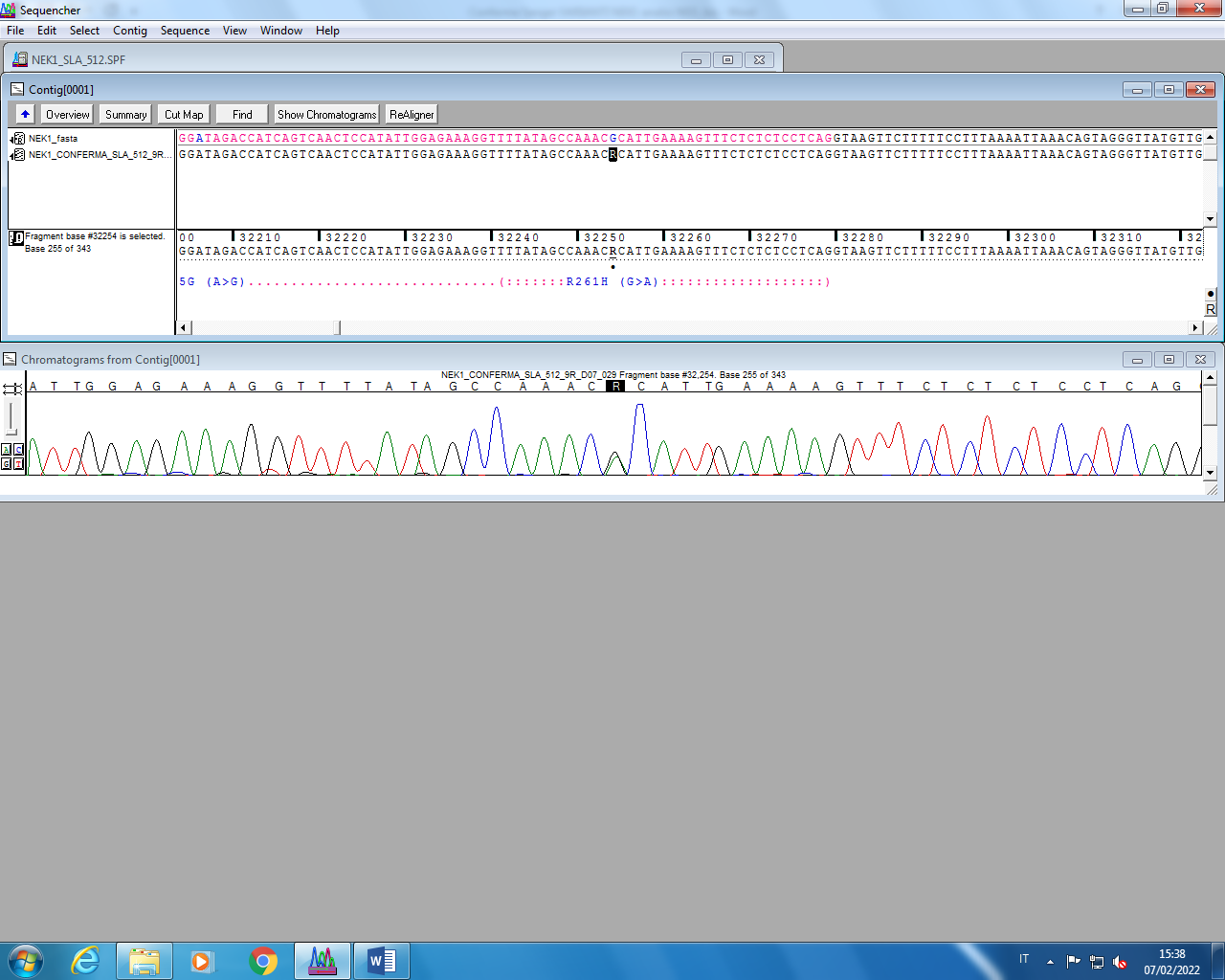


- ALS_839: p.Arg261His (G>A)


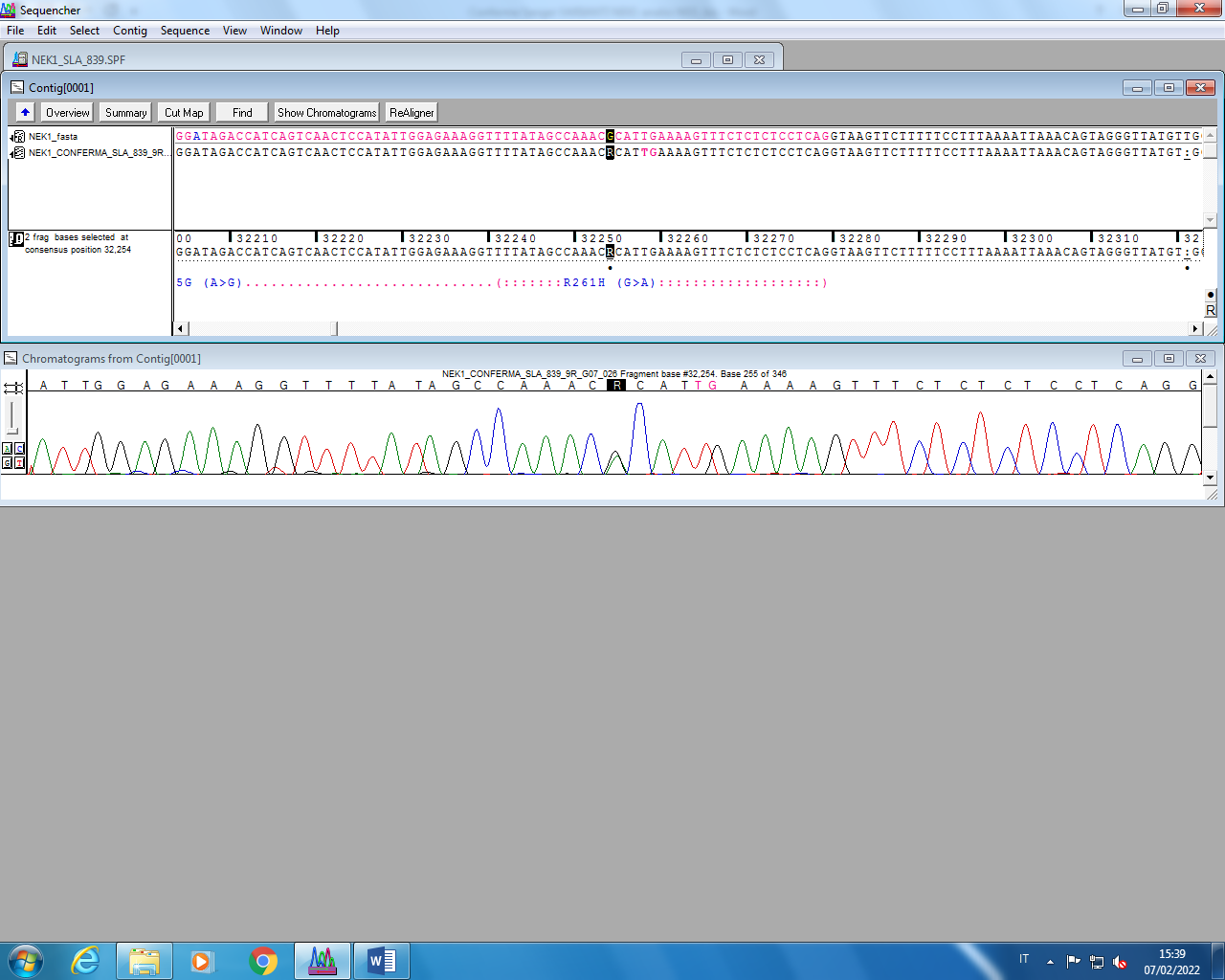


- ALS_855: p.Arg261His (G>A)


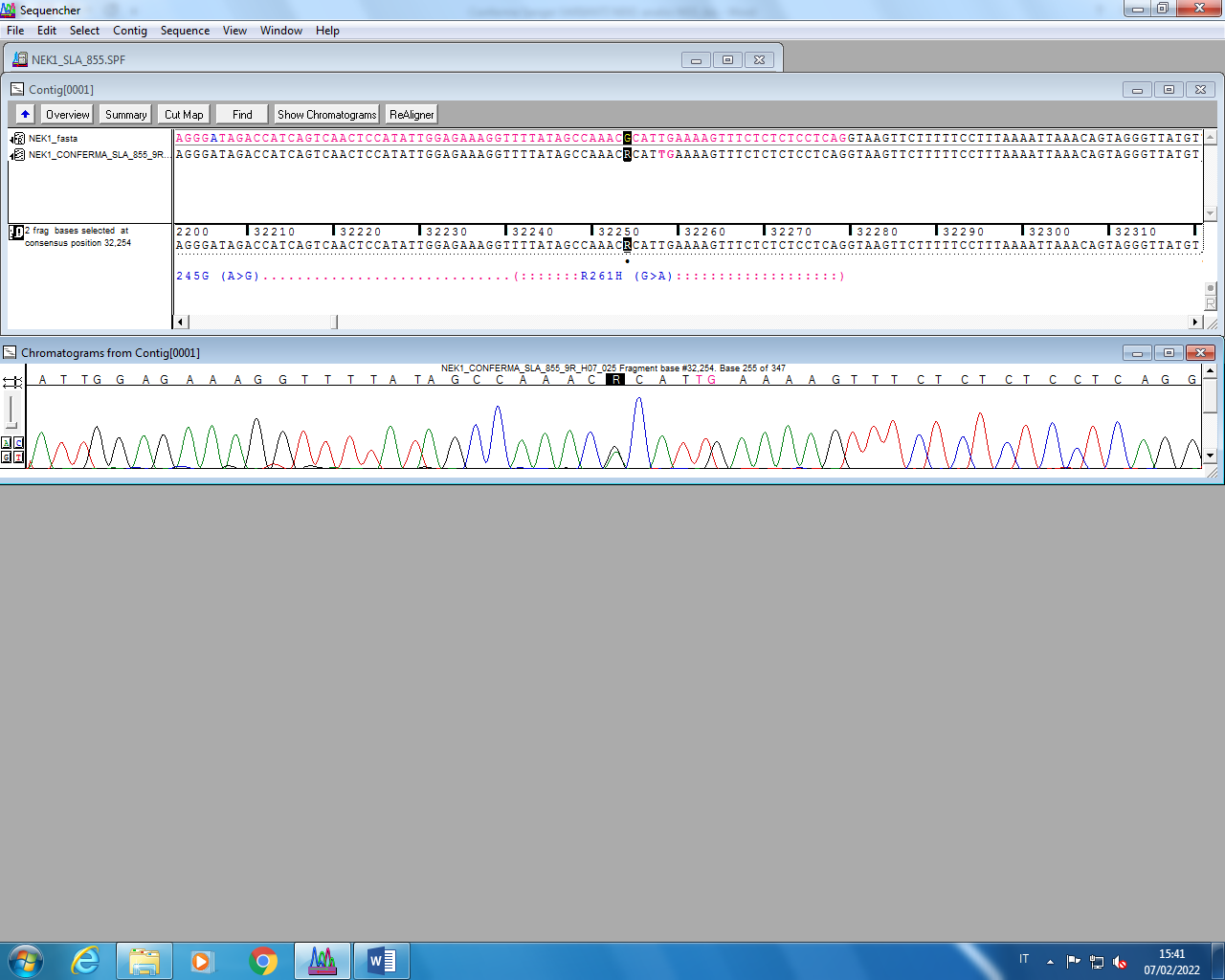


- ALS_883: p.Arg261His (G>A)


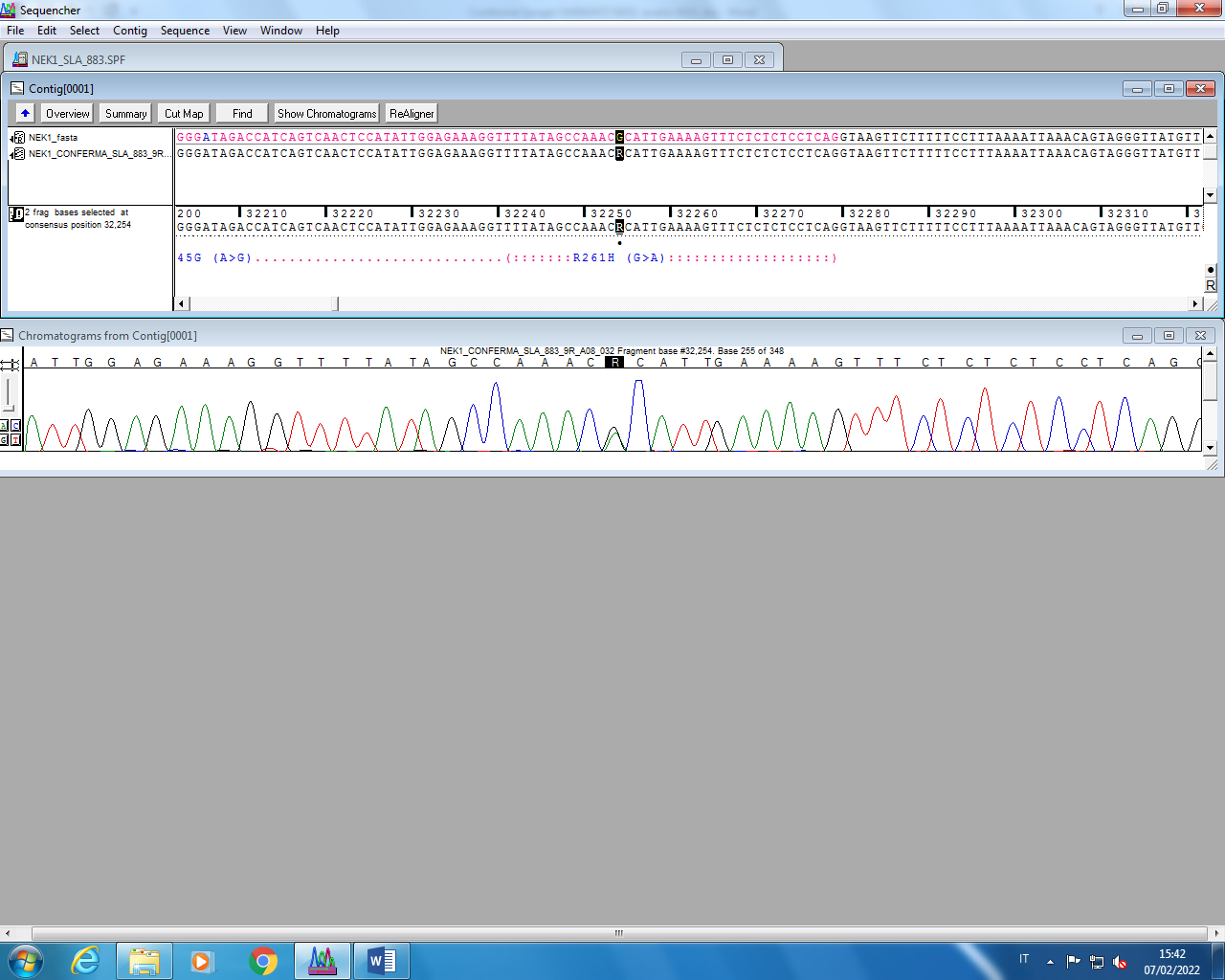


- ALS_974: p.Arg261His (G>A)


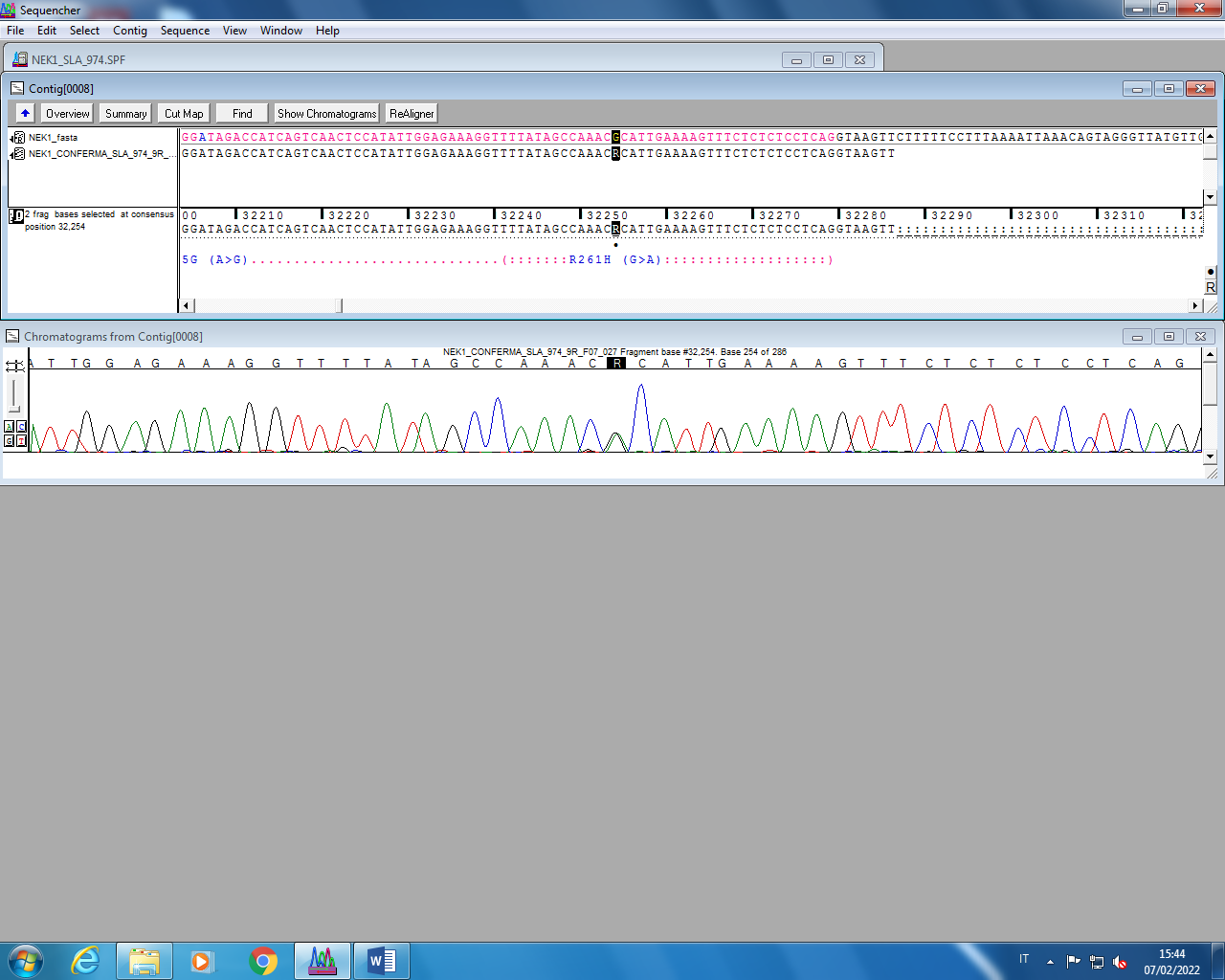


- ALS_742: p.Lys648Glu (A>G)


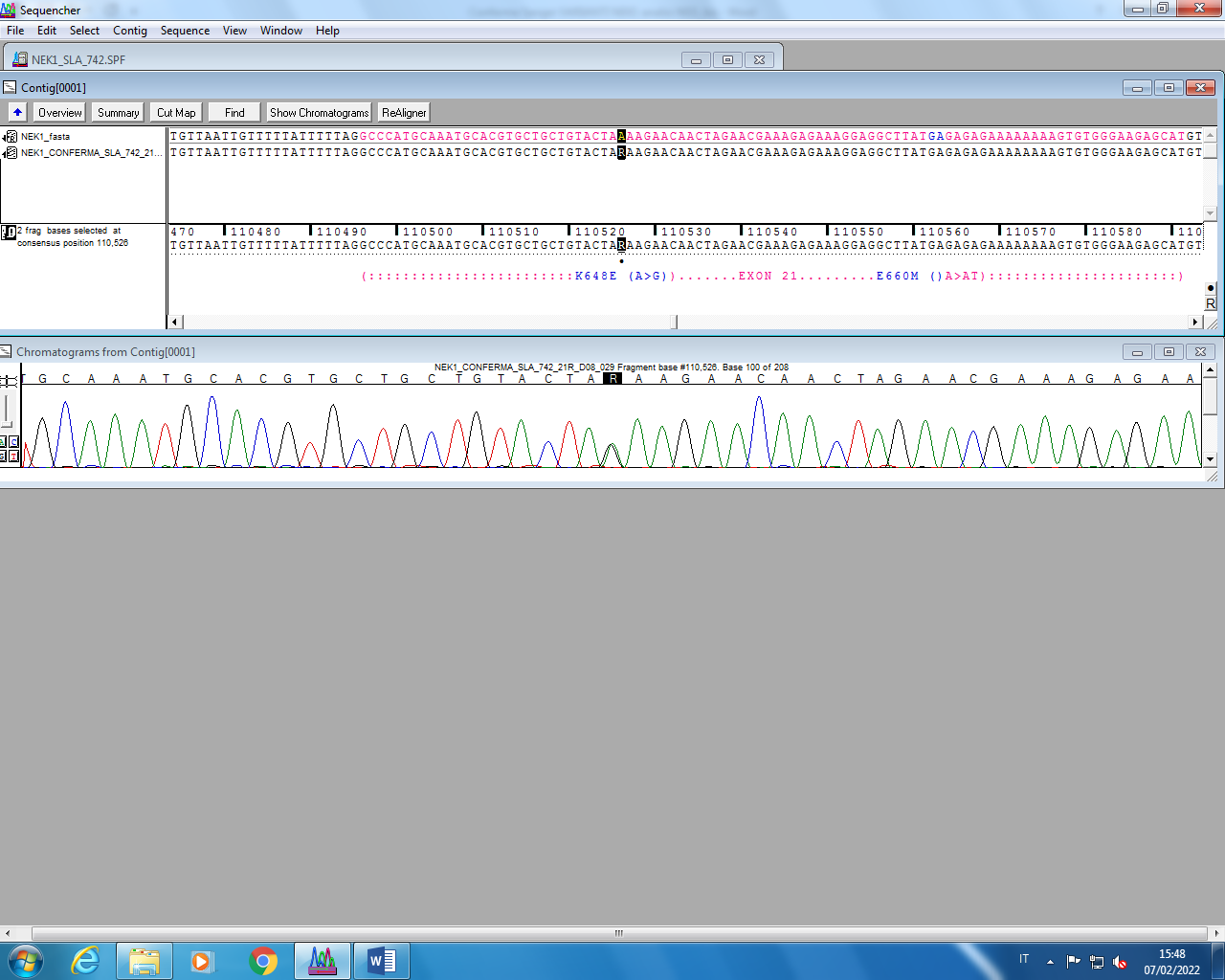


- ALS_101: p.Glu660Met (GA>AT)


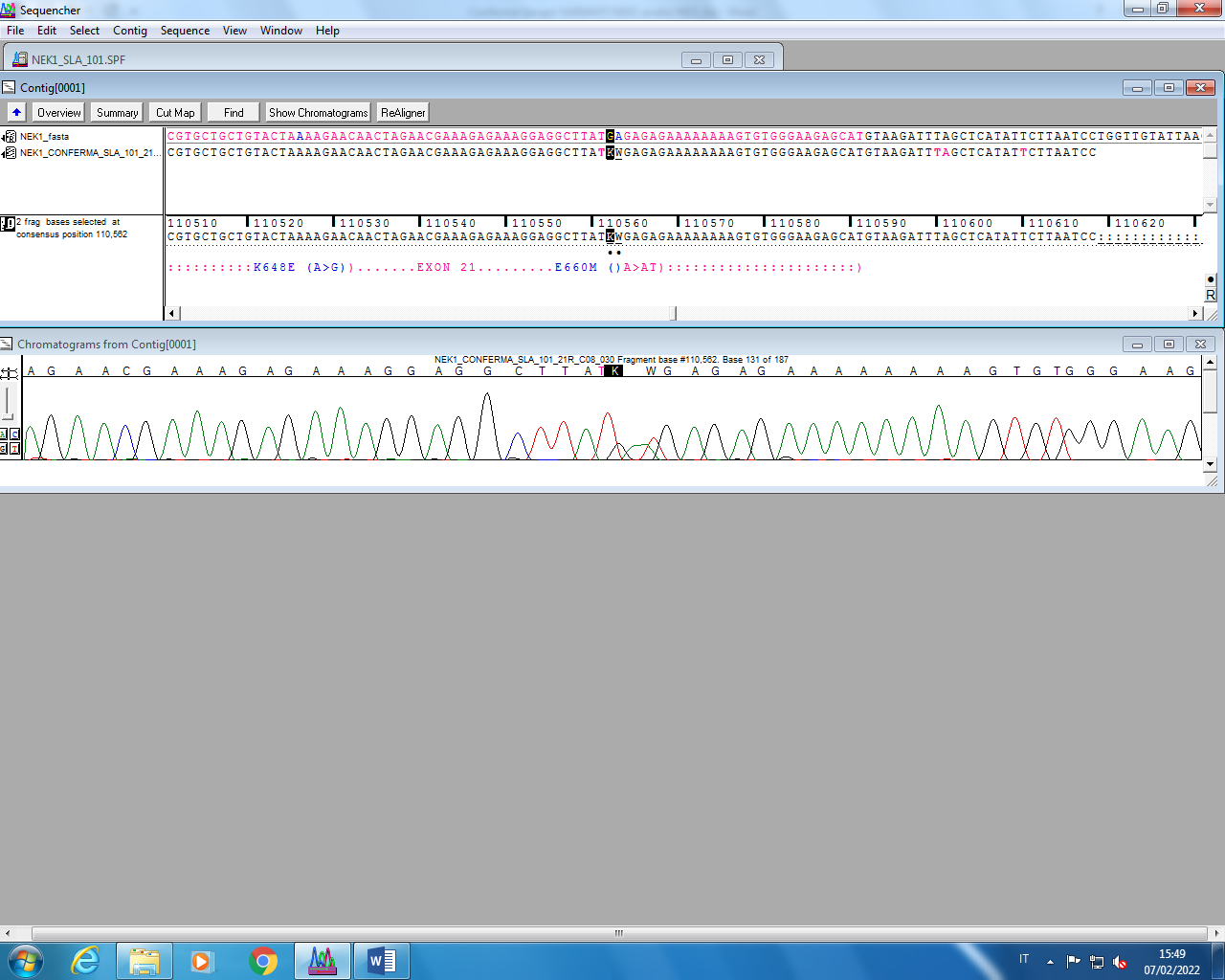


- ALS_190: p.Asn732Ser (A>G)


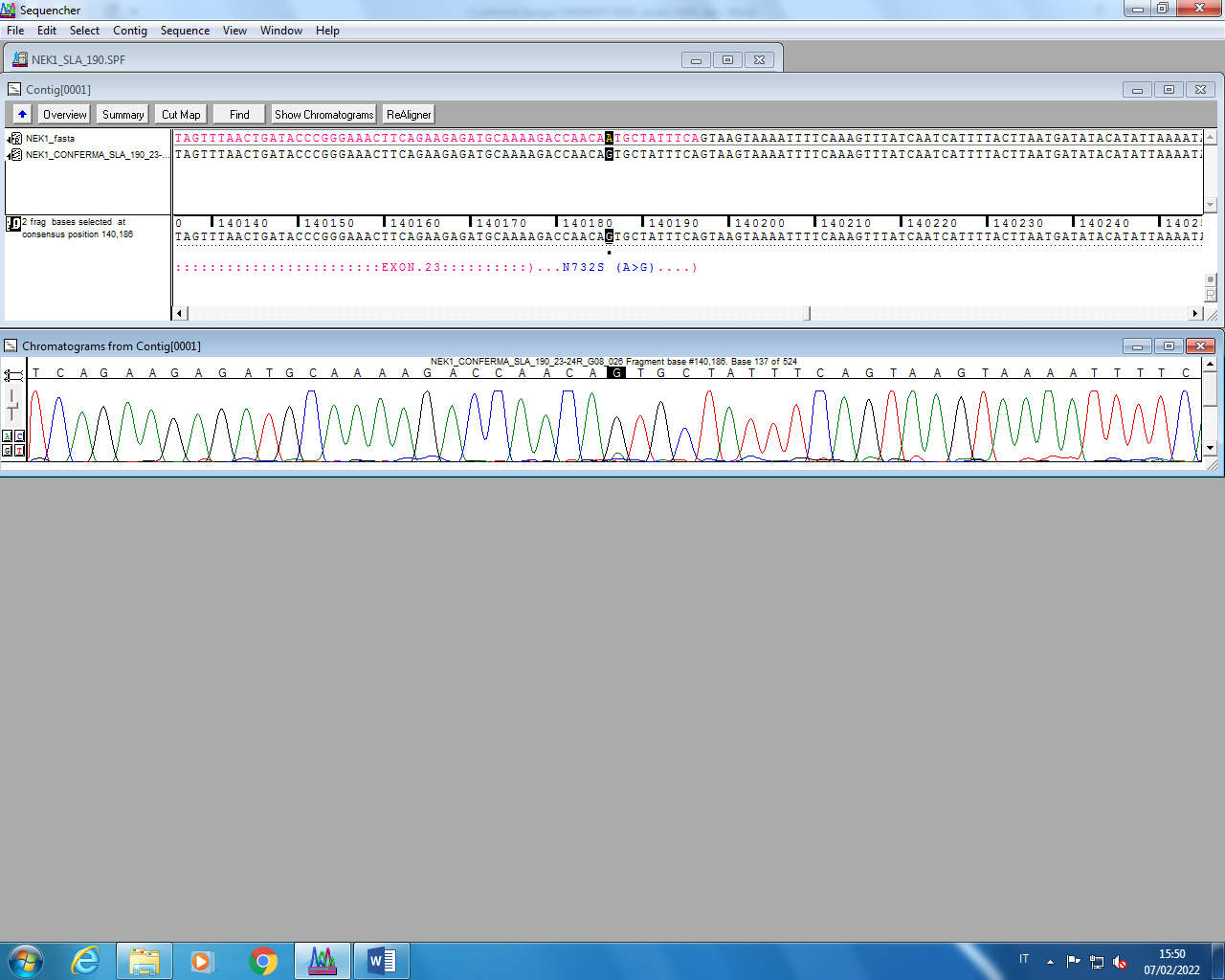


- ALS_320: p.Asn745Lys (T>G)


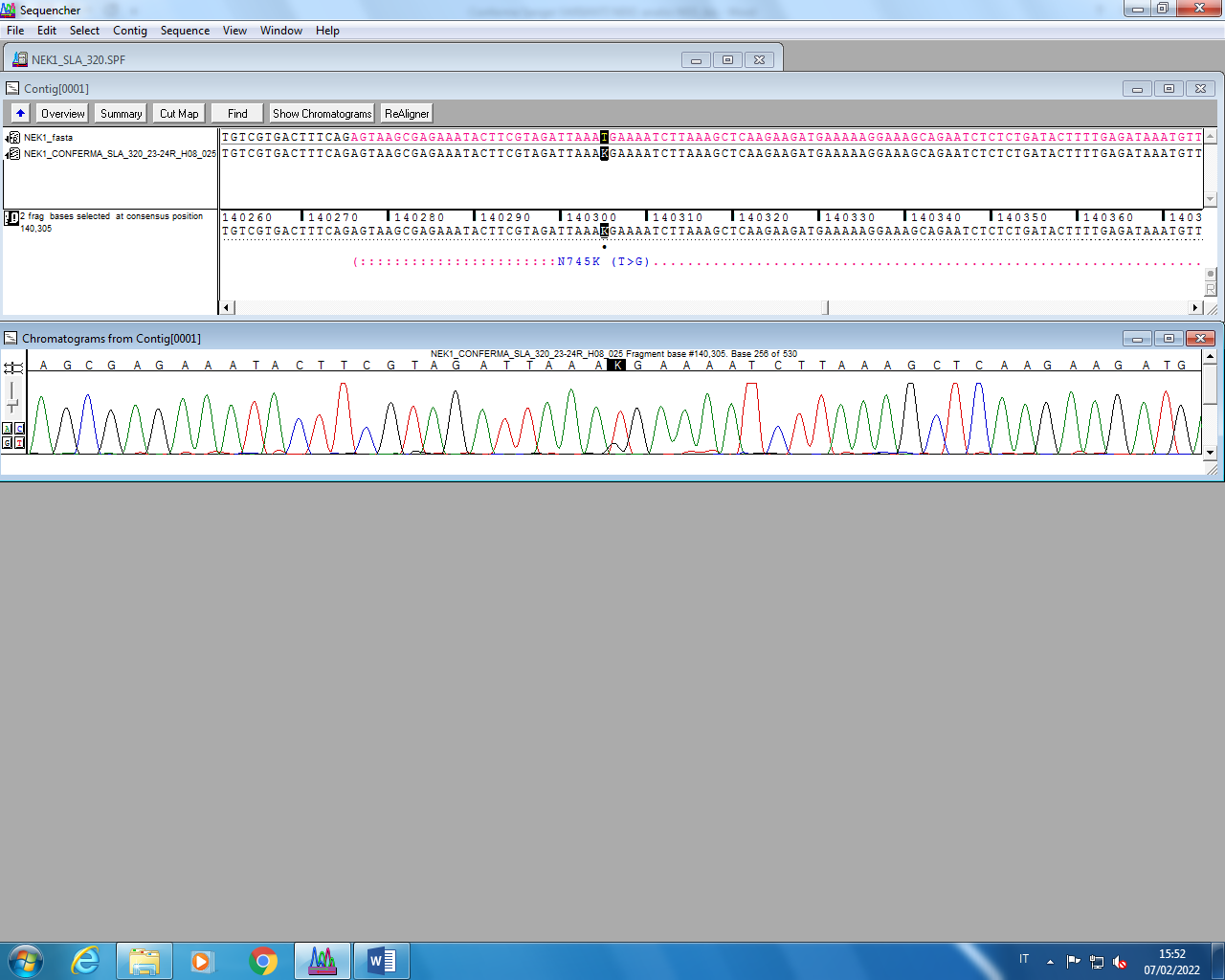


- ALS_470: p.Asn745Lys (T>G)


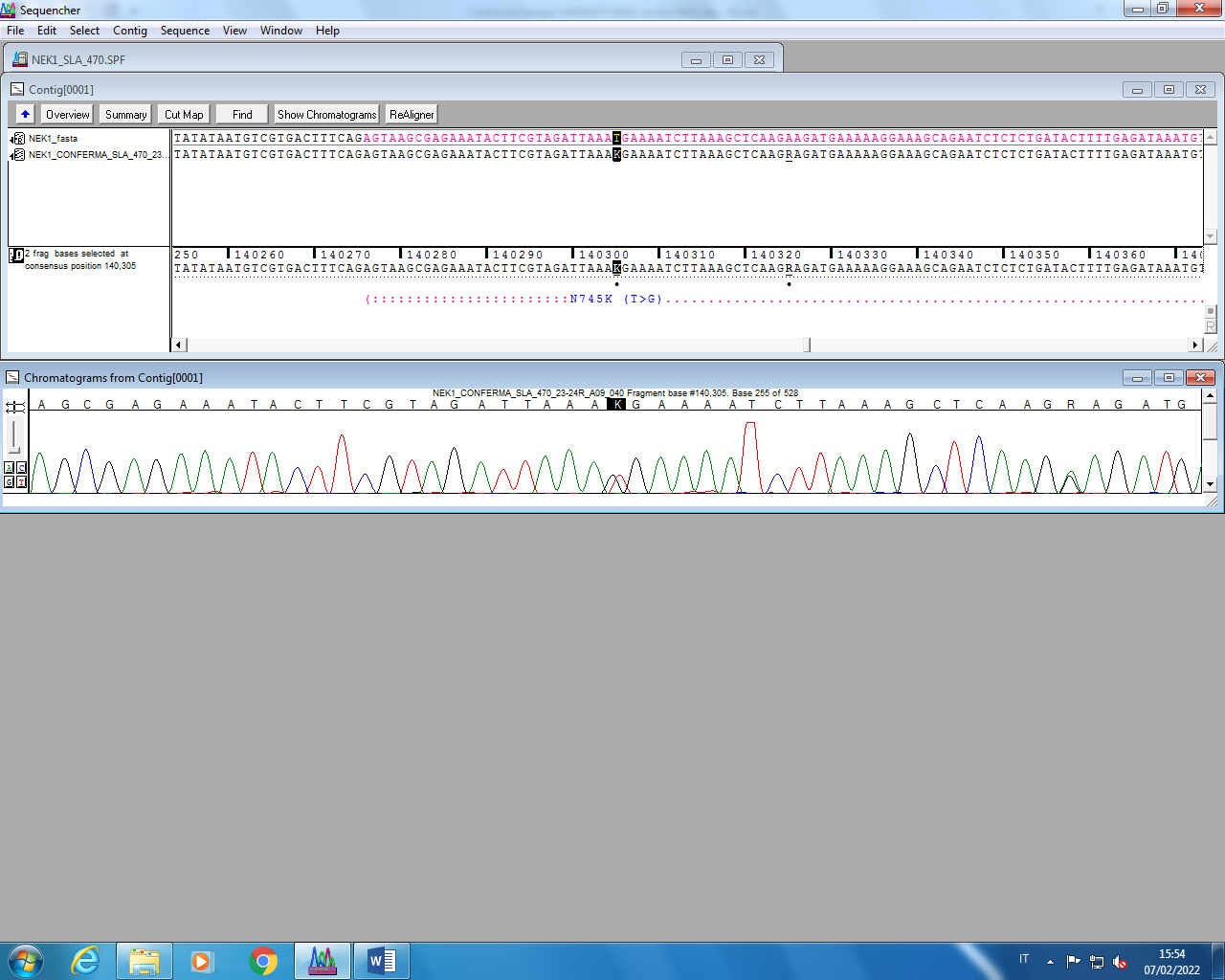


- ALS_945: p.Asn745Lys (T>G)


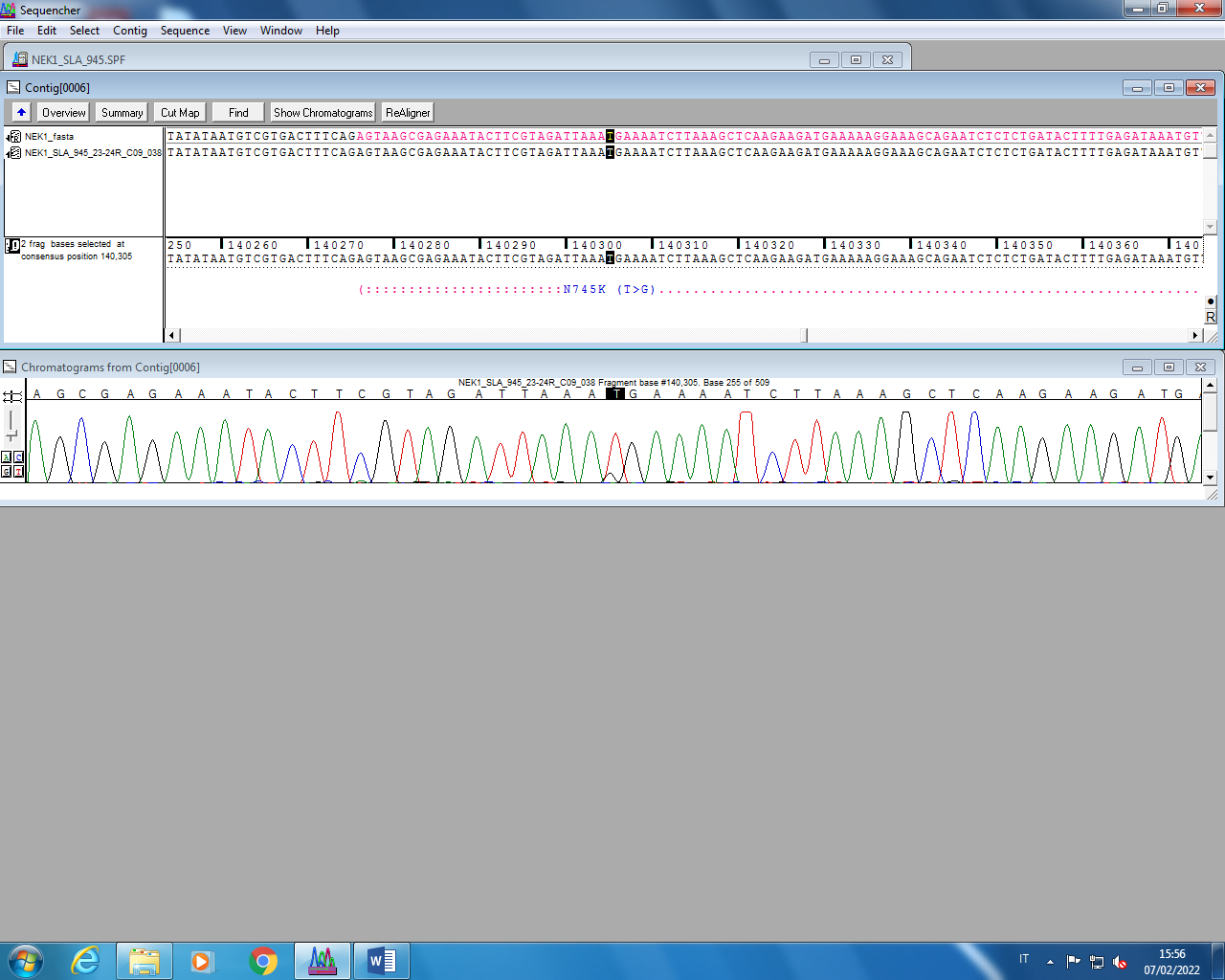


- ALS_66: p.Gly792Asp (G>A)


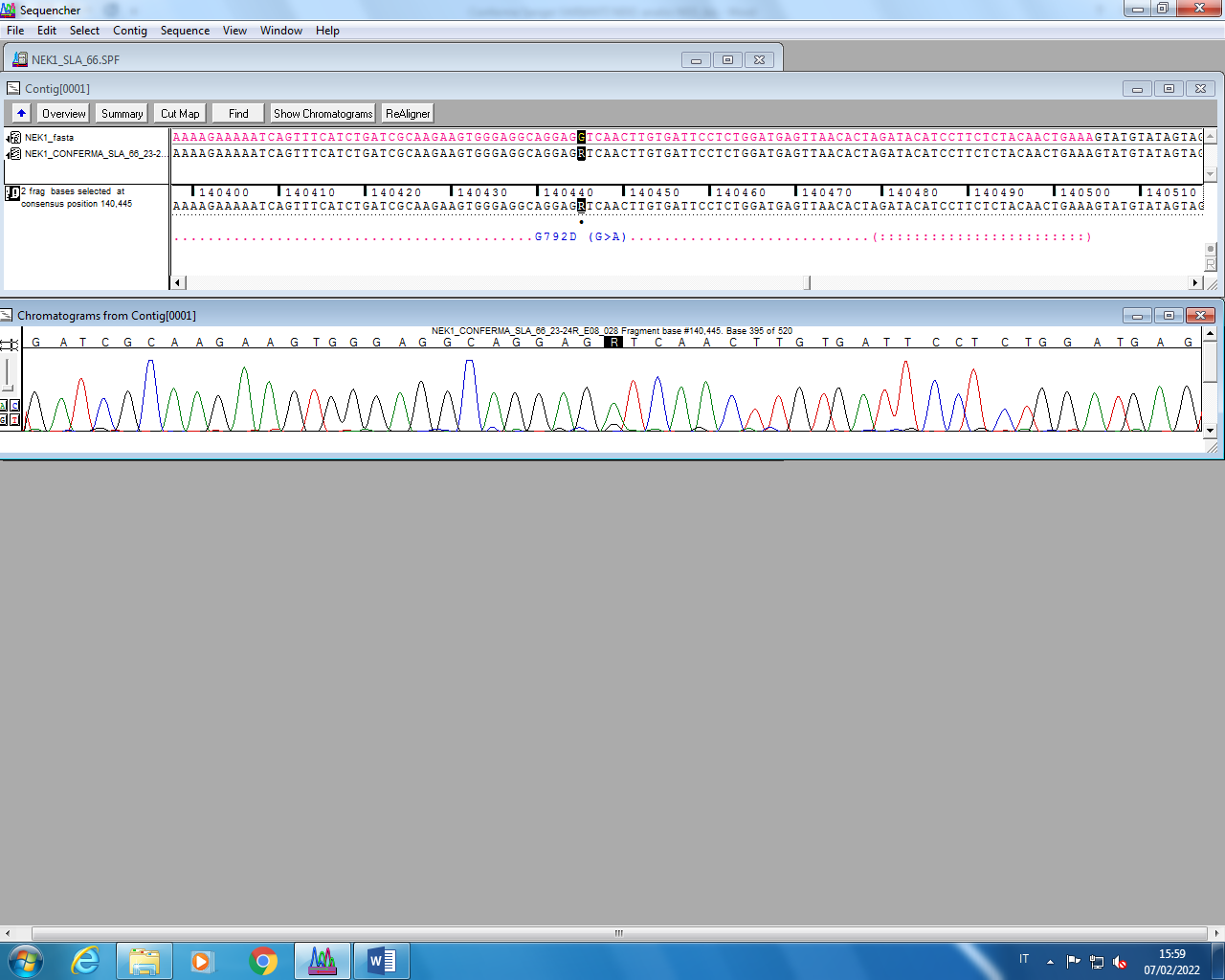


- ALS_441: p.Gln911Glu (C>G)


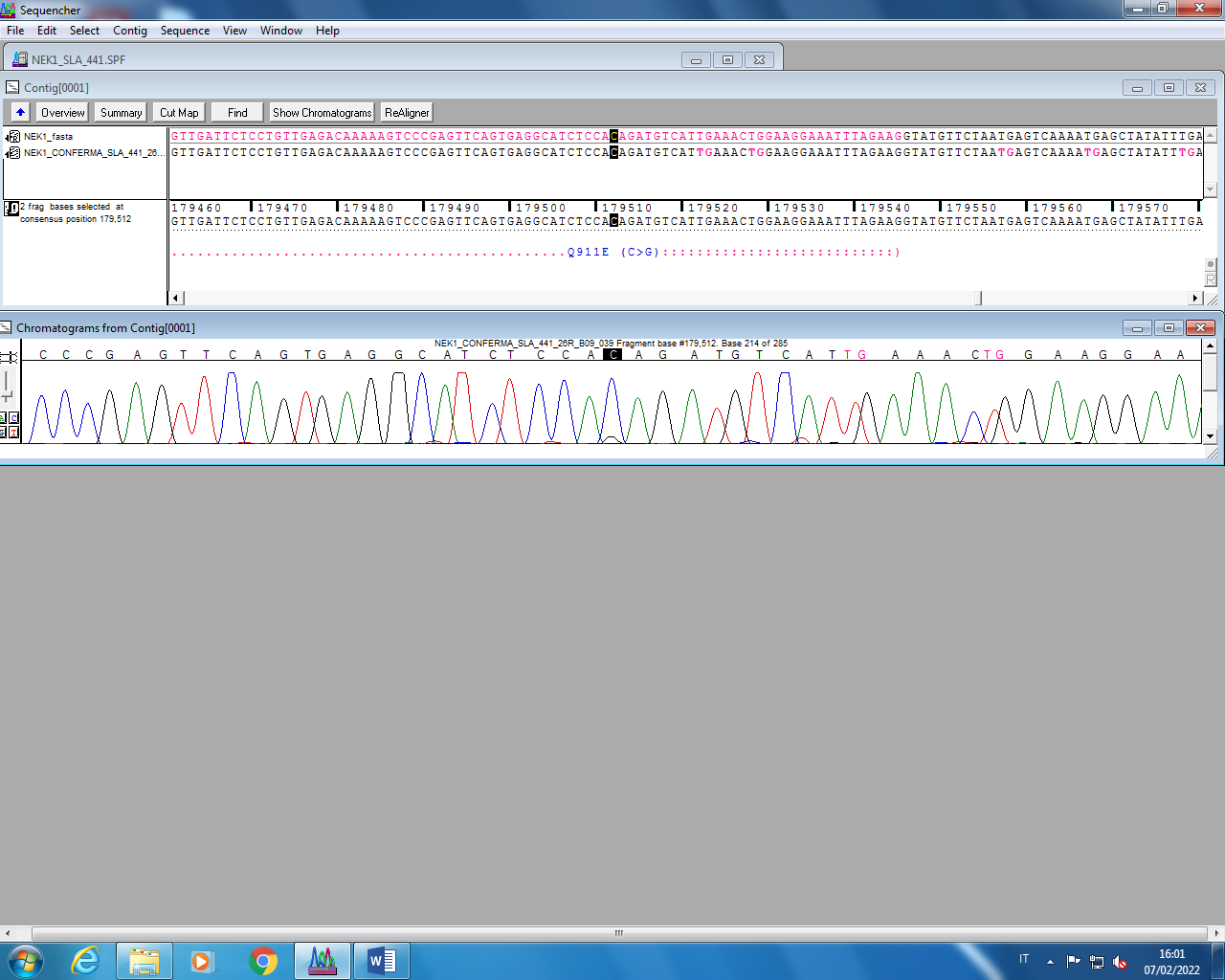


- ALS_862: p.Gln911Glu (C>G)


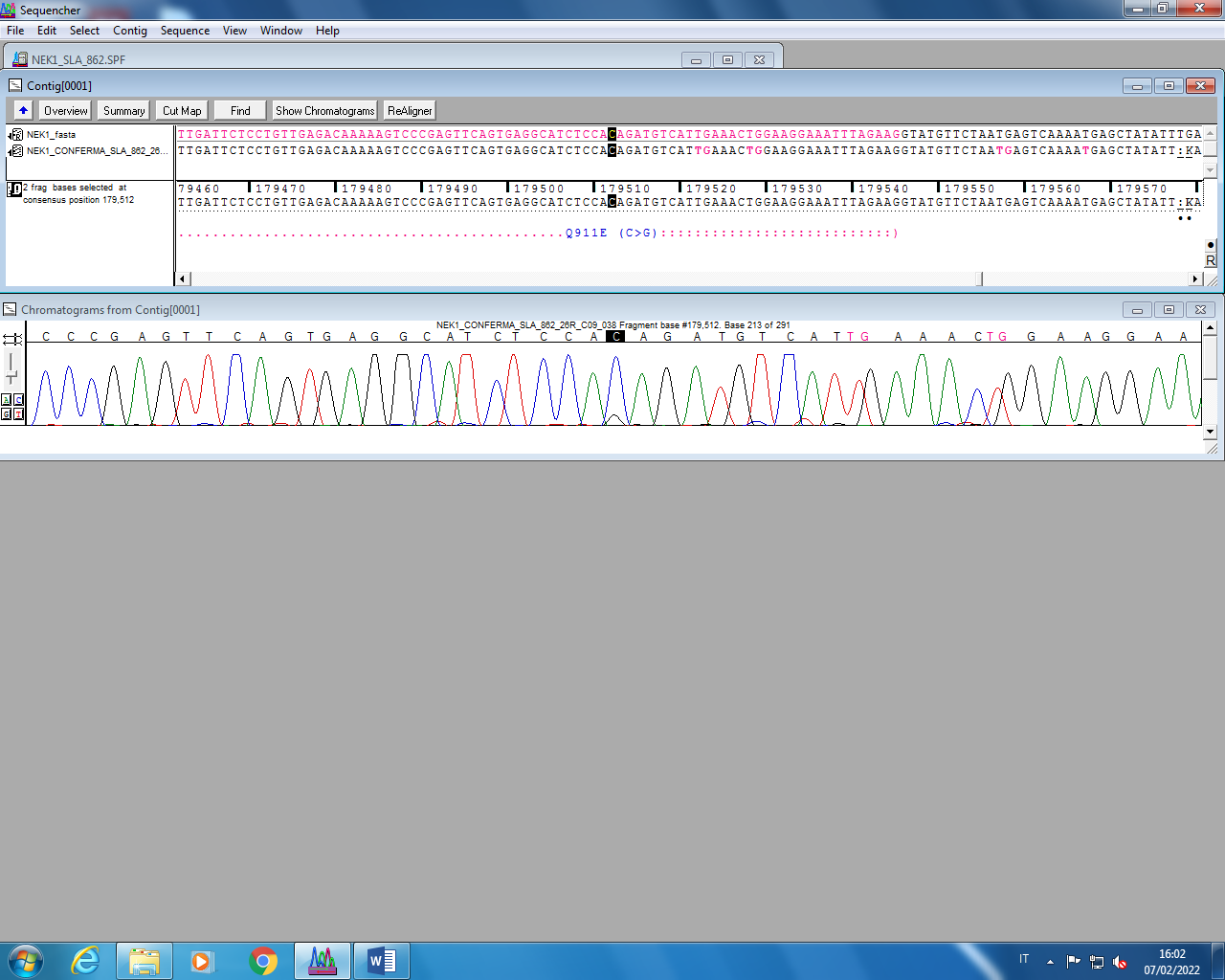


- ALS_751: p.Val1097Ile (G>A)


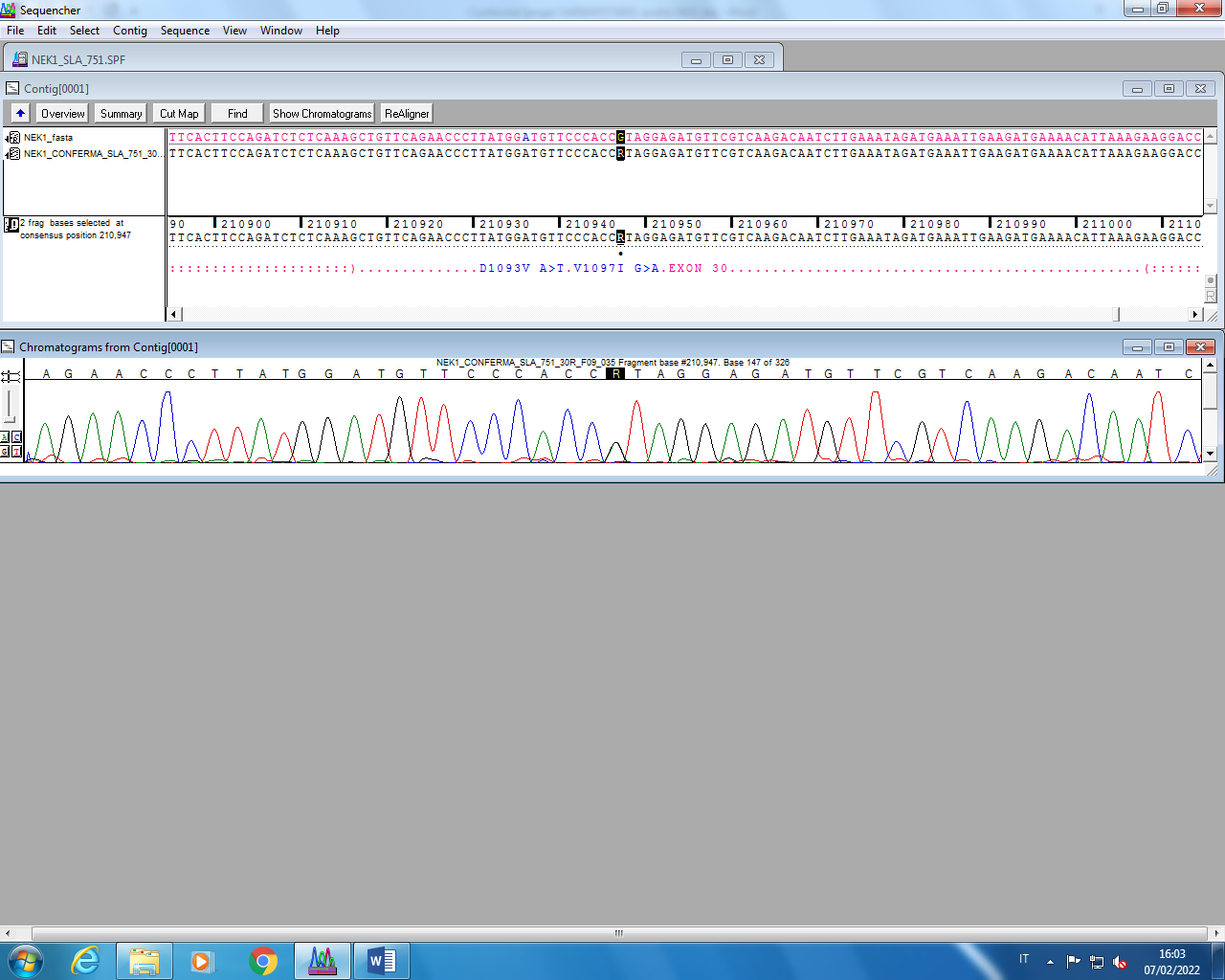


- ALS_601: p.Val1210Ile (G>A)


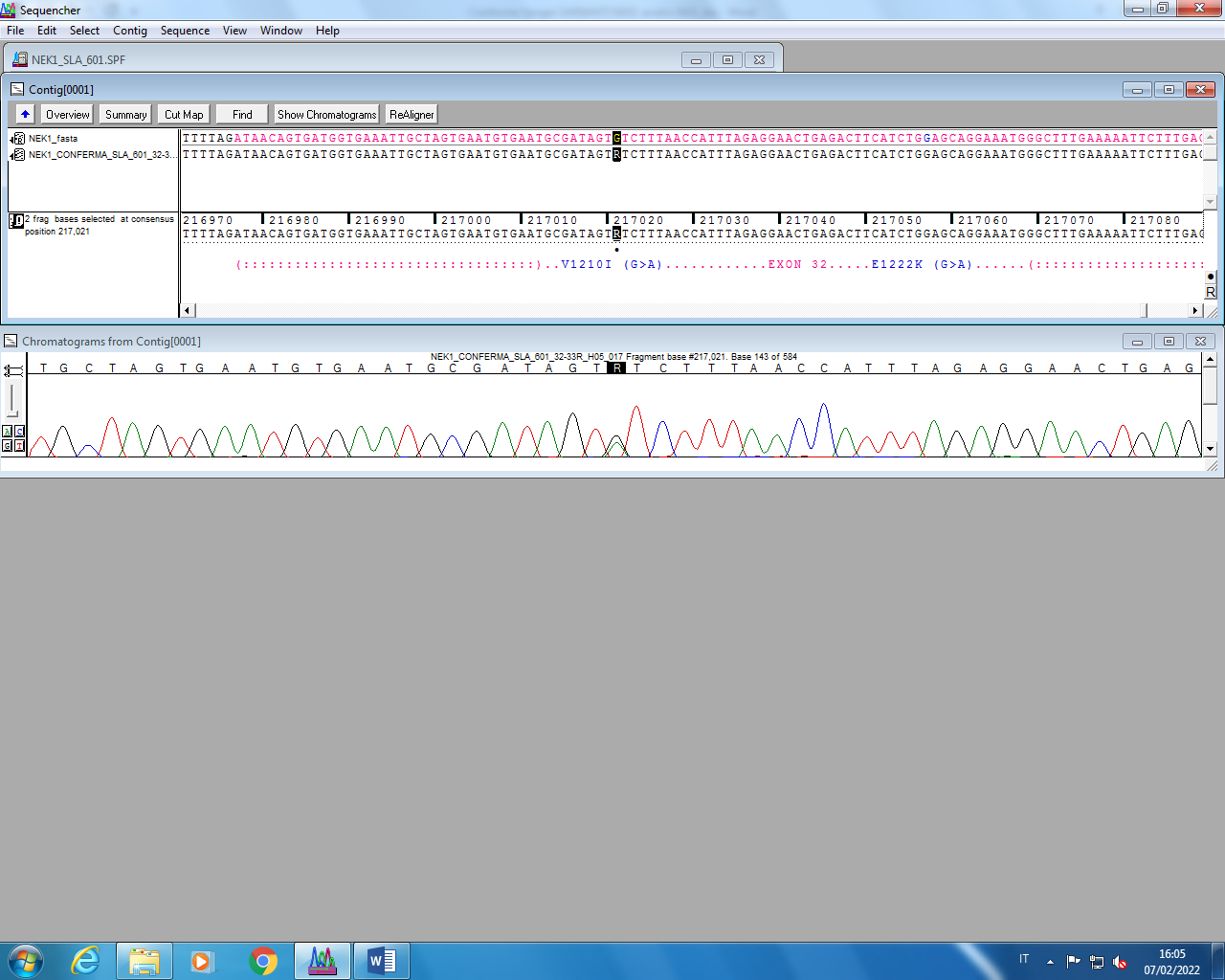


- ALS_518: p.Glu1222Lys (G>A)


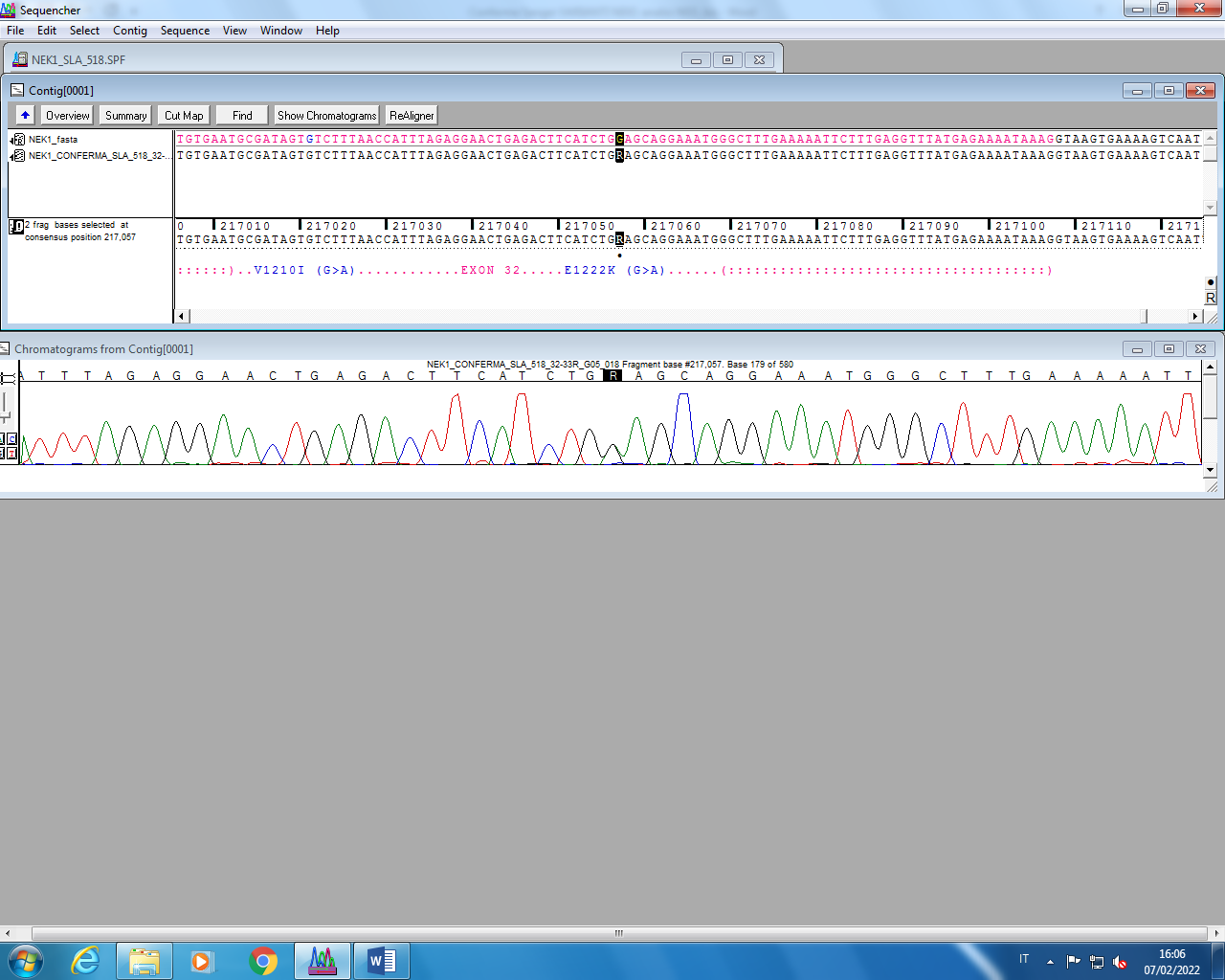


**Figure S3: Sanger validation of NEK1 variants found in families.**

- Family #1: c.3374+1G>A

(II:1)


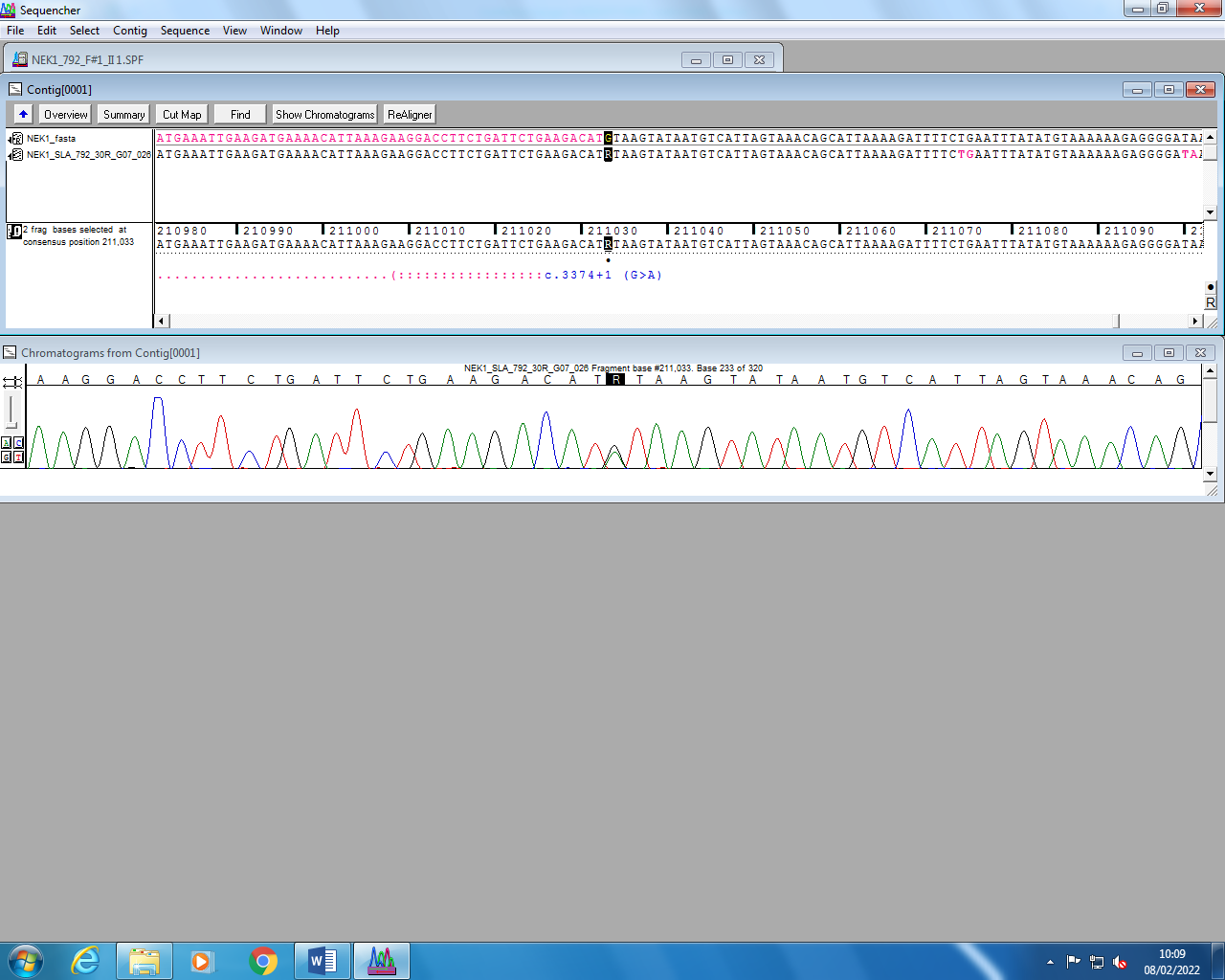


- Family #2: p.Cys113Arg (T>C)

(I:2; II:1)


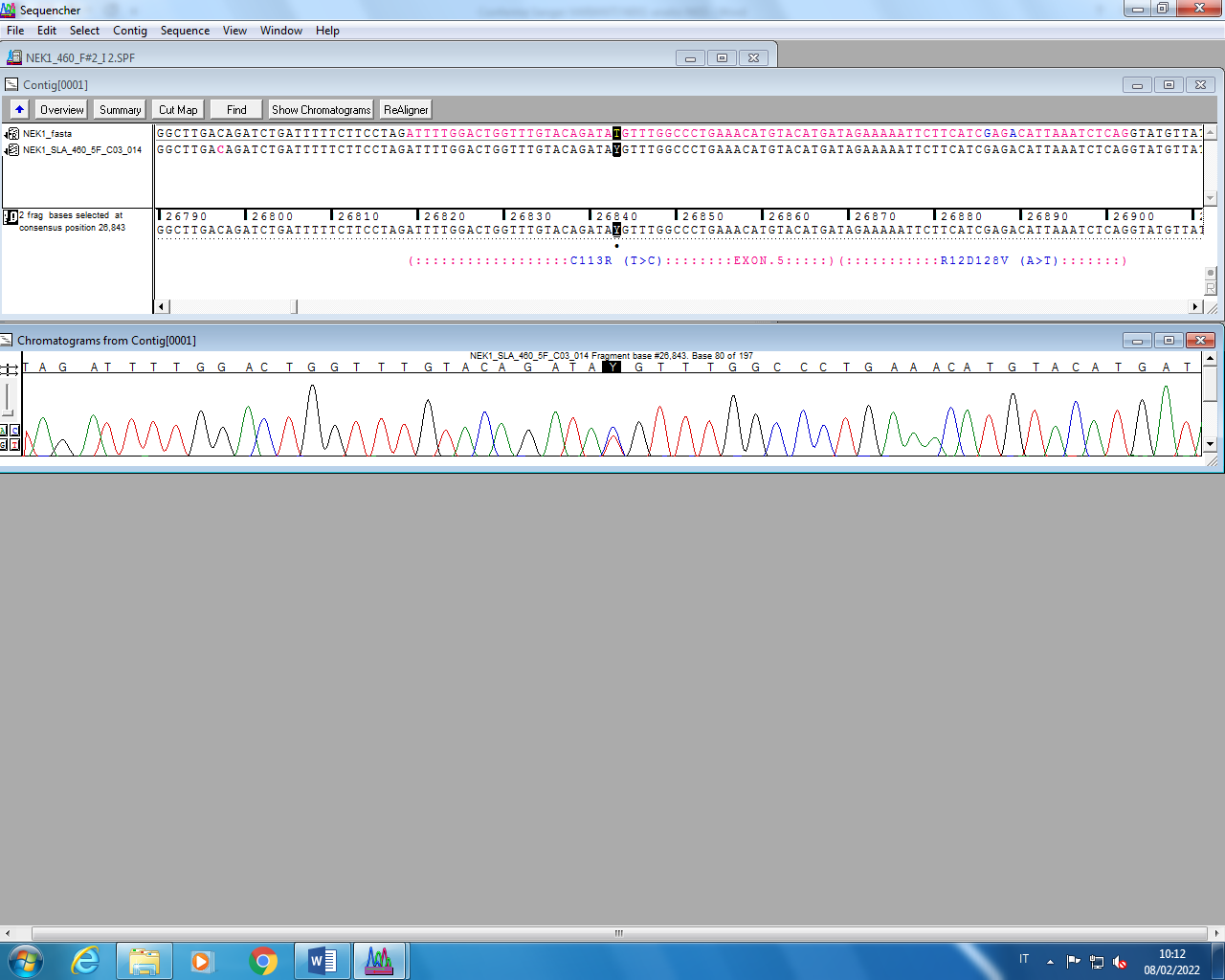


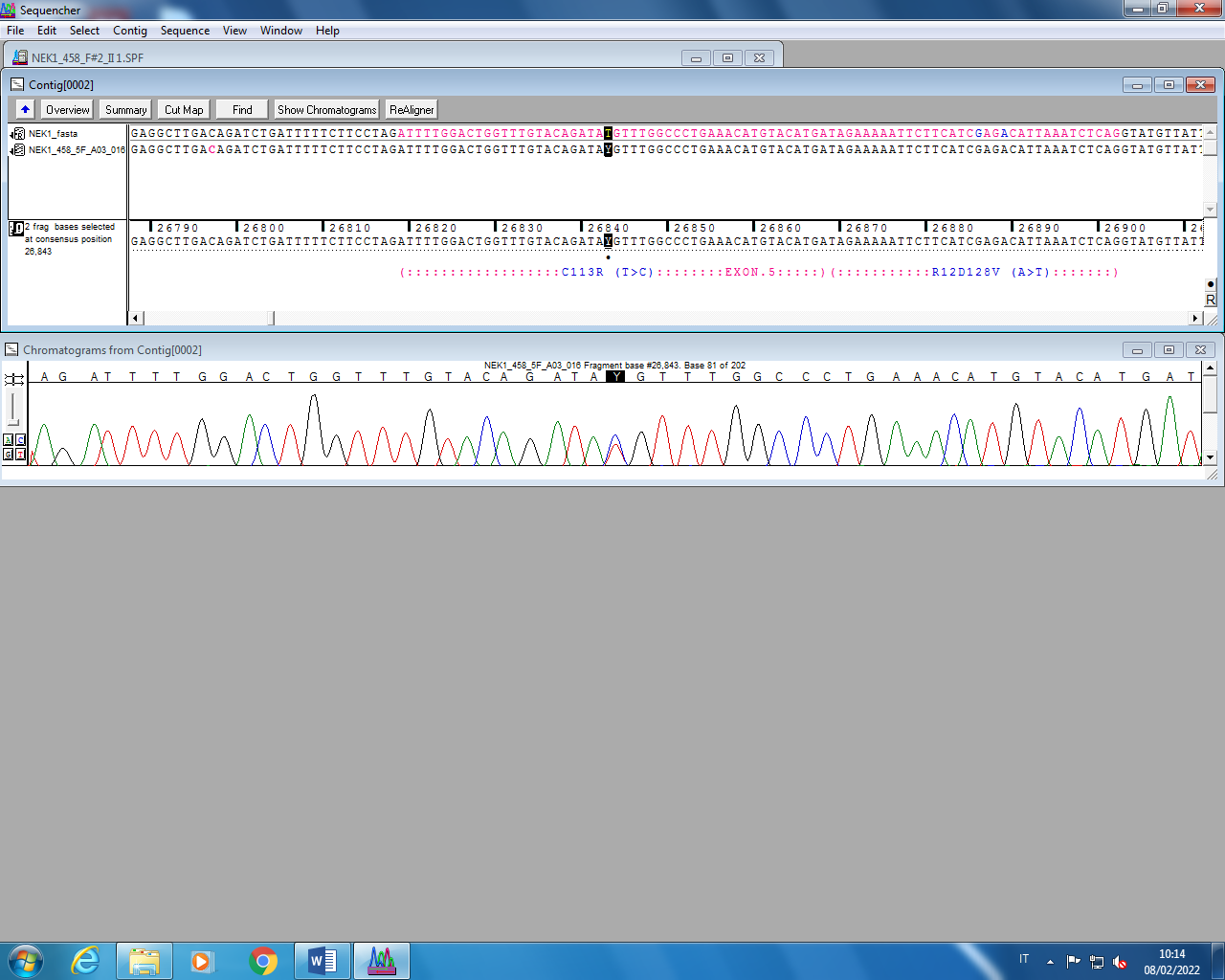


- Family #3: p.Asn732Ser (A>G)

(II:1, II:2)


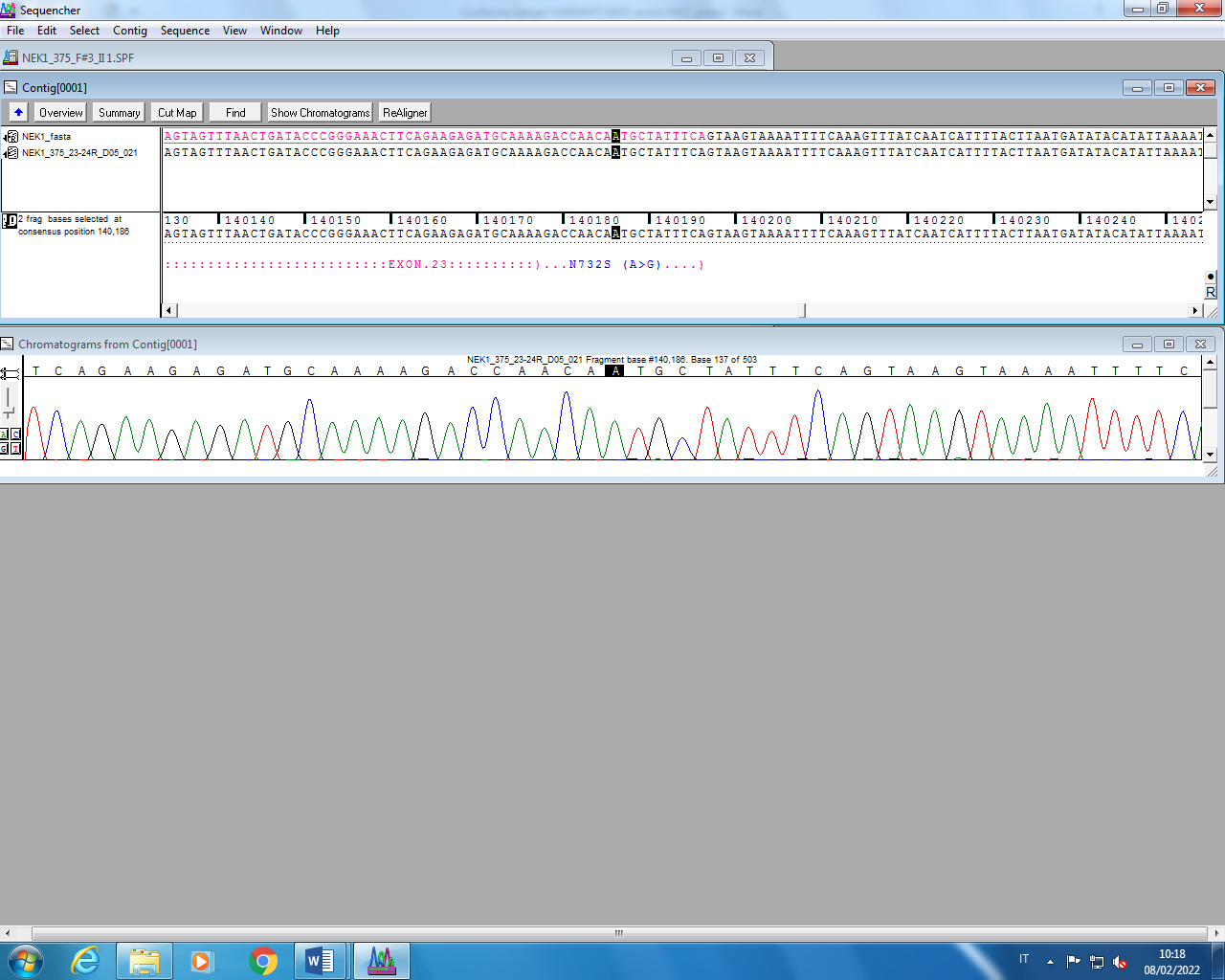


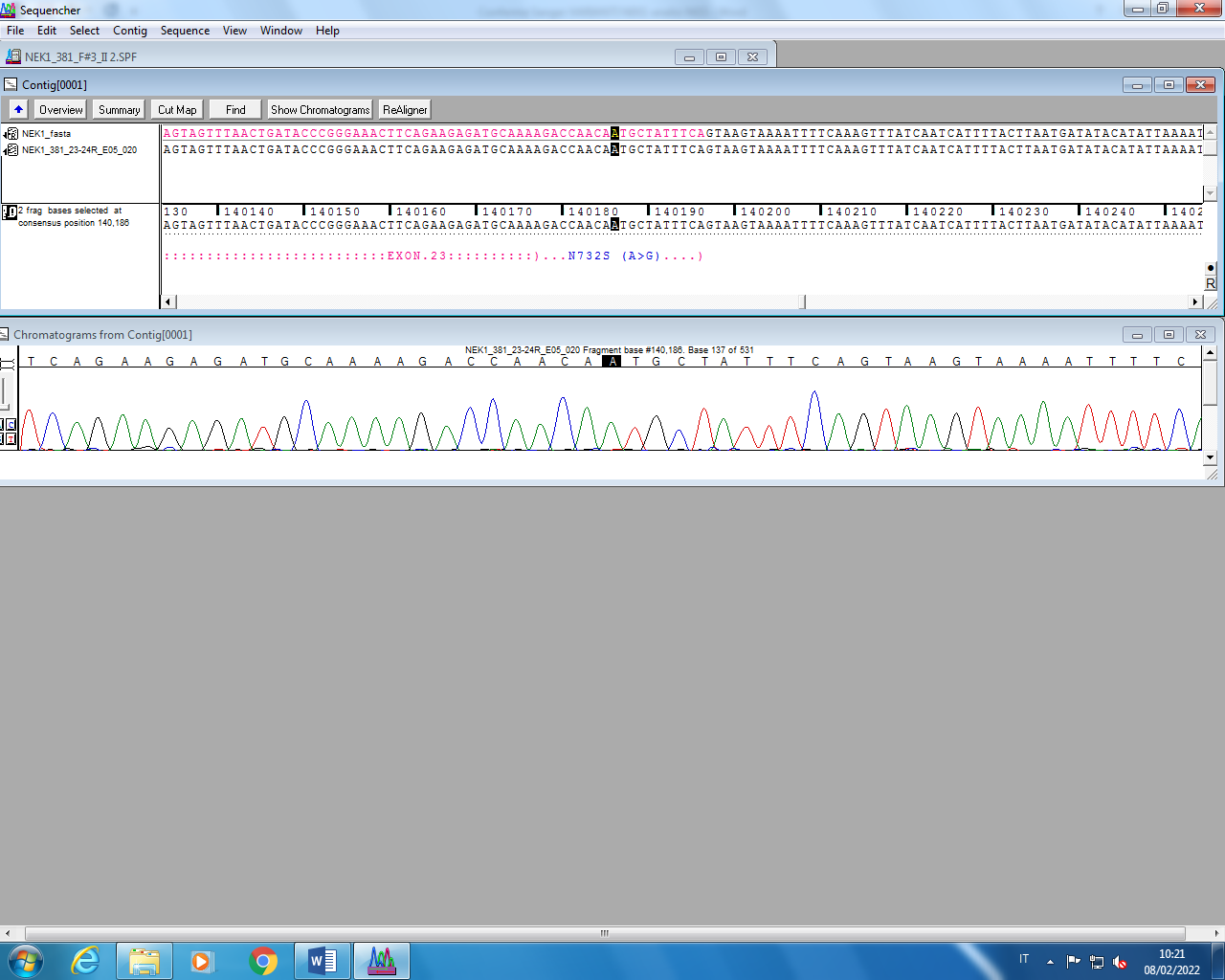


- Family #4: p.Arg261His (G>A)

(II:1)


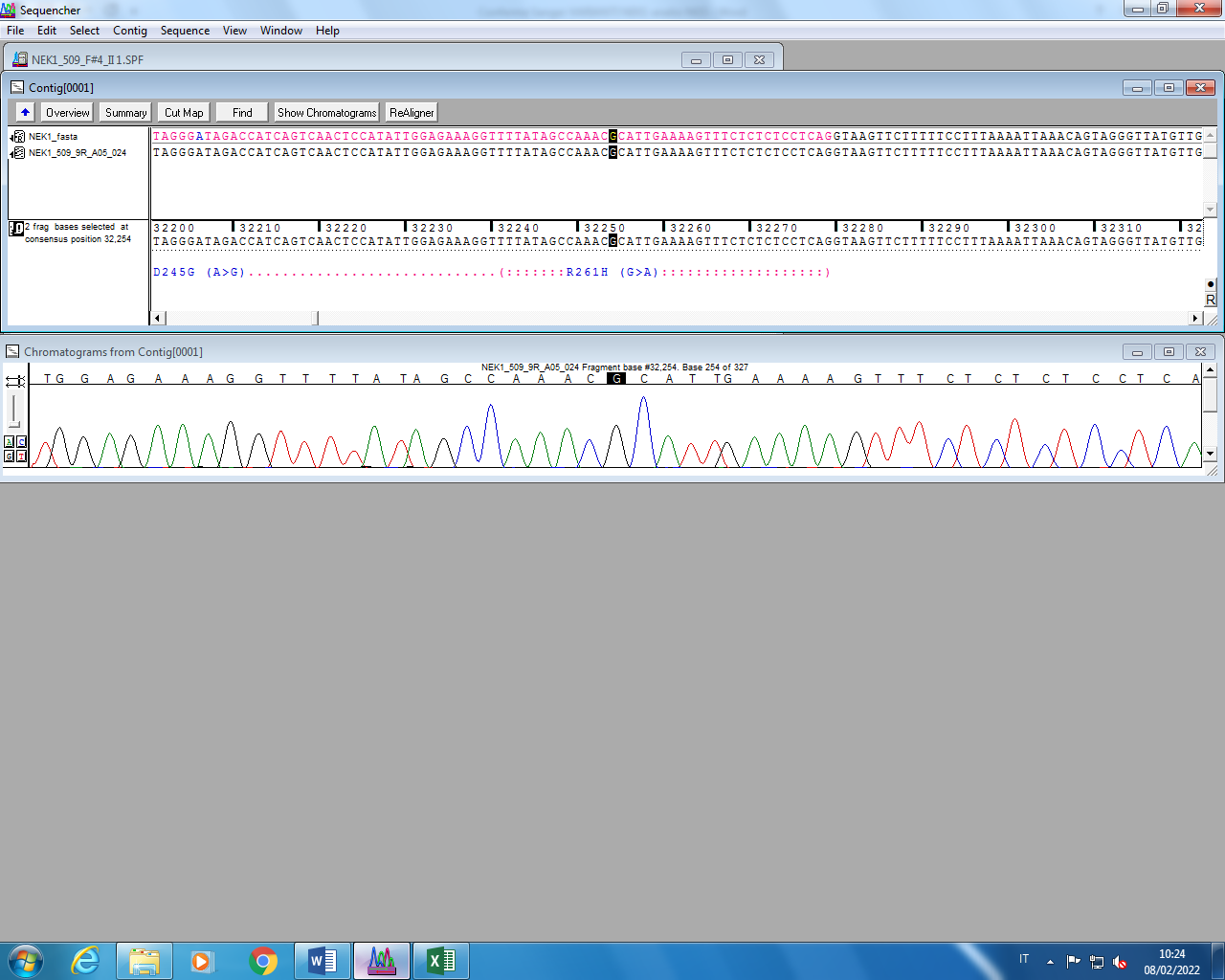


**Table S1: Primer sequences for Sanger confirmation of the *NEK1* variants found in NGS analysis**

| exon 5 | 5’-ggatttagttcctctcatcc-3’ |
| --- | --- |
|  | 5’-caatgaaggttccatgtctt-3’ |
| exon 7 | 5’-gaccagcatcatatatgc-3’ |
|  | 5’-gaatagtattccaaaggtta-3’ |
| exon 9 | 5’-gaactatttctcattaccgat-3’ |
|  | 5’-cctccaagatccagatgtgt-3’ |
| exon 17 | 5’-gtatggctatgttctgcaga-3’ |
|  | 5’-ggtactaagctatgtatgac-3’ |
| exon 21 | 5’-gtaaatatcttcatagacctg-3’ |
|  | 5’-ctgatgtactacccaggaag-3’ |
| exon 23-24 | 5’-gtaacgaggatgtaatattt-3’ |
|  | 5’-ctcaattcttctaagtgttc-3’ |
| exon 26 | 5’-ccaatgccattacagataaa-3’ |
|  | 5’-ctgtttataaagcttgtgaa-3’ |
| exon 29 | 5’-gaatttgtgttacatactgt-3’ |
|  | 5’-gtctgtgttaagagattaaa-3’ |
| exon 30 | 5’-gtgattagttctagttttct-3’ |
|  | 5’-cagaagacataaaagcagag-3’ |
| exon 32-33 | 5’-caatggcattattcatttgt-3’ |
|  | 5’-gtgatgtaagctgattcata-3’ |

**Table S2: In-silico splicing predictions**

| Variant | SSF [0-100] (wt→mut) | MES [0-12]  ( wt→mut) | NNS [0-1] (wt→mut) |
| --- | --- | --- | --- |
| c.3222+1G>A (donor site) | 99.77 → 0 | n.d. | n.d. |
| c.3374+1G>A (donor site) | 87.38 → 0 | 8.31 → 0 | 0.99 → 0 |

Legend: SSF: Splice Site Finder; MES: Max Ent Scan; NNS: Neural Network Site; n.d., not detected

**Table S3: *NEK1* missense variants found only in control cases**

| **cDNA Change** | **Protein Change** | **dbSNP ID^a^** | **ACMG** | **Global MAF^b^** | **Population MAF^c^** | **Nr. Controls^d^** |
| --- | --- | --- | --- | --- | --- | --- |
| c.336A>G | p.Ile112Met | rs764374761 | 3 | 0,000007 | 0,00015 | 1 |
| c.482G>A | p.Arg161Gln | rs1041766237 | 3 | 0,00042 | 0,00016 | 1 |
| c.937G>A | p.Ala313Thr ^¶^ | rs775516158 | 3 | 0,00028 | 0,00016 | 1 |
| c.1021G>A | p.Ala341Thr ^§^ | rs189186475 | 3 | 0.002 | 0.003 | 2 |
| c.2137G>A | p.Val713Met ^§^ | rs199827465 | 2 | 0.001 | 0.001 | 1 |

Legend: a dbSNP150; b Global MAF, global allele counts were calculated from all subjects in the GnomAD database; c MAF population, population allele count refers to European Ancestry subjects from the GnomAD database; d Nr. Controls, number of controls carrying the variant. Key: §, variants already described in literature in both patients with ALS and control cases; ¶, variants already described in literature in patients with ALS. See table S5 for details. ACMG, American College of Medical Genetics and Genomics; ACMG classification: 1 = benign, 2 = likely benign, 3 = uncertain significance, 4 = likely pathogenic, 5 = pathogenic; MAF, minor allele frequency;

**Table S4: Summary data of review of the literature of *NEK1* variants found in both ALS patients and controls (**[**Brenner, Muller et al. 2016**](#_ENREF_2)**;** [**Kenna, van Doormaal et al. 2016**](#_ENREF_5)**;** [**Black, Leighton et al. 2017**](#_ENREF_1)**;** [**Gratten, Zhao et al. 2017**](#_ENREF_4)**;** [**Nguyen, Van Mossevelde et al. 2018**](#_ENREF_10)**;** [**Shu, Lei et al. 2018**](#_ENREF_15)**;** [**Naruse, Ishiura et al. 2019**](#_ENREF_9)**;** [**Tsai, Lin et al. 2020**](#_ENREF_16)**;** [**Zhang, Lu et al. 2020**](#_ENREF_17)**;** [**Lattante, Doronzio et al. 2021**](#_ENREF_6)**).**

|  | ALS* | ALS+CTRL* | CTRL* |
| --- | --- | --- | --- |
| All | 107 (100) | 6036 (38) | 94 (81) |
| Lof | 51 (47) | 30 (4) | 9 (9) |
| missense | 56 (53) | 6006 (34) | 85 (72) |
| R261H | - | 134 | - |

Legend: *Number of patients (Number of different variants).

Key: ALS, amyotrophic lateral sclerosis; CTRL, non-neurological unrelated controls. LoF, loss of function

**Table S5: Review of the literature of *NEK1* variants in the ALS-FTD spectrum reported so far**

| **Protein Change** | **Reference (year)** | **ALS**  **(nr. subjects)** | **CTRL**  **(nr. subjects)** |
| --- | --- | --- | --- |
| **LoF variants** | | | |
| p.S14Sfs*45 | Brenner (2016) | 1 | - |
| p.F15Sfs*10 | Brenner (2016) | - | 1 |
| p.Q132R* | Kenna (2016) | 1 | - |
| p.N133Hfs*4 | Naruse (2020) | 1 | - |
| p.E158fs | Kenna (2016) | 1 | - |
| p.R161* | Kenna (2016), Gratten (2017), Black (2017) | 3 | 1 |
| p.D185Lfs*6 | Naruse (2020) | 1 | - |
| p.F220fs | Kenna (2016) | 1 | - |
| p.H330fs | Kenna (2016) | 1 | - |
| p.Q343* | Kenna (2016) | 1 | - |
| p.K376Tfs*7 | Tsai (2020) | 1 | - |
| p.Q380E* | Kenna (2016) | 1 | - |
| p.Q393* | Kenna (2016) | - | 1 |
| p.W409* | Kenna (2016) | 1 | 1 |
| p.R440X | Gratten (2017) | 1 | - |
| p.Q455* | Naruse (2020) | 1 | - |
| p.P498Lfs*10 | Tsai (2020) | 1 | - |
| p.R540* | Kenna (2016) | 2 | - |
| p.R550* | Kenna (2016) | 1 | - |
| p.E581Rfs*18 | Naruse (2020) | - | 1 |
| p.I633Nfs*28 | Tsai (2020) | 1 | - |
| p.Q650fs | Black (2017) | 1 | - |
| p.R653* | Gratten (2017), Naruse (2020) | 2 | - |
| p.R655fs | Kenna (2016) | 1 | - |
| p.K656Rfs*41 | Naruse (2020) | 1 | - |
| p.Y659* | Tsai (2020) | - | 1 |
| p.E662fs | Kenna (2016) | 2 | - |
| p.V665Cfs*34 | Tsai (2020) | 1 | - |
| p.Q696* | Kenna (2016) | 1 | - |
| p.R738* | Shu (2018) | 1 | - |
| p.S761fs | Kenna (2016) | 1 | - |
| p.E778* | Naruse (2020) | 1 | - |
| p.K779fs | Gratten (2017) | 2 | - |
| p.W788* | Shu (2018) | 1 | - |
| p.R812* | Brenner (2016) | 1 | - |
| p.L854Yfs*2 | Nguyen (2017) | 1 | - |
| p.Y871fs | Kenna (2016) | 1 | - |
| p.Y871Vfs*17 | Nguyen (2017) | 1 | - |
| p.V881Yfs*8 | Tsai (2020) | 1 | - |
| p.L920* | Kenna (2016) | 1 | - |
| p.E929Nfs*12 | Lattante (2021) | 1 | - |
| p.N938fs | Gratten (2017) | 1 | - |
| p.V1030Ifs*23 | Lattante (2021) | 1 | - |
| p.S1036* | Kenna (2016), Brenner (2016), Nguyen (2017) | 15 | 6 |
| p.D1112* | Naruse (2020) | 1 | - |
| p.P1119fs | Gratten (2017) | 1 | - |
| p.I1125fs | Gratten (2017) | 1 | - |
| p.K1210* | Zhang (2020) | 1 | - |
| c.214+1G>A | Black (2017) | 1 | - |
| c.217+1C>T | Kenna (2016) | - | 1 |
| c.877-1C>A | Kenna (2016) | 1 | - |
| c.878+1C>G | Kenna (2016) | 1 | - |
| c.1153+3T>G | Kenna (2016) | 1 | - |
| c.1446+1C>A | Kenna (2016) | - | 1 |
| c.1683+2A>G | Kenna (2016) | - | 1 |
| c.1750-5T>C | Black (2017) | 1 | - |
| c.1911+1->TATA | Black (2017) | 1 | - |
| c.2458-2T>C | Kenna (2016) | - | 1 |
| c.2588-2A>G | Shu (2018), Tsai (2020) | 2 | 1 |
| c.2612-2T>C | Kenna (2016) | - | 1 |
| **Missense variants** | | | |
| p.M1? | Lattante (2021) | 1 | - |
| p.K3E | Kenna (2016) | - | 1 |
| p.I10T | Kenna (2016) | 1 | 1 |
| p.Y30C | Kenna (2016) | 1 | - |
| p.I37V | Nguyen (2017) | - | 1 |
| p.V64A | Kenna (2016) | 1 | - |
| p.R91Q | Kenna (2016) | - | 1 |
| p.N93D | Kenna (2016) | 1 | 1 |
| p.V98I | Kenna (2016) | - | 3 |
| p.Q101R | Naruse (2020) | 1 | - |
| p.I105T | Kenna (2016) | 1 | - |
| p.L106W | Nguyen (2017) | - | 1 |
| p.A115S | Kenna (2016) | - | 1 |
| p.A115V | Gratten (2017) | 1 | - |
| p.H120R | Gratten (2017) | 1 | - |
| p.I129S | Nguyen (2017), Black (2017) | 1 | 1 |
| p.I149L | Naruse (2020) | 1 | - |
| p.N154Y | Tsai (2020) | 1 | 1 |
| p.N181S | Brenner (2016) | 1 | - |
| p.D185G | Kenna (2016) | 2 | - |
| p.L193F | Kenna (2016), Lattante (2021) | 1 | 1 |
| p.E204G | Kenna (2016) | - | 1 |
| p.V212G | Nguyen (2017) | - | 1 |
| p.V223M | Kenna (2016) | - | 1 |
| p.S224F | Kenna (2016) | - | 1 |
| p.Y229C | Kenna (2016), Tsai (2020) | 2 | 12 |
| p.R232C | Nguyen (2017) | 1 | - |
| p.R232H | Black (2017) | - | 1 |
| p.P243A | Kenna (2016) | 1 | - |
| p.E254K | Gratten (2017) | 1 | - |
| p.F257C | Tsai (2020) | - | 1 |
| p.I258T | Tsai (2020) | 1 | - |
| p.I258R | Tsai (2020) | 1 | - |
| p.R261C | Kenna (2016) | 1 | - |
| p.R261H | Kenna (2016), Brenner (2016), Nguyen (2017), Black (2017), Lattante (2021) | 70 | 64 |
| p.C276F | Black (2017) | - | 1 |
| p.P287A | Shu (2018) | - | 5 |
| p.I300T | Naruse (2020) | - | 2 |
| p.I308M | Kenna (2016) | - | 1 |
| p.A313T | Kenna (2016) | 1 | - |
| p.K332T | Gratten (2017) | 1 | - |
| p.K337N | Kenna (2016) | - | 1 |
| p.A341T | Kenna (2016), Brenner (2016), Nguyen (2017), Black (2017), Lattante (2021) | 20 | 50 |
| p.Q343R | Gratten (2017) | - | 1 |
| p.T344A | Kenna (2016), Lattante (2021) | 1 | 1 |
| p.R356K | Kenna (2016) | 1 | 1 |
| p.D379E | Kenna (2016), Black (2017) | 4 | 5 |
| p.Q380E | Lattante (2021) | 1 | - |
| p.L384S | Kenna (2016) | - | 1 |
| p.G399A | Brenner (2016) | 1 | - |
| p.K422E | Kenna (2016) | 1 | - |
| p.S428N | Naruse (2020) | - | 1 |
| p.R440Q | Kenna (2016) | - | 1 |
| p.Y443C | Kenna (2016) | 1 | - |
| p.E444D | Kenna (2016) | 1 | - |
| p.E462V | Kenna (2016) | 1 | - |
| p.A463V | Kenna (2016) | 382 | 914 |
| p.G478A | Kenna (2016) | 1 | - |
| p.G478E | Gratten (2017) | 1 | - |
| p.R484G | Shu (2018) | 1 | - |
| p.K511Q | Kenna (2016) | 1 | - |
| p.A512V | Kenna (2016) | - | 1 |
| p.A512T | Naruse (2020) | 1 | 2 |
| p.Q525K | Kenna (2016) | - | 1 |
| p.E529G | Tsai (2020) | 1 | - |
| p.R542Q | Tsai (2020) | - | 1 |
| p.M545T | Kenna (2016), Brenner (2016), Tsai (2020) | 3 | 1 |
| p.Q546R | Lattante (2021) | 1 | - |
| p.M555I | Lattante (2021) | 1 | - |
| p.R568K | Tsai (2020) | - | 1 |
| p.Y585C | Naruse (2020) | - | 1 |
| p.L586P | Shu (2018) | 1 | - |
| p.R590K | Tsai (2020) | - | 1 |
| p.P596R | Kenna (2016) | - | 1 |
| p.F597I | Kenna (2016), Black (2017) | - | 2 |
| p.N598S | Kenna (2016) | 3 | 2 |
| p.K606R | Nguyen (2017) | - | 1 |
| p.R608H | Naruse (2020), Tsai (2020) | 1 | 2 |
| p.R608C | Tsai (2020) | 2 | - |
| p.E610K | Kenna (2016) | - | 1 |
| p.E624K | Kenna (2016) | - | 1 |
| p.A626T | Kenna (2016) | 527 | 1271 |
| p.R630H | Kenna (2016), Lattante (2021) | 1 | 1 |
| p.R643G | Shu (2018) | 1 | - |
| p.R643H | Naruse (2020) | - | 1 |
| p.V646I | Kenna (2016) | 1 | 1 |
| p.K648E | Kenna (2016), Lattante (2021) | 2 | 2 |
| p.K654M | Kenna (2016) | - | 1 |
| p.E660K | Tsai (2020) | - | 1 |
| p.R661K | Tsai (2020) | - | 1 |
| p.E662D | Naruse (2020) | - | 1 |
| p.E662K | Tsai (2020) | - | 1 |
| p.W666C | Tsai (2020) | 1 | - |
| p.V675I | Kenna (2016) | - | 1 |
| p.S681F | Kenna (2016) | - | 1 |
| p.P682L | Kenna (2016) | 3 | 2 |
| p.V704I | Brenner (2016), Kenna (2016) | 4 | 4 |
| p.V713M | Kenna (2016), Brenner (2016), Nguyen (2017), Black (2017), Lattante (2021) | 6 | 22 |
| p.Q728P | Tsai (2020) | - | 1 |
| p.N732S | Gratten (2017), Tsai (2020) | 1 | 1 |
| p.A733G | Nguyen (2017) | - | 1 |
| p.I734N | Nguyen (2017) | - | 1 |
| p.S735del | Nguyen (2017) | - | 1 |
| p.R742C | Kenna (2016) | 2 | 8 |
| p.N745K | Kenna (2016), Brenner (2016), Nguyen (2017), Black (2017) | 52 | 98 |
| p.N745S | Gratten (2017) | 1 | - |
| p.E752G | Kenna (2016) | 674 | 1742 |
| p.T763P | Tsai (2020) | - | 1 |
| p.H769R | Kenna (2016), Black (2017) | - | 3 |
| p.H775Y | Kenna (2016) | - | 2 |
| p.H775D | Gratten (2017) | 1 | - |
| p.D784E | Kenna (2016) | - | 1 |
| p.A790T | Black (2017) | - | 1 |
| p.G791R | Kenna (2016) | - | 1 |
| p.G792D | Kenna (2016) | 2 | 1 |
| p.L798V | Black (2017) | 1 | - |
| p.L815F | Lattante (2021) | 1 | - |
| p.V818I | Gratten (2017) | 1 | - |
| p.N824D | Naruse (2020) | - | 1 |
| p.G825V | Kenna (2016) | 1 | 1 |
| p.P835L | Kenna (2016) | - | 2 |
| p.K841E | Kenna (2016) | - | 1 |
| p.A846T | Kenna (2016) | - | 1 |
| p.K870N | Kenna (2016) | - | 1 |
| p.S902G | Kenna (2016) | 1 | - |
| p.S909C | Lattante (2021) | 1 | - |
| p.Q911E | Kenna (2016) | - | 1 |
| p.M912I | Tsai (2020) | - | 1 |
| p.K939E | Kenna (2016) | - | 1 |
| p.C945Y | Kenna (2016) | - | 1 |
| p.Q962R | Kenna (2016) | - | 1 |
| p.Q985H | Tsai (2020) | 1 | - |
| p.L986F | Kenna (2016) | - | 1 |
| p.H990D | Gratten (2017) | - | 1 |
| p.P993A | Kenna (2016) | - | 1 |
| p.S1001Y | Shu (2018) | 1 | - |
| p.Q1031R | Kenna (2016) | - | 1 |
| p.S1047L | Black (2017) | 1 | - |
| p.P1050L | Tsai (2020) | - | 1 |
| p.T1065A | Nguyen (2017), Kenna (2016) | 3 | 11 |
| p.R1073C | Lattante (2021) | 1 | - |
| p.M1075L | Kenna (2016) | - | 1 |
| p.T1096S | Kenna (2016) | 1 | 1 |
| p.T1096I | Naruse (2020) | - | 1 |
| p.V1097I | Tsai (2020) | - | 1 |
| p.D1099H | Kenna (2016) | 1 | - |
| p.D1108H | Naruse (2020) | - | 2 |
| p.E1129G | Kenna (2016) | 1 | - |
| p.E1142Q | Kenna (2016) | 1 | - |
| p.R1146K | Kenna (2016), Lattante (2021) | 1 | 1 |
| p.D1180A | Kenna (2016) | - | 1 |
| p.N1189K | Kenna (2016) | 1 | - |
| p.F1211S | Kenna (2016) | - | 1 |
| p.E1222K | Lattante (2021) | 1 | - |
| p.Y1229R | Shu (2018) | 1 | - |
| p.H1265Y | Kenna (2016), Lattante (2021) | 1 | 1 |
| p.I1270S | Kenna (2016), Lattante (2021) | 2 | - |
| p.D1283G | Kenna (2016) | - | 1 |
| p.N1284D | Kenna (2016) | 2 | 2 |

**References:**

Black, H. A., D. J. Leighton, et al. (2017). "Genetic epidemiology of motor neuron disease-associated variants in the Scottish population (vol 51, pg e11, 2017)." Neurobiology of Aging **56**: 214-214.

Brenner, D., K. Muller, et al. (2016). "NEK1 mutations in familial amyotrophic lateral sclerosis." Brain **139**(Pt 5): e28.

Cady, J., P. Allred, et al. (2015). "Amyotrophic lateral sclerosis onset is influenced by the burden of rare variants in known amyotrophic lateral sclerosis genes." Ann Neurol **77**(1): 100-113.

Gratten, J., Q. Y. Zhao, et al. (2017). "Whole-exome sequencing in amyotrophic lateral sclerosis suggests NEK1 is a risk gene in Chinese." Genome Medicine **9**.

Kenna, K. P., P. T. van Doormaal, et al. (2016). "NEK1 variants confer susceptibility to amyotrophic lateral sclerosis." Nat Genet **48**(9): 1037-1042.

Lattante, S., P. N. Doronzio, et al. (2021). "Novel variants and cellular studies on patients' primary fibroblasts support a role for NEK1 missense variants in ALS pathogenesis." Human molecular genetics.

Little, J., J. P. Higgins, et al. (2009). "Strengthening the reporting of genetic association studies (STREGA): an extension of the STROBE Statement." Hum Genet **125**(2): 131-151.

Morgan, S., M. Shoai, et al. (2015). "Investigation of next-generation sequencing technologies as a diagnostic tool for amyotrophic lateral sclerosis." Neurobiol Aging **36**(3): 1600 e1605-1608.

Naruse, H., H. Ishiura, et al. (2019). "Burden of rare variants in causative genes for amyotrophic lateral sclerosis (ALS) accelerates age at onset of ALS." J Neurol Neurosurg Psychiatry **90**(5): 537-542.

Nguyen, H. P., S. Van Mossevelde, et al. (2018). "NEK1 genetic variability in a Belgian cohort of ALS and ALS-FTD patients." Neurobiol Aging **61**: 255 e251-255 e257.

Nishiyama, A., T. Niihori, et al. (2017). "Comprehensive targeted next-generation sequencing in Japanese familial amyotrophic lateral sclerosis." Neurobiology of aging **53**: 194 e191-194 e198.

Pozzi, L., F. Valenza, et al. (2017). "TBK1 mutations in Italian patients with amyotrophic lateral sclerosis: genetic and functional characterisation." J Neurol Neurosurg Psychiatry **88**(10): 869-875.

Richards, S., N. Aziz, et al. (2015). "Standards and guidelines for the interpretation of sequence variants: a joint consensus recommendation of the American College of Medical Genetics and Genomics and the Association for Molecular Pathology." Genet Med **17**(5): 405-424.

Scarlino, S., T. Domi, et al. (2020). "Burden of Rare Variants in ALS and Axonal Hereditary Neuropathy Genes Influence Survival in ALS: Insights from a Next Generation Sequencing Study of an Italian ALS Cohort." Int J Mol Sci **21**(9).

Shu, S., X. Lei, et al. (2018). "Mutation screening of NEK1 in Chinese ALS patients." Neurobiol Aging **71**: 267 e261-267 e264.

Tsai, Y. S., K. P. Lin, et al. (2020). "Hand-onset weakness is a common feature of ALS patients with a NEK1 loss-of-function variant." Ann Clin Transl Neurol **7**(6): 965-971.

Zhang, K., Y. Lu, et al. (2020). "NEK1 and GRN mutations coexist in a sporadic Chinese Hui descent ALS patient." Amyotroph Lateral Scler Frontotemporal Degener **21**(7-8): 624-626.
